# Acetolysis of waste polyethylene terephthalate for upcycling and life-cycle assessment study

**DOI:** 10.1038/s41467-023-38998-1

**Published:** 2023-06-05

**Authors:** Yuantao Peng, Jie Yang, Chenqiang Deng, Jin Deng, Li Shen, Yao Fu

**Affiliations:** 1 CAS Key Laboratory of Urban Pollutant Conversion, Anhui Province Key Laboratory of Biomass Clean Energy, Department of Applied Chemistry, University of Science and Technology of China, Hefei, Anhui 230026 PR China; 2 Utrecht University, Copernicus Institute of Sustainable Development, Utrecht, The Netherlands

**Keywords:** Chemical engineering, Sustainability, Polymers

## Abstract

To reduce environmental pollution and reliance on fossil resources, polyethylene terephthalate as the most consumed synthetic polyester needs to be recycled effectively. However, the existing recycling methods cannot process colored or blended polyethylene terephthalate materials for upcycling. Here we report a new efficient method for acetolysis of waste polyethylene terephthalate into terephthalic acid and ethylene glycol diacetate in acetic acid. Since acetic acid can dissolve or decompose other components such as dyes, additives, blends, etc., Terephthalic acid can be crystallized out in a high-purity form. In addition, Ethylene glycol diacetate can be hydrolyzed to ethylene glycol or directly polymerized with terephthalic acid to form polyethylene terephthalate, completing the closed-loop recycling. Life cycle assessment shows that, compared with the existing commercialized chemical recycling methods, acetolysis offers a low-carbon pathway to achieve the full upcycling of waste polyethylene terephthalate.

## Abstract (web summary)

The recycling of polyethylene terephthalate is of utmost importance to reduce environmental pollution and reliance on fossil resources however, the existing methods do not process colored or blended polyethylene terephthalate materials. Here, the authors demonstrate the acetolysis of waste polyethylene terephthalate into terephthalic acid and simultaneous acidic degradation of dyes and additives

## Introduction

Plastics have become an important material in the modern global consumer economy, and the production is estimated to increase to around 12 billion tons by 2050^1–3^. As a large class of plastics, polyethylene terephthalate (PET) is widely used in various containers, packaging materials, and textiles^4,5^, with a production scale of more than 70 million tons per year^6^. PET does not degrade in nature and has a low recycling rate, especially if it is colored or blended, resulting in a large amount of plastic waste^7,8^. Effective recycling or upcycling methods for waste and contaminated plastic are being intensively developed^9,10^.

At present, post-consumer PET is mainly recycled by mechanical methods, but this will lead to the deterioration of the material structure and performance, and typically low-value products can be produced for downcycling^5,11^. Compared with mechanical recycling, chemical recycling of PET breaks down the polymer into monomers, which can be used again for polymerization or other more valuable products^12^. Common chemical methods for recycling waste PET include methanolysis^13–15^, glycolysis^16–20^, neutral hydrolysis^21–24^, alkaline hydrolysis^25,26^, acidic hydrolysis^27–30^. Recently, Gregg T. Beckham et al. have made a development in the enzymatic hydrolysis of PET^31^. However, the necessary amorphous PET pretreatment step and pH regulation make this process no more environmentally friendly than the current virgin PET^32^. In addition, the methods of hydrogenolysis of PET reported in recent years^33–35^ provide an efficient way to treat polyester. However, according to the cleavage of the ester group via hydrogenolysis, these methods always lead to a moiety of the depolymerization products being converted into other substances (i.e., either terephthalic acid converted into 1,4-benzenedimethanol, or ethylene glycol converted into ethane) that cannot be recovered as monomers.

Among these chemical methods, methanolysis and glycolysis, as the industrialized processes, are less efficient when the dyed feedstock (for example, green flake) is input, which makes the above methods highly sensitive to the purity of the feedstock of waste PET^5,36^. Besides, both acid and alkaline hydrolysis use strong acids or strong bases as solvents, which will cause serious corrosion to the equipment and produce a large amount of waste in the post-processing stage. Due to these inadequacies of existing chemical recycling methods, colored PET bottles and PET textiles are regarded as unrecyclable back into high-quality, food-grade PET for upcycling^31,37,38^. Hence, it is of great importance to develop an effective and sustainable method to upcycle PET waste.

Acetolysis, as a carboxylic acidolysis (the opposite of alcoholysis), is the exchange reaction between carboxylic acid and ester. Although the depolymerization of PET with acetic acid (weak acid) to obtain TPA (strong acid) is chemically unfavorable, the solubility of TPA in acetic acid is also extremely low. Therefore, if the product is removed simultaneously, the reaction equilibrium will be shifted towards the product. Based on this characteristic of TPA in acetic acid, we developed a new strategy to depolymerize waste PET into terephthalic acid (TPA) and ethylene glycol diacetate (EGDA) through acetolysis and to achieve PET re-polymerization via reverse reaction of acetolysis for upcycling. In this strategy, acetic acid has three main advantages: (i) Since the solubility of TPA in acetic acid is very low (1.08 × 10^−2^ g/100 g@32 °C, 3.86 g/100 g@240 °C), which not only ensures the complete decomposition of PET but also allows TPA to be crystallized out in a high-purity form. (ii) Acetic acid can dissolve or decompose other common components in PET materials (such as dyes, additives, blending ingredients, etc.) to ensure the separation of TPA from these impurities. (iii) As the solvent for industrial oxidation of *p*-xylene to produce TPA^39^, acetic acid can be directly applied to existing production devices. Additionally, to compare acetolysis with other existing chemical recycling, we designed a path to complete the closed-loop recycling PET. An attributional life-cycle assessment (LCA) was conducted to assess the environmental impacts of acetolysis and compare it with other industrial recycling methods.

## Results

### Acetolysis of PET plastics

**Fig. 1:**
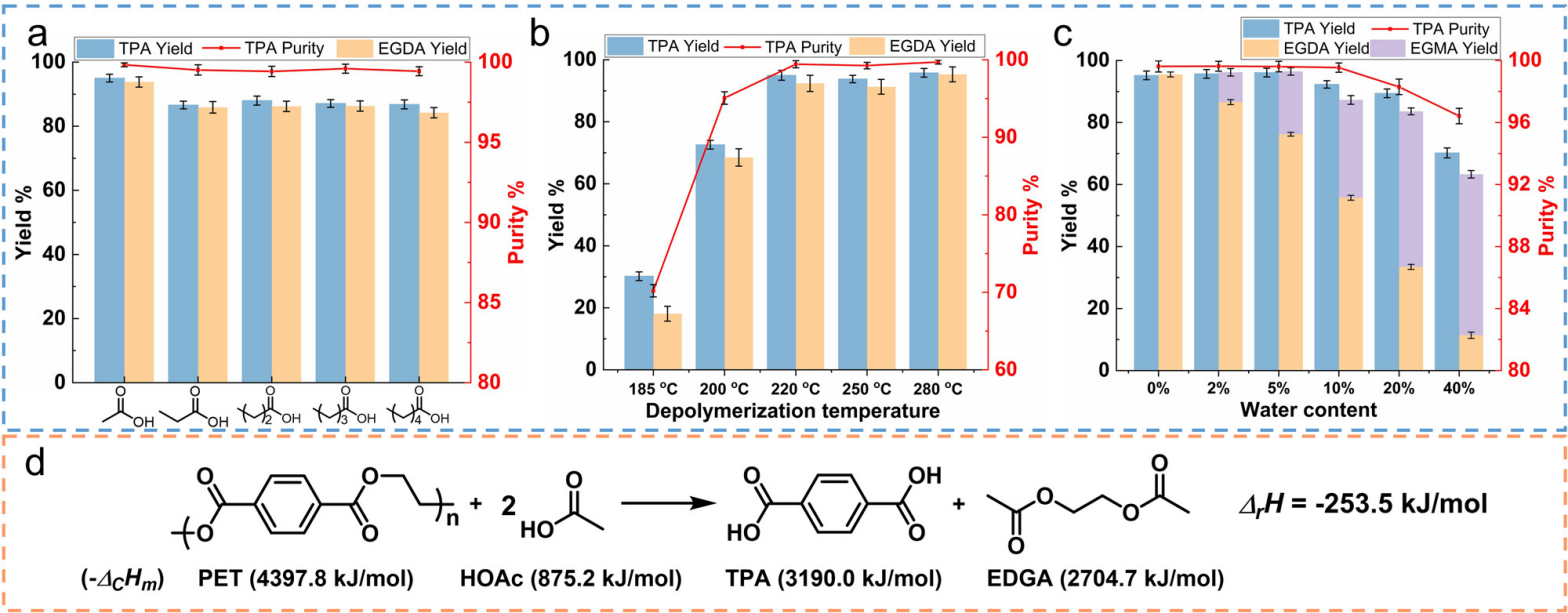
Acetolysis of PET. **a** Effects of different carboxylic acids on the acetolysis of PET. **b** Effects of different temperatures on the acetolysis of PET. **c** Effects of different moisture contents on the acetolysis of PET. **d** Reaction enthalpy of the acetolysis of PET. The combustion enthalpy of PET is from Supplementary ref. ^13^. Combustion enthalpy data for other substances are from ASPEN Plus. Error bars indicate the standard deviation of the depolymerization results obtained from three independent replicates.

As the solvent used in the industrial production of TPA, acetic acid was selected, and it was more effective in depolymerizing PET than other carboxylic acids due to it having the strongest acidity (Fig. 1a and Supplementary Table 2). Through acetolysis, used PET bottle flakes could be completely depolymerized in two hours at 280 °C, and high-purity TPA (95.8% yield, over 99.7% purity) and EGDA (95.3% yield, over 98.0% purity) could be obtained. Our study found that the acetolysis of PET was very sensitive to the decrease in the reaction temperature (Fig. 1b and Supplementary Table 3). When the temperature was below 220 °C, satisfactory results could not be obtained even if the reaction time was extended to 18 h. A certain amount of strong acid (more than 10% triflic acid) can significantly accelerate the degradation of PET at 180 °C (Supplementary Table 4), but the addition of acid not only causes trouble to the separation of EGDA but also aggravates the environmental impact. On the other hand, since the acetolysis of PET is an exothermic reaction (Fig. 1d), which requires a little energy to maintain the reaction temperature, the acetolysis of PET is not an energy-intensive process. For the above reasons, 280 °C was chosen as the optimal reaction temperature without any catalyst in the acetolysis process. Additionally, a certain amount of water will not have adverse effects on PET degradation but will make part of EGDA to ethylene glycol monoacetate (EGMA) conversion (Fig. 1c and Supplementary Table 6). This showed that the process did not have strict requirements for water in the pretreatment stage.

**Fig. 2:**
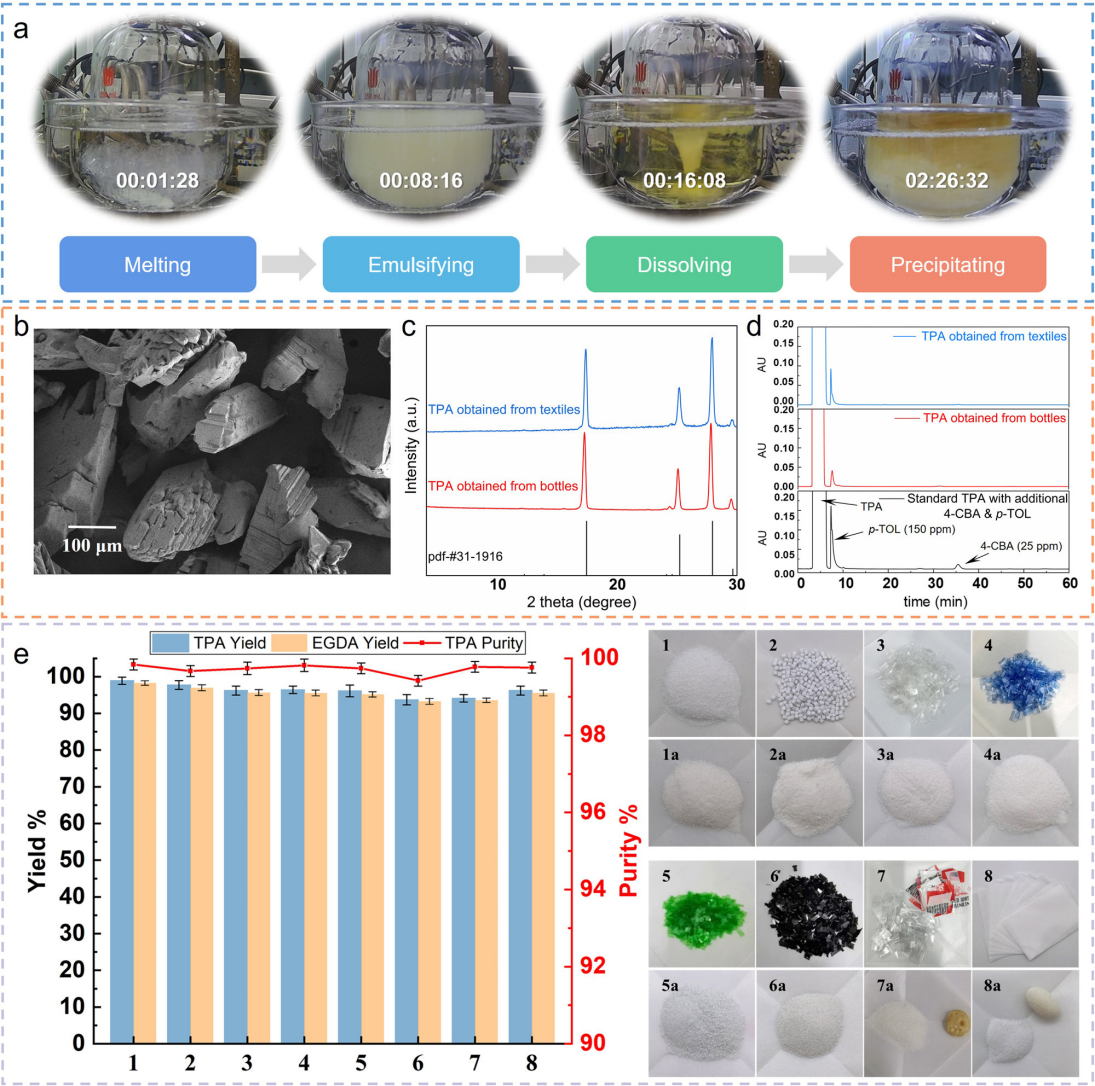
Results of chemical degradation of PET through acetolysis. **a** The reaction process of acetolysis of PET bottles. **b** SEM pattern of TPA obtained from waste PET bottles through acetolysis. **c** XRD pattern of TPA obtained from waste PET bottles and textiles. **d** HPLC spectra of TPA obtained from waste PET bottles and textiles. **e** Universality of PET degraded by acetolysis, 1–8 are photos of PET powder, particles, transparent bottle, blue bottle, a green bottle, black tray, physical mixing of PET and PP, PET/PP composite films, 1a–8a are the photos of terephthalic acid obtained by acetolysis of the above materials. Error bars indicate the standard deviation of the depolymerization results obtained from three independent replicates.

To better understand the depolymerization behavior of PET in carboxylic acid, a surveillance movie was recorded (see Supplementary Movie 1 for details). The movie showed that the depolymerization of PET in carboxylic acids underwent a melting-dissolving-precipitating process (Fig. 2a). At first, the PET melted rapidly in carboxylic acid, gradually forming an evenly dispersed emulsion. Then the melted PET depolymerized into small molecular weight oligomers that could be dissolved in carboxylic acids. Finally, the oligomers further depolymerized to form TPA and crystallized out in the system. Whereas TPA was continuously precipitated from the acetic acid system as a *p*-type flake crystal according to scanning electron microscope (SEM) and X-ray powder diffraction (XRD) (Fig. 2b, c and Supplementary Fig. 8). This process, unlike hydrolysis, was particularly efficient as the depolymerization of oligomers dissolved in carboxylic acid was a homogeneous reaction. The precipitation of TPA not only effectively drives the chemical equilibrium of depolymerization but also makes the separation of products simpler. Additives such as pigments and dyes were dissolved in organic acids without precipitating along with TPA. In addition, impurities such as *p*-toluic acid (*p*-TOL) and 4-carboxybenzaldehyde (4-CBA) produced by the incomplete oxidation of *p*-xylene were in trace amounts in the PET depolymerization product (Fig. 2d), which fully complies with the GB^40^ and the ASTM^41^ standard for polymerization grade TPA. These guaranteed the purity of TPA obtained by acetolysis of PET, and it also could be proved by titration analysis, high-performance liquid chromatography (HPLC), Fourier transform infrared spectrum (FTIR), ultraviolet–visible spectroscopy (UV–VIS), etc. (Supplementary Fig. 8).

Figure 2e illustrated that acetolysis had a strong tolerance to the source of waste PET plastics (see Supplementary Table 7 for more examples). PET plastics like powder, particles, or bottle flakes (colorless, colored, or even black) could be quantitatively depolymerized to TPA and EGDA. For PET flakes mixed with polyethylene (PE) labels and PET-polypropylene (PP) composite films, PET decomposed into TPA sinking, whereas PE and PP melted, agglomerated, and floated on the solution after acetolysis (Supplementary Fig. 9). However, with a similar density to PET, polyvinyl chloride (PVC) will be partially decomposed but not affect the depolymerization of PET^42^. In addition, other polyester plastics such as poly (ethylene terephthalateco-1,4-cylclohexylenedimethylene terephthalate) (PETG), polybutylene terephthalate (PBT), polyethylene naphthalenediate (PEN), and polyethylene 2,5-furandicarboxylate (PEF) could be degraded by this process with corresponding monomers obtained in high yield (Supplementary Table 9).

### Acetolysis of PET textiles

PET fiber is the highest market-shared chemical fiber with the most important application of PET products (ca. 80%). Compared with bottle-grade PET, fiber-grade PET generally has a lower degree of polymerization, and its textiles are generally more complex than bottles^43^. For PET textiles, the current physical recycling method can only lead to downcycling due to the gradually decreasing degree of polymerization, whereas the current chemical recycling methods are unable to achieve up cycling due to the difficulty of separating and purifying the depolymerized products^31,39,44^.

**Fig. 3:**
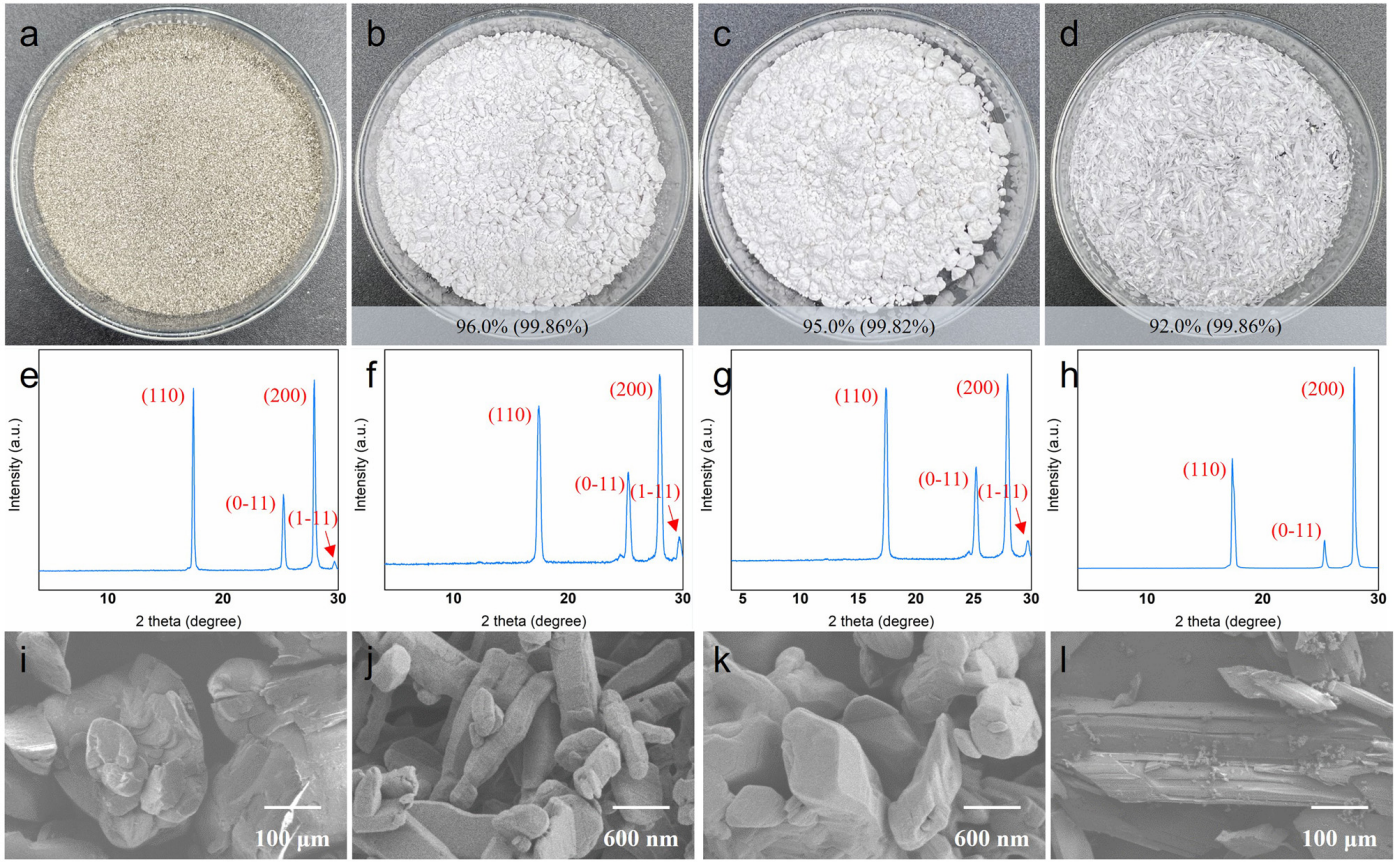
Decolorization of dark TPA. **a** Dark TPA obtained directly from acetolysis of Variegated clothes. **b** Product obtained by decolorizing dark TPA (sample a) in an alkaline solution with activated carbon. **c** Product obtained by decolorizing dark TPA (sample a) in N, N-dimethylacetamide with activated carbon. **d** Product obtained by decolorizing dark TPA (sample a) in hot water (280 °C) with activated carbon. The yield of decolorization (purity after decolorization) is shown in the figure, and the chromaticity after decolorization is all 5 Hazen. **e** XRD pattern of dark TPA (sample a). **f** XRD pattern of TPA decolorized with an alkaline solution. **g** XRD pattern of TPA decolorized with N, N-dimethylacetamide. **h**, XRD pattern of TPA decolorized with hot water. **i** SEM image of dark TPA. **j** SEM image of TPA decolorized with an alkaline solution. **k** SEM image of TPA decolorized with N, N-dimethylacetamide. **l** SEM image of TPA decolorized with hot water. All dark products obtained by acetolysis of waste PET material were easily decolorized in the described ways, including colored plastic bottles and black trays.

We applied the acetolysis method and successfully depolymerized used PET textiles (Table 1, Supplementary Figs. 10–12). Since some dyes or other organic impurities are partially decomposed and carbonized, the depolymerized product TPA resulted in a darker color. These dark TPAs could be decolorized by activated carbon after being dissolved in alkaline (Method 1) or amide (Method 2). Applying the two decolorization methods, white PET with qualified chromaticity (Hazen < 10) could be obtained^41^. The third decolorization method is to alter the high-temperature (280 °C) pure water recrystallization with activated carbon. The appearance of the product was also significantly improved (Fig. 3). Based on the LCA, the use of strong bases and acids in Method 1 and the recovery of amide in Method 2 could lead to a huge environmental impact. Decolorization by recrystallization in hot water (Method 3) offers the least environmental impact of the three (Supplementary Fig. 54).

**Table 1:** Degradation of real textiles by acetolysis^a^

^a^Reaction conditions: PET Textile fragments (60 g), acetic acid (300 mL) reaction temperature (280 °C), reaction time (2 h).

^b^bDark TPA was decolored in an alkaline solution with activated carbon.

^c^Yields are calculated for decolorized TPA based on PET textile raw materials.

In addition, for textiles made from PET blended with other polymers, the method of acetolysis worked effectively. Since acetic acid can decompose and transform many synthetic fibers at high temperatures to form acetic acid-soluble depolymerization products (e.g., nylon 6 is decomposed into 6-acetylaminocaproic acid; nylon 66 is decomposed into adipic acid and diacetyl hexamethylene diamine; polyacrylonitrile fiber is converted into acetamide; spandex is decomposed into acetanilide, Supplementary Table 10), so it does not affect the separation of TPA. Meanwhile, because these synthetic fiber decomposition products often have high boiling points, the separation and purification of EGDA via distillation are straightforward. It is worth mentioning that EGDA, as a high-value-added compound with a strong dissolving ability, can be used as a green solvent for ink coatings, which makes the acetolysis process more economical.

### LCA for closed-loop upcycling PET via acetolysis process

**Fig. 4:**
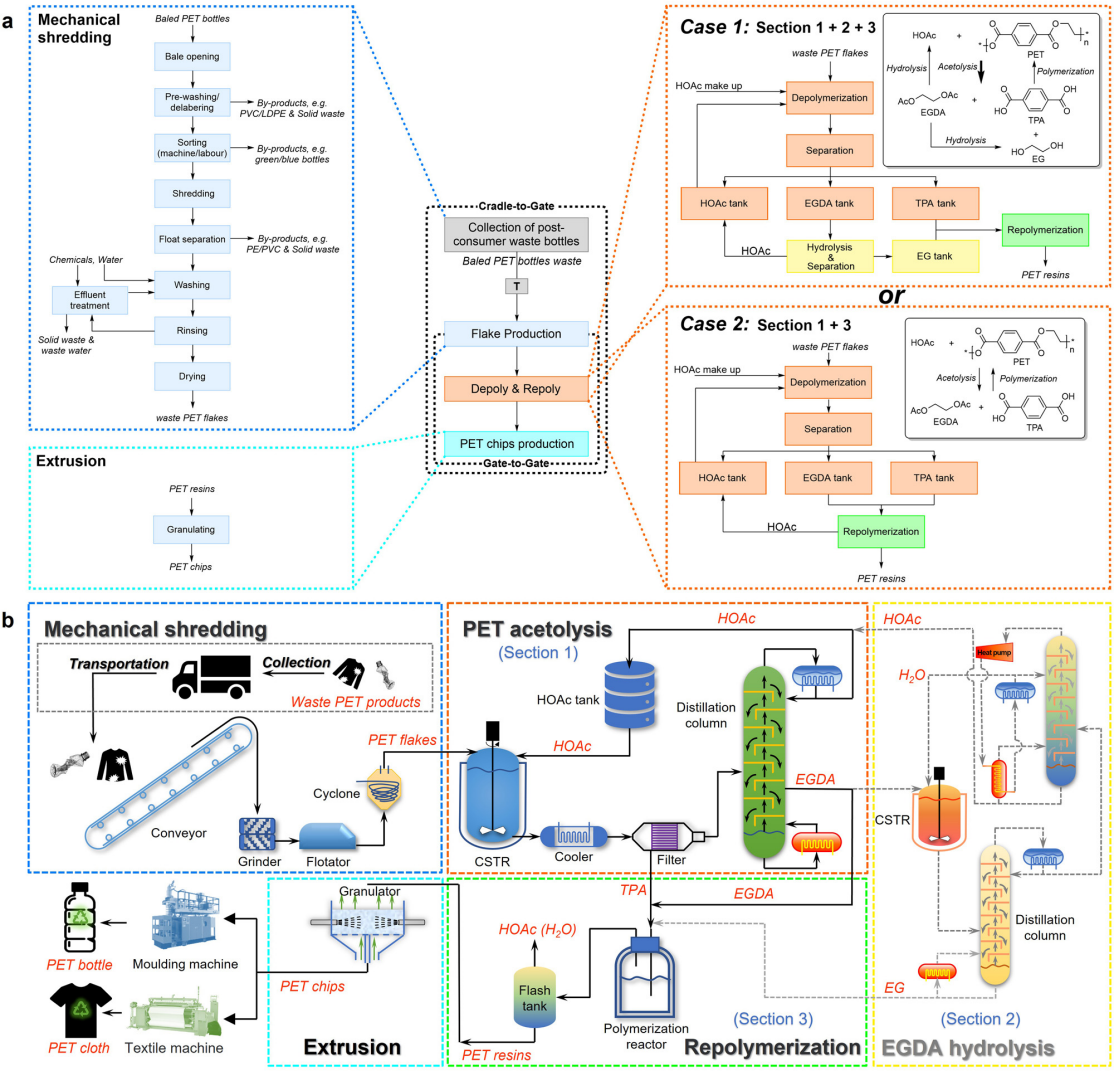
The process for closed-loop upcycling PET via acetolysis. **a** Cradle-to-factory gate system boundary of closed-loop upcycling PET via an acetolysis process based on the cut-off approach (illustrated by taking waste PET bottles as an example). **b** Schematic diagram of closed-loop upcycling PET via the acetolysis (decolorization not shown).

We applied an LCA to compare the greenhouse gas emissions of the acetolysis method with other existing industrial methods for recycling PET. Since the subsequent use and disposal of various PET products vary greatly, the system boundary of this LCA is set as cradle-to-gate. To simplify the analysis^44–47^, the LCA of PET recycling is based on the cut-off approach (Fig. 4a). Herein, the foreground process data are obtained by experiments and Aspen simulations. Background process data such as the production of tap water, electricity, gas, and basic chemicals come from ecoinvent v3.7^48^, while the pretreatment of waste PET and extrusion of PET resins are derived from literature^49^. See detailed information in Supplementary Table 9.

The process for upcycling PET from waste PET via acetolysis is shown in Fig. 4b, which includes five units: (i) mechanical shredding of waste PET (including collection and transportation); (ii) acetolysis of PET flakes to produce TPA and EGDA (Section “Introduction”); (iii) hydrolysis of EGDA to EG (Section “Results”); (iv) repolymerizing of EG (EGDA) and TPA to PET resins (Section “Discussion”); and (v) extrusion of PET resins to chips. When PET feedstocks are colored bottles or textiles, the decolorization unit is also taken into account. The first and last units are physical conversions. Further, in the mechanical shredding section, we assume dealing with bottles is very similar to dealing with textiles. The chemical processes composed of the remaining units (Sections “Introduction” to “Discussion” and decolorization in specific scenarios) were simulated on an industrial scale with an annual treatment of 100,000 tons of waste PET, using Aspen Plus V11 to obtain the mass balance and energy consumption. The global warming potential (GWP) and non-renewable energy use (NREU) of the process were calculated by OpenLCA.

**Fig. 5:**
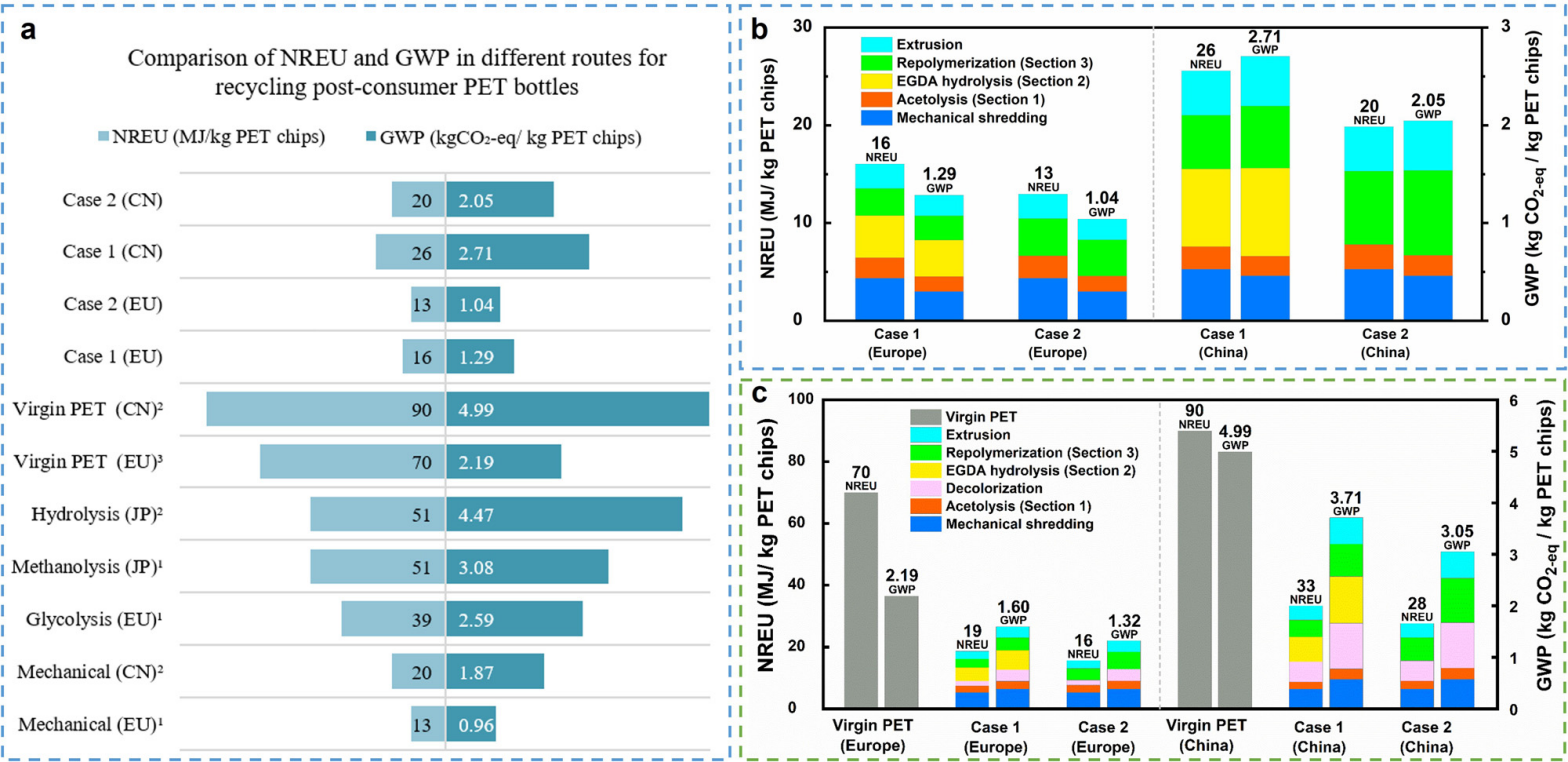
LCA results for closed-loop upcycling PET via acetolysis. **a** Comparison of NREU and GWP in different routes for recycling post-consumer PET bottles^1^. Ref. ^2,45^. Ref. ^3,50^. Ref. ^51^. EU: Europe, CN: China, JP: Japan. **b** Contribution analysis of NREU and GWP via acetolysis of post-consumer PET bottles (detailed data are listed in Supplementary Table 12). **c** Contribution analysis of NREU and GWP via acetolysis of post-consumer PET textiles (detailed data are listed in Supplementary Table 14).

The results (Fig. 5a) show that, compared with the process for preparing virgin-PET from the fossil resource, the NREU and GWP of the acetolysis process presented by Case 1 (Fig. 4a) could be reduced by over 70% and 40%, respectively, for both product systems assuming Europe or China as the background in Case 1. Compared to the literature data reported for chemical recycling methods for PET, acetolysis of PET also offers substantially lower GWP impacts (Fig. 5a). The breakdown of the results showed that section “Results” (EGDA hydrolysis) contributed the most GWP and NREU (Fig. 5b), especially based on China. In Case 2, hydrolysis of EGDA (Section “Results” in Fig. 4a) is omitted. EGDA is directly condensed with TPA and polymerized into PET by removing acetic acid to further simply the downstream design. The experimental results show that EGDA can indeed replace EG to produce PET with similar thermal properties and relatively low molecular weight (Supplementary Note 3). Due to the omission of the hydrolysis section, LCA results showed that PET produced in a closed-loop cycle via case 2 could further reduce GWP and NREU, and the impacts are comparable with those of the mechanical method reported in the literature (Fig. 5). This method provides a feasible and low-environmental impact solution for upcycling waste PET.

Furthermore, we also did the LCA for the recycling of post-consumer PET textiles via acetolysis (Fig. 5c). To the best of our knowledge, there is no publicly available LCA on chemical recycling of waste PET textiles into granulates to date. Therefore, we could only compare the results with fossil-based PET. Similarly, Europe and China are chosen to highlight the difference caused by background systems. From Fig. 5c, we can see that the environmental impacts of both Case 1 and Case 2 are lower than those of the fossil-based route for both EU and CN. The LCA results show that acetolysis of PET offers a very promising, low-carbon technological solution to upcycle used PET textiles.

## Discussion

In this work, we developed an acetolysis process to depolymerize used PET bottles and textiles into basic chemical building blocks and then re-polymerize them again into PET. Our approach is to degrade waste PET in acetic acid to obtain high-purity TPA and EGDA with yields over 90%. And the acetolysis can deal effectively with impurities, dyes, and other components (polyethylene or polypropylene or nylon, etc.) present in waste PET. We have also designed two schemes of closed-loop recycling of PET. The environmental impacts were assessed through an LCA. The LCA results showed that PET produced by acetolysis could significantly reduce GWP (by at least 40%) and NREU (by at least 70%) compared to virgin PET. Under optimal conditions, e.g., optimized heat integration in process design, acetolysis can offer a low-carbon upcycling technology solution compared to the existing, commercialized chemical recycling methods for waste PET. It should be noted that this LCA study only focuses on GWP and NREU. More environmental impacts (*e.g*., acidification, eutrophication, ecotoxicity) are not included due to a lack of available counterpart materials. So, future researches need to address other environmental impacts associated with PET chemical recycling. Briefly, this work provides a promising and sustainable way for upcycling PET waste and reducing dependence on fossil resources.

## Methods

### Polyester materials, chemicals, and reagents

In all experiments, PET fragments were obtained from various PET products, including transparent and colored, mono- and multi-layer post-consumer PET lunch boxes and trays, and PET water bottles without caps and labels. These materials were simply washed with tap water, dried, and cut into approximately 3 × 3 mm fragments for experiments. PET textiles fragments were donated by the author and his collaborators. PEF particles (yellow solid particles, average molecular weight > 30,000) and PEN polyester (pressure-sensitive silicone tape) were generously gifted by Hefei Leaf Biotechnology Co., Ltd. PETG polyester (3D printing consumables) was purchased in the Lambo flagship store. These materials were cut into fragments (or particles). PBT powder (300 mesh) was purchased from the Hongyuan polymer supply chain. PVC powder was purchased from Suyuan plastic raw material mall. TPA (99%), *p*-TOL (98%), and 4-CBA (98%) were purchased from Adamas Reagent Co., Ltd. Other chemicals are commercially available and utilized without further treatment.

### Acetolysis of PET plastics

A typical acetolysis process was shown as follows. 60.0 g PET flakes and 300 mL glacial acetic acid were mixed in a 500 mL titanium hydrothermal reactor. Then the hydrothermal reactor was placed in a homogeneous reactor for depolymerization at a constant temperature of 280 °C for 2 h. After the reaction, the hydrothermal reactor was cooled down to room temperature. The solid products were obtained by filtration and were washed three times with pure water and dried in an oven. The TPA yield was calculated by Eq. (1). The purity of TPA was tested by acid-base titrations according to the National Standard of the People’s Republic of China GB/T 30921.5-2016.

1 $${{{{{\rm{TPA}}}}}}\,{{{{{\rm{yield}}}}}}\,\left(\%\right)=	\frac{{{{{\rm{{Produced}}}}}}\,{{{{\rm{{TPA}}}}}}\,{amount}\,(g)}{{{{{\rm{Theoretically}}}}}\,{produced}\,{TPA}\,{amount}\,\left(g\right)}\times 100\left(\%\right) \\=	\frac{{{{{\rm{TPA}}}}}\,{qunality}\,{by}\,{obtained}\,\left(g\right)}{\frac{{{{{\rm{PET}}}}}\,{amount}\,(g)}{192.13(\frac{{{{{\rm{g}}}}}}{{{{{\rm{mol}}}}}})}\times 166.13(\frac{{{{{\rm{g}}}}}}{{{{{\rm{mol}}}}}})}\times 100(\%)$$

The products in the liquid phase were analyzed by GC equipped with a flame ionization detector (FID) for quantitative analysis. The yield of EGDA was calculated by Eq. (2):

2 $${{{{{\rm{EGDA}}}}}\,{yield}}\,\left(\%\right)=	\frac{{{{{\rm{Produced}}}}}\,{EGDA}\,{amount}\,(g)}{{{{{\rm{Theoretically}}}}}\,{Produced}\,{EGDA}\,{amount}\,\left(g\right)}\times 100\left(\%\right) \\=	\frac{{{{{\rm{EGDA}}}}}\,{amount}\,{qunatified}\,{by}\,{GC}\,\left(g\right)}{\frac{{{{{\rm{PET}}}}}\,{amount}\,(g)}{192.13(\frac{{{{{\rm{g}}}}}}{{{{{\rm{mol}}}}}})}\times 146.14(\frac{{{{{\rm{g}}}}}}{{{{{\rm{mol}}}}}})}\times 100(\%)$$

### Acetolysis of PET textiles

A typical acetolysis for textiles process was shown as follows. 60.0 g PET textile fragments and 300 mL glacial acetic acid were mixed in a 500 mL titanium hydrothermal reactor. Then the hydrothermal reactor was placed in a homogeneous reactor for depolymerization at a constant temperature of 280 °C for 2 h. After the reaction, the hydrothermal reactor was cooled down to room temperature. The products were separated and analyzed as previously shown.

### Decolorization of dark TPA

Decolorization of black TPA was carried out by activated carbon. *Method 1*: 20 g of dark TPA and 0.2 g of activated carbon were added to a 200 mL aqueous solution containing 20 g of sodium hydroxide and the mixture was refluxed for 20 min. The impurities were removed by hot filtration and white TPA was obtained by adding an equal amount of dilute sulfuric acid. *Method 2*: 40 g of dark TPA and 0.4 g of activated carbon were added to 200 mL of N, N-dimethylacetamide (DMAC) and the mixture was stirred at 150 °C for 20 min. The insoluble impurities were removed by hot filtration. The adductive crystals of TPA and DMAC selectively precipitated out of the filtrate. White TPA could be obtained by heating the crystals or adding water after filtration. *Method 3*: 40 g of dark TPA and activated carbon fiber felt (10 cm × 15 cm) were added to 200 mL of water and stirred for 30 min at 280 °C. Then cooled the reactor to room temperature slowly to obtain clean TPA crystals. All dark products obtained by acetolysis of waste PET materials were easily decolorized in the above-described ways.

### Polymerization of TPA and EGDA

A typical polymerization of TPA and EGDA process was shown as follows. TPA (100 g), EGDA (105 g, 1.2eq), and Sb_2_O_3_ (50 mg, 0.05 wt%) were mixed in a 500 mL custom-made titanium polyester reactor. The reactor was then filled with nitrogen and replaced 5 times with nitrogen, maintaining the polyester reactor pressure at 8 atm. The transesterification reaction takes place at 260–280 °C, and the pressure is controlled at about 8 atm. The progress of the reaction was assessed by the amount of acetic acid produced as a by-product. After more than 4 h, the transesterification reaction was completed. The polycondensation reaction started at 280 °C and a high vacuum of less than 200 Pa. The polycondensation reaction lasts for 3 h to obtain the desired polymer. The structure and properties were characterized by ^1^H NMR, DSC, and GPC, see detailed information in Supplementary Note 3.

### Characterization

Products in the liquid phase were detected by flame ionization detector gas chromatography (GC-FID, Shimadzu-Nexis GC-2030), which used HP-INNOWAX (30 m × 0.25 m × 0.25 mm) as a capillary column. High-performance liquid chromatography (HPLC) was performed using Agilent HC-C18, 4.6 × 250 mm column and Waters UV detector 2489 at 254 nm. Eluent: acetonitrile/water = 10:90 (volume ratio, containing 0.5% phosphoric acid). Nuclear magnetic resonance (NMR) spectroscopic measurements were carried out in deuterated dimethyl sulfoxide (DMSO-d6) or deuterated chloroform (CDCl_3_) on a Bruker Advance 400 (400 MHz) spectrometer for the product characterization. The morphology of the samples was observed with Zeiss Gemini 500 Schottky field emission gun SEM (2–5 kV). XRD data were acquired on a JEOL-2010 diffractometer using Cu Kα radiation at 4 ~ 30 degrees. **FTIR** was recorded on an FTIR/Raman Thermo Nicolet 8700 spectrometer in the wavenumber range of 3800 to 500 cm^−1^, with an instrument resolution of 0.1 cm^−1^. The UV–VIS absorption spectra were collected with a UV-1750 UV–vis spectrophotometer (Shimadzu, Japan). Differential scanning calorimetry (DSC) was used to detect the product of the polymerization. The sample (5 mg ± 0.5 mg) was heated and analyzed on DSC Q2000 using a heating program (30–300 °C, 10 °C/min). Molecular weight and molecular weight distributions were determined by gel permeation chromatography (GPC) equipped with hexafluoroisopropanol (HFIP) column and OPTILAB DSP interference refractometer as detector.

### Life-cycle assessment

The LCA analysis followed the ISO standard series 14040 and was conducted using OpenLCA 1.10.3. The aim is to identify the optimal acetolysis design to recycle PET, and to compare the results with virgin PET and other chemical recycling methods. The functional unit is 1 kg of amorphous PET resin in chips form. The system boundary is cradle-to-gate, which included: the collection and transportation of waste PET, the production of PET flakes, the whole processes of depolymerization and re-polymerization, and extrusion into PET chips. Two environmental impact categories were assessed, namely NREU and GWP. Two geographical scopes were investigated, namely China and Europe. The detailed data and assumptions can be found in Supplementary Information.

## Supplementary information

Supplementary Information

Peer Review File

Description of Additional Supplementary Files

Supplementary Movie 1

## Data Availability

The experiment data generated in this study are provided in the Supplementary Information. Additional data are available from the corresponding author upon request.
