# Supplementary material for: Acetolysis of waste polyethylene terephthalate for upcycling and life-cycle assessment study
Source: Nat Commun. 2023 Jun 5;14:3249. doi: 10.1038/s41467-023-38998-1 (PMC10241940; doi:10.1038/s41467-023-38998-1)
Supplement: Supplementary file 1 — Supplementary Information [file 41467_2023_38998_MOESM1_ESM.pdf]

Supplementary Information for

Acetolysis of waste polyethylene terephthalate for upcycling and life-cycle assessment study

Yuantao Peng[1], Jie Yang[1], Chenqiang Deng[1], Jin Deng[1]*, Li Shen[2]* and Yao Fu[1]*

[1]CAS Key Laboratory of Urban Pollutant Conversion, Anhui Province Key Laboratory of Biomass Clean Energy Department of Applied Chemistry, University of Science and Technology of China, Hefei, Anhui, 230026, PR China.

[2]Utrecht University, Copernicus Institute of Sustainable Development, Utrecht, the Netherlands.

*Corresponding author.

E-mail: dengjin@ustc.edu.cn

E-mail: L.Shen@uu.nl

E-mail: fuyao@ustc.edu.cn

Supplementary Notes

Supplementary Note 1: The kinetics of PET degradation by acetolysis

The experimental results indicated that the acetolysis of PET followed the first-order reaction. Its kinetic constant (k) value can be calculated by the following equation (1) derived from the integral rate law:

$$ln\frac{1}{1-y} = kt \qquad (1)$$

where y, k, and t are the depolymerization rate of PET (for simplicity, the yield of EGDA was used instead of the depolymerization rate of PET), the rate constant of the reaction, and the reaction time, respectively.

According to the depolymerization rate of PET at different temperatures (200 °C, 220 °C, 240 °C, 260 °C, 280 °C) and different times, the rate constant k at different temperatures could be obtained by linear fitting, and then ***Ea*** of the reaction could be obtained from Arrhenius equation (2):

$$lnk = lnA - \frac{Ea}{RT} \qquad (2)$$

where k, A, R, and T are the rate constant of the reaction, the pre-exponential factor, the gas constant (8.31 J/k mol), and the absolute temperature in Kelvin, respectively.

The effect of reaction temperature on k calculated by equation (1) was shown in Supplementary Figure 1a. The experimental data showed a good linear relationship at different temperatures (Pearson's r > 0.98). In addition, the k value increases significantly with the increase of depolymerization temperature, which showed that temperature is the key factor affecting PET degradation. Supplementary Figure 1b was an Arrhenius plot of apparent k with good linear correlation (Pearson’s r = 0.9985). Therefore, it was reasonable to assume that the acetolysis of PET follows first-order kinetics. According to the slope of the Arrhenius curve, the apparent activation energy of the reaction was 127.38 kJ / mol, which was comparable to the ***Ea*** of other acid hydrolysis [1, 2].

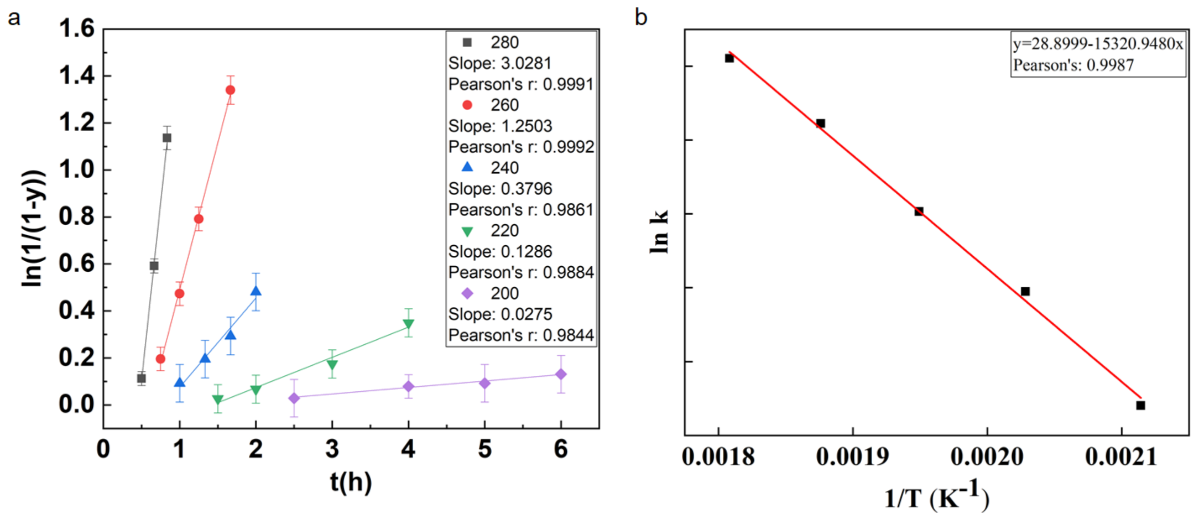

Supplementary Figure 1 Kinetic data of acetolysis. a, Relationship between ln(1/(1–y)) and t at different temperatures. **b,** Arrhenius curve of chemical degradation of PET through acetolysis. Error bars indicate the standard deviation of the depolymerization results obtained from three independent replicates.

Supplementary Note 2: Kilogram scale degradation of post-consumer PET plastic waste through acetolysis

Reaction equation:

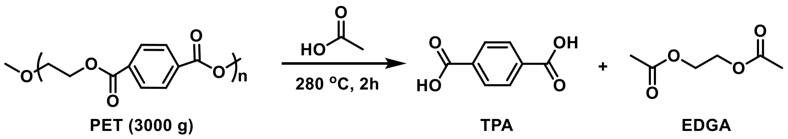

(3)

Reaction equipment: 20L Hastelloy-C276 mechanical stirring autoclave with sampling valve (working pressure range: 1-30 atm; working temperature range: r.t-350 °C)

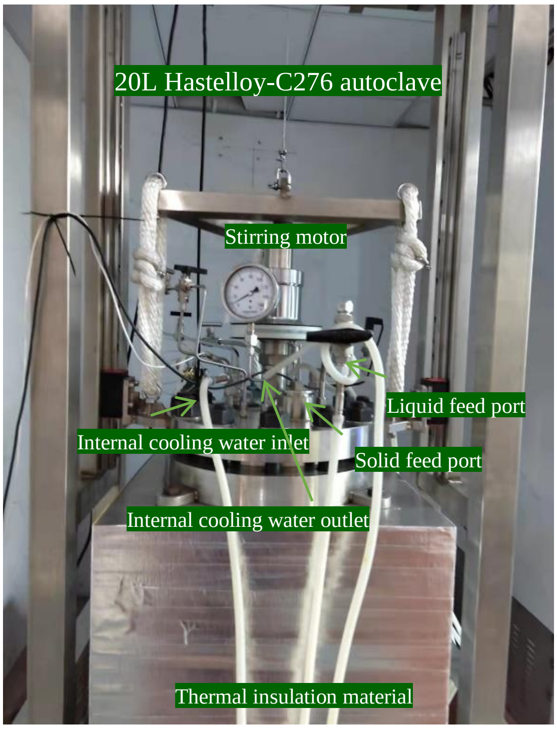

Supplementary Figure 2 Mechanical stirring autoclave for the acetolysis of PET.

Input form: Post-consumer PET plastic waste: 3000 g (15.6 mol)

Glacial acetic acid (purity ≥99.5%): 15 L

Reaction conditions: Reaction temperature: 280 ºC (from room temperature to 280 ºC at 5 ºC/min)

Reaction time: 2 hours

Stirring rate: 60 rpm

Operation process: The weighed post-consumer PET plastic waste (3000 g) and glacial acetic acid (15 L) were put into the autoclave in turn. After sealing the autoclave, mechanical stirring was started and the speed was controlled at 60 rpm. The autoclave was heated to 280 ºC for two hours. After the reaction, the autoclave was cooled down to room temperature with internal cooling water. The reaction liquid and products were taken out through the valve at the bottom of the autoclave.

Post-processing: Terephthalic acid was separated by suction filtration, then washed with water several times and dried in a drying oven. We finally obtained 2350g TPA with a yield of 90.60% (purity: 99.86%).

The acetic acid in the reaction solution was spun out under reduced pressure, and the vacuum degree was controlled at 15±5mmHg.

Then, the remaining liquid (2578g) was distilled under reduced pressure by an oil pump, the vacuum degree was controlled in the range of 2-4 mmHg, the fractions at different temperatures were collected, and analyzed by GC.

Fraction composition:

Fractions	Weight	HOAc	EGDA	Others
front cut fraction (≤ 60 ºC)	240g	197.3g (82.2%)	41.5g (17.3%)	1.2g (0.5%)
**main fraction (60 - 80 ºC)**	**2095g**	**35.6g (1.7%)**	**2057.3g (98.2%)**	**2.1g (0.1%)**
distillation residue	158g	0.8g (0.5%)	37.2g (23.5%)	120.0g (76.0%)
sum	2493g	233.7g (9.4%)	2136.0g (85.7%)	123.3g (4.9%)

Vacuum distillation loss: 2578g – 2493g = 85g

Yield of EGDA: 2136.0g / 2281.3g = 93.6%

Isolated yield of EGDA: 2057.3g / 2281.3g = 90.2% (purity: >98%)

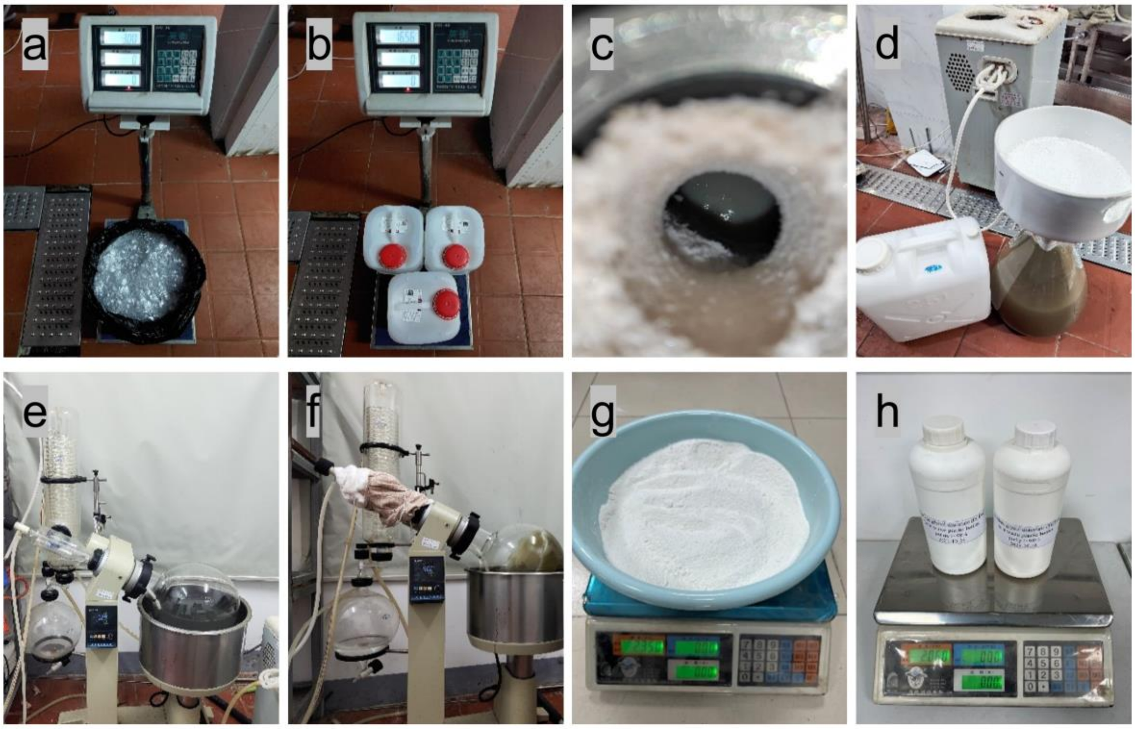

Supplementary Figure 3 Photos of Kilogram scale depolymerization of PET plastic. a, Waste PET fragments. **b,** Glacial acetic acid. **c,** After reaction. **d,** Suction filtration. **e,** Remove acetic acid. **f,** Vacuum distillation. **g,** Solid product (TPA). **h,** Liquid product (EGDA).

Supplementary Note 3: Polymerization of TPA and EGDA.

A typical polymerization of TPA and EGDA process was shown as follows (Supplementary Figure 4). TPA (100 g), EGDA (105 g, 1.2eq), and Sb_2O_3 (50 mg, 0.05 wt.%) were mixed in a 500 mL custom-made titanium polyester reactor (Supplementary Figure 5). The reactor was then filled with nitrogen and replaced 5 times with nitrogen, maintaining the polyester reactor pressure at 8 atm. The transesterification reaction takes place at 260-280 ºC, and the pressure is controlled at about 8 atm. The progress of the reaction was assessed by the amount of acetic acid produced as a by-product. After more than 4 hours, the transesterification reaction was completed. The polycondensation reaction started at 280 ºC and a high vacuum of less than 200 Pa. The polycondensation reaction lasts for 3 hours to obtain the desired polymer. The structure and properties were characterized by ^{1}H NMR, DSC, and GPC.

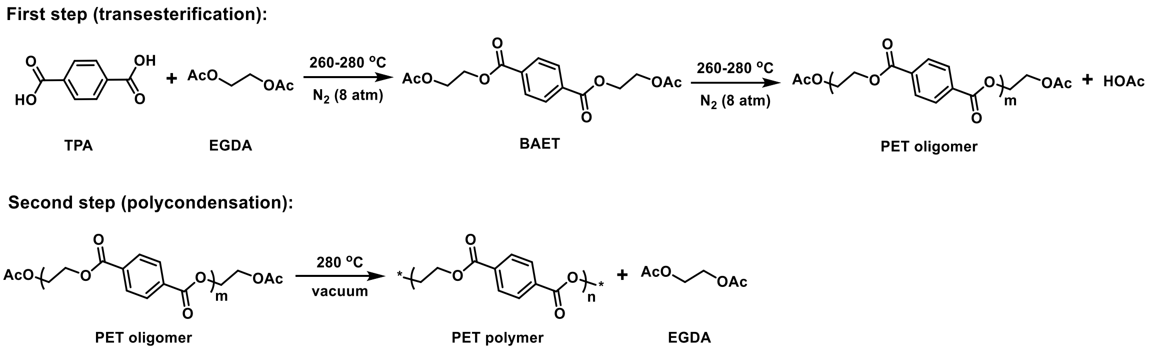

Supplementary Figure 4 Procedures for synthesizing PET with TPA and EGDA.

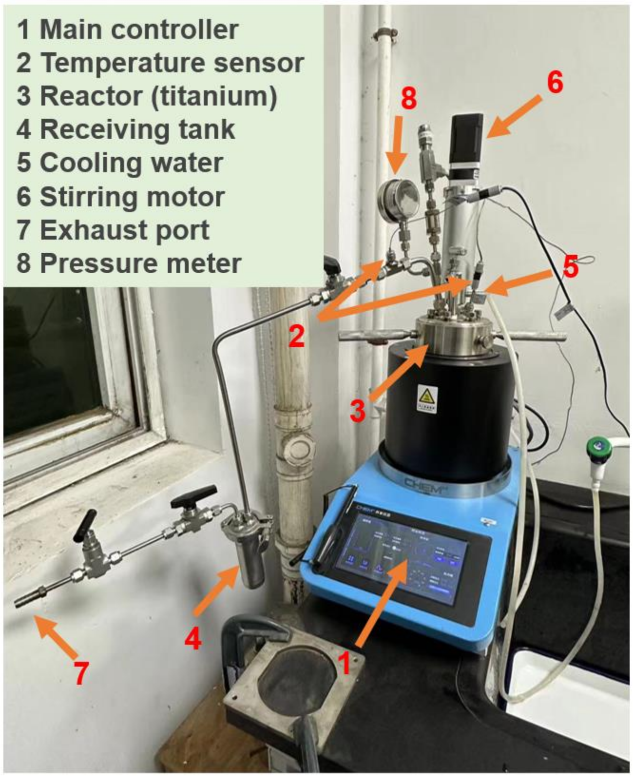

Supplementary Figure 5 Photo of custom-made titanium polyester reactor for PET re-polymerization.

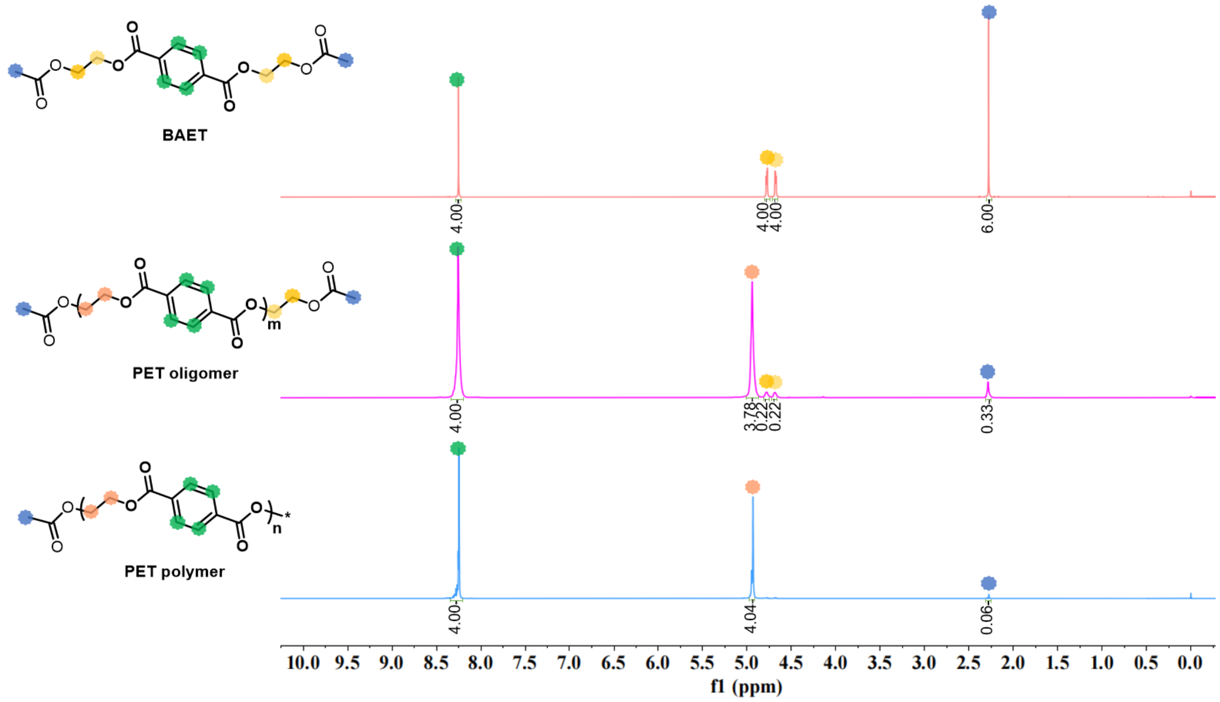

Supplementary Figure 6 ^{1}H NMR spectra of PET polymerization at different stages. (CF_3COOD)

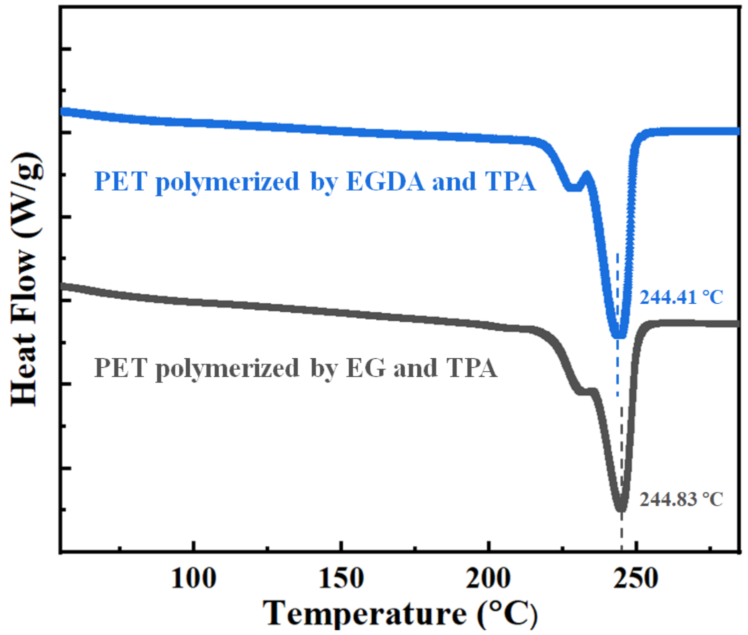

Supplementary Figure 7 DSC second heating scanning pictures of PET polymerized with different substrates.

Supplementary Table 1 Molecular weight of pet synthesized with different substrates.

	Mn[a] (kDa)	Mw[a] (kDa)	Mp[a] (kDa)	PD[a]
PET (Case1: EG+TPA)	37	67	56	1.8102
PET (Case2: EGDA+TPA)	18	41	34	2.3062

[a] Calculated by GPC in HFIP.

Supplementary Tables

Supplementary Table 2 Effects of different organic acids on PET degradation through acetolysis.

Entry	Organic acids	pKa [3]	TPA Yield/%	TPA Purity/%	EGDA Yield/%
1	Acetic acid	4.76	95.0 ± 1.2	99.83 ± 0.12	93.8 ± 1.6
2	Propionic acid	4.87	86.6 ± 1.2	99.51 ± 0.32	85.9 ± 1.8
3	*n*-Butyrate	4.83	88.0 ± 1.4	99.42 ± 0.32	86.2 ± 1.6
4	*n*-Valeric acid	4.83	87.1 ± 1.2	99.59 ± 0.28	86.3 ± 1.6
5	*n*-Caproic acid	4.85	86.8 ± 1.4	99.43 ± 0.28	84.2 ± 1.6

Reaction conditions: PET bottle fragments (60 g), organic acid (300 mL), reaction temperature (280 °C), reaction time (2 h).

Supplementary Table 3 Effect of reaction temperature on acetolysis of PET.

Entry	Temp./°C	Time/h	TPA Yield /%	TPA Purity/%	EDGA Yield/%
1	185	18	30.2 ± 1.4	70.2 ± 0.82	18.1 ± 2.4
2	200	18	72.6 ± 1.4	95.07 ± 0.78	68.5 ± 2.8
3	220	10	95.0 ± 1.6	99.43 ± 0.46	92.4 ± 2.6
4	250	8	93.8 ± 1.2	99.25 ± 0.42	91.3 ± 2.4
5	280	2	95.8 ± 1.4	99.72 ± 0.26	95.3 ± 2.4

Reactions conditions: PET bottle fragments (60 g), 300 mL acetic acid.

Supplementary Table 4 Depolymerization results in the presence of catalyst and corresponding photographs of TPA.

Entry	bottle flakes	Solvent	Catalyst	t/h	T/°C	TPA yield/%	EGDA yield/%
1	2.5 g	HOAc (25 mL)	HOTf (0.25 g)	1	180	70.2	65.8
2	2.5 g	HOAc (25 mL)	HOTf (1.00 g)	1	180	95.2	90.7

Supplementary Table 5 Effect of waste plastic concentration on acetolysis of PET.

Entry	Concentration of PET*/(% w/v)	TPA Yield/%	TPA Purity/%	EDGA Yield/%
1	5	93.3 ± 2.2	99.33 ± 0.36	90.3 ± 2.0
2	10	95.0 ± 1.6	99.43 ± 0.40	89.8 ± 1.8
3	20	95.5 ± 1.2	99.50 ± 0.32	95.6 ± 1.6
4	50	78.2 ± 3.6	97.35 ± 0.42	68.8 ± 3.4
5	100	75.2 ± 4.2	97.09 ± 0.48	68.2 ± 3.8

Reactions conditions: PET bottle fragments (1.25 ~ 25 g), 25 mL acetic acid, 280 °C, 2 h.

* Concentration of PET = [mass of PET (g) / volume of acetic acid (mL)] × 100%

Supplementary Table 6 Effect of water content on acetolysis of PET.

Entry	Water quality	TPA Yield/%	TPA Purity/%	EGA Yield/% (EGDA: EGMA)
1	-	95.2 ± 1.4	99.62 ± 0.36	95.5 (100%:0)
2	2% H_2O	95.7 ± 1.4	99.63 ± 0.32	96.2(90%:10%)
3	5% H_2O	96.1 ± 1.4	99.61 ± 0.34	96.5(79%: 21%)
4	10%H_2O	92.3 ± 1.2	99.54 ± 0.30	87.1(64%: 36%)
5	20%H_2O	89.4 ± 1.4	98.30 ± 0.52	83.6(40%: 60%)
6	40%H_2O	70.2 ± 1.6	96.42 ± 0.50	63.3(18%: 82%)

Reactions conditions: PET bottle fragments (5.0 g), solvent (25 mL acetic acid or aqueous solution of acetic acid with different water content), 280 °C, 2 h.

Supplementary Table 7 Acetolysis of waste PET from different sources.

Category	Sources	TPA	TPA Yield/%	EGDA Yield/%
PET powder	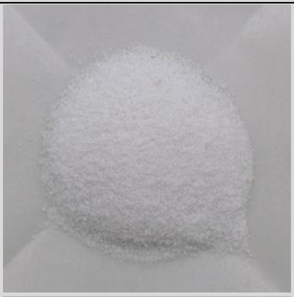	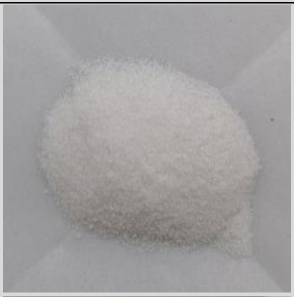	98.9 ± 1.0	98.3 ± 0.6
PET particle	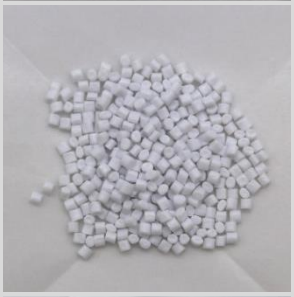	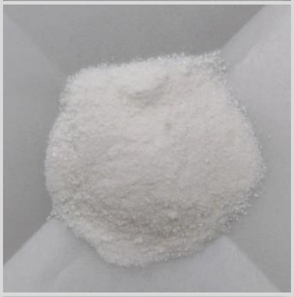	97.7 ± 1.2	97.0 ± 0.8
Transparent bottle	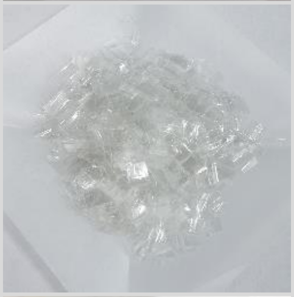	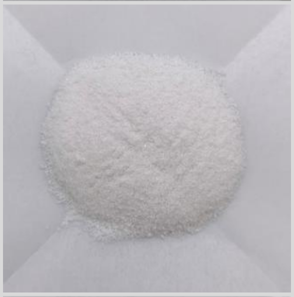	96.2 ± 1.2	95.7 ± 0.8
Blue bottle	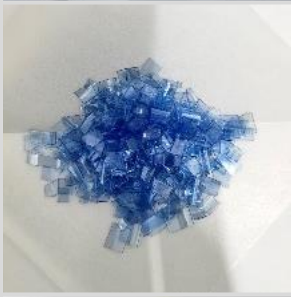	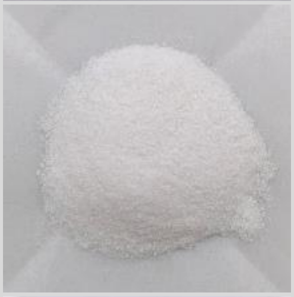	96.4 ± 1.0	95.6 ± 0.8
Green bottle	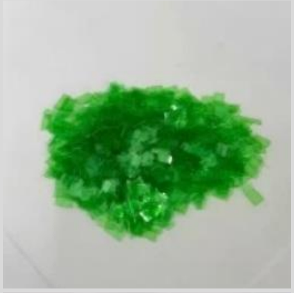	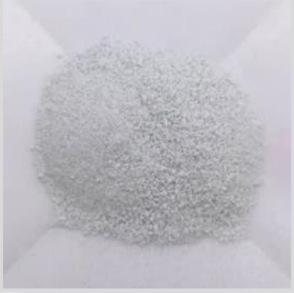	96.1 ± 1.6	95.2 ± 0.7
brown bottle	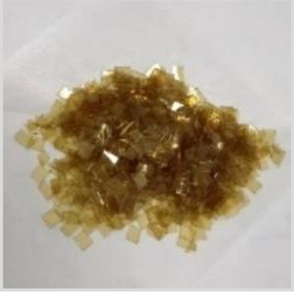	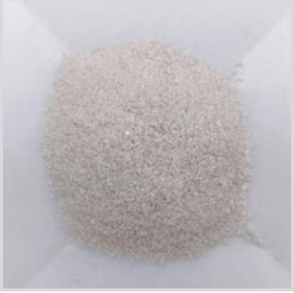	95.9 ± 1.4	95.1 ± 0.6

Black box	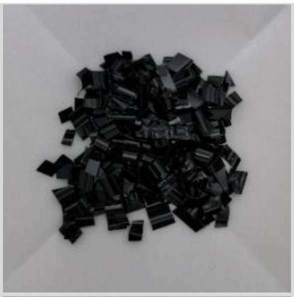	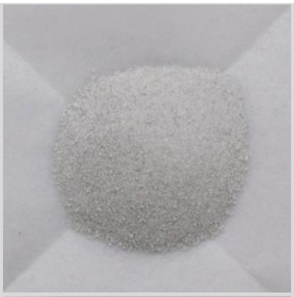	93.7 ± 1.4	93.3 ± 0.8
Black tray	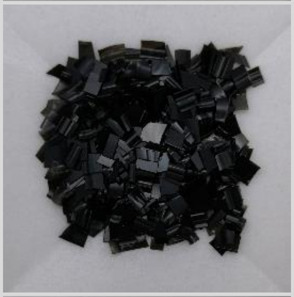	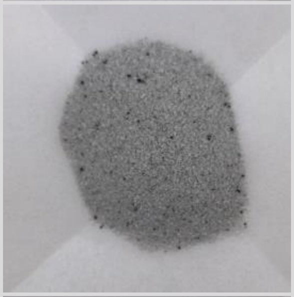	91.5 ± 1.8	91.0 ± 1.0
Transparent tray	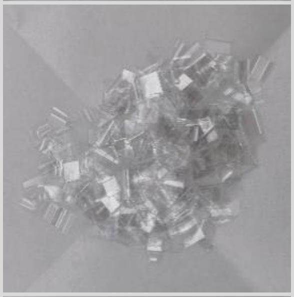	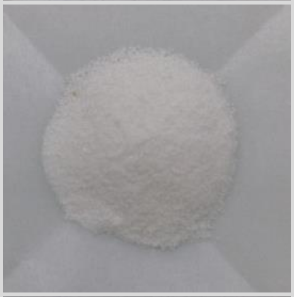	95.5 ± 0.8	92.5 ± 0.6
Transparent box	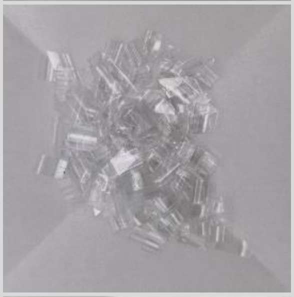	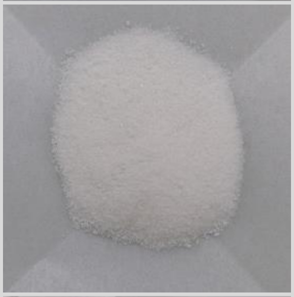	96.5 ± 0.8	95.4 ± 0.6
PET+PE	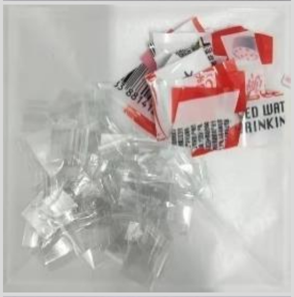	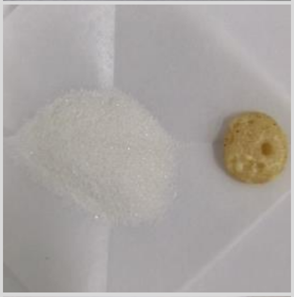	94.1 ± 1.4	93.6 ± 0.6
PET/PP	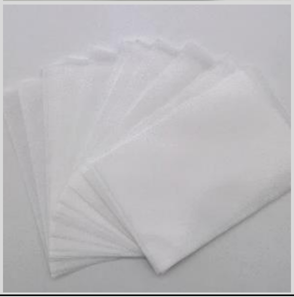	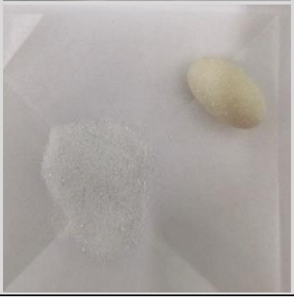	96.2 ± 1.4	95.6 ± 0.8

Reactions conditions: Waste plastic (60g), 300 mL acetic acid, 280 ^{o}C, 2 h.

Supplementary Table 8 Determine the content of 4-CBA and *p*-TOL in TPA by HPLC [4].

The source of TPA	4-CBA (mg/kg)	*p*-TOL (mg/kg)
TPA obtained from bottles.	n. d.	102.3
TPA obtained from textiles.	n. d.	127.3
TPA decolorized in an alkaline solution with activated carbon.	n. d.	118.5
TPA decolorized in N, N-dimethylacetamide with activated carbon.	n. d.	119.2
TPA decolorized in hot water (280°C) with activated carbon.	n. d.	108.6
The GB/T 32685-2016 standard [5]	≤25	≤150
The ASTM D7976-2020 International standard [6]	≤25	≤190

These decolorized TPA can be seen in Fig. 3 in main text. n. d. = not detected.

Supplementary Table 9 Acetolysis of other plastics.

	PETG	PEF	PEN	PBT
Polyester	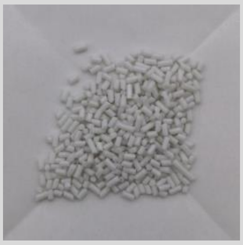	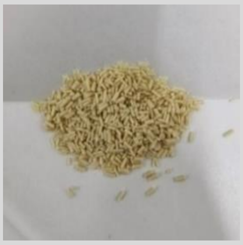	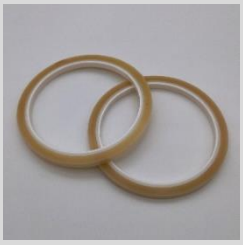	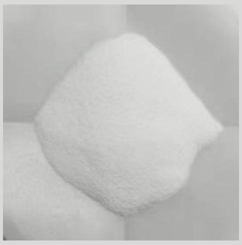
Structure	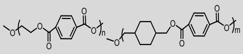	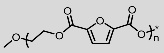	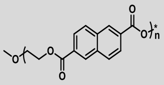	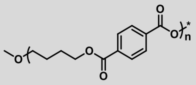
Product	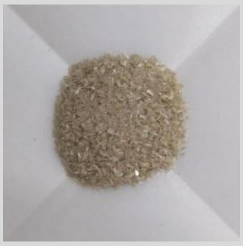	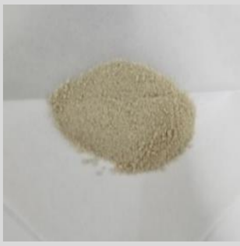	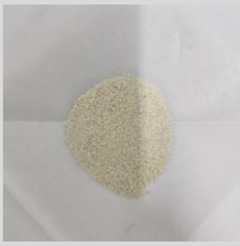	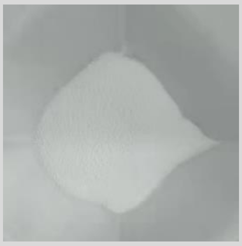
	TPA	2,5-FDCA	2,6-NDA	TPA
Yield/%	87.3 ± 1.8	89.3 ± 1.4	88.9 ± 2.4	92.3 ± 1.6

Reactions conditions: Waste plastic (5.0g), 25 mL acetic acid, 280 °C, 2 h.

Supplementary Table 10 Acetolysis of PET plastics containing blended impurities.

Samples	Products	Yield	NMR
Sample 1 (20% nylon 6 in PET)	6-acetylaminocaproic acid	18.80% (94.00%)	Fig. S31&S32
Sample 2 (20% polyacrylonitrile in PET)	acetamide	19.36% (96.80%)	Fig. S33&S34
Sample 3 (5 % nylon 66 in PET)	adipic acid	4.96 % (99.20%)	Fig. S35&S36
	diacetyl hexamethylene diamine	4.98 % (99.60%)	Fig. S37&S38
Sample 4 (6% spandex in PET)	acetanilide	5.68 % (94.67%)	Fig. S39&S40

Reactions conditions: Samples (5g), 20 mL acetic acid, 280 °C, 2 h.

Supplementary Figures

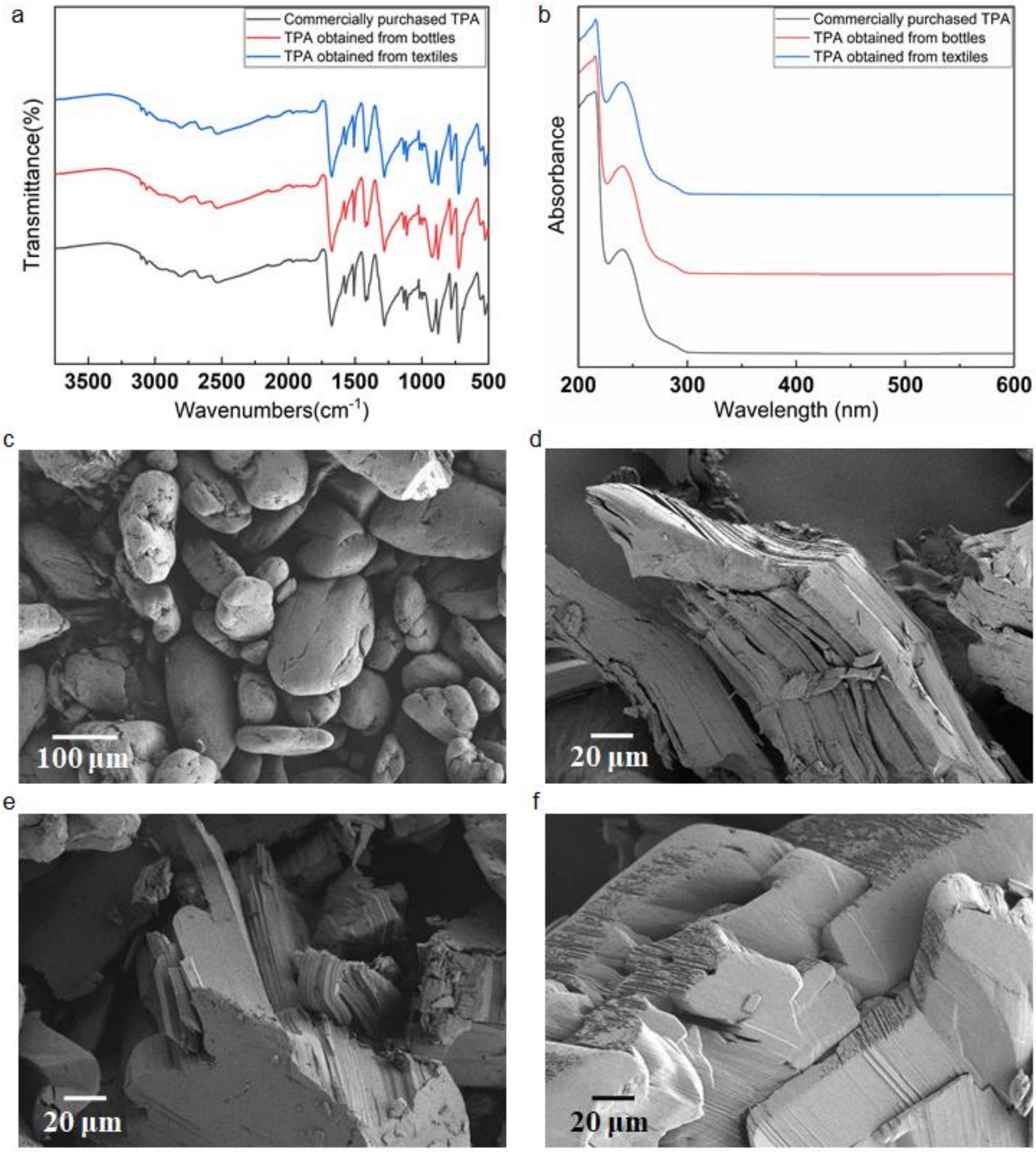

Supplementary Figure 8 Some spectral data of TPA obtained by acetolysis. a, FTIR spectra of TPA obtained from waste PET bottles and textiles **b,** UV-Vis spectra of TPA obtained from waste PET bottles and textiles. **c,** TPA powder purchased commercially (50 - 200 µm). **d,** TPA obtained by acetolysis of PET bottle fragments (500*X*). **e,** TPA obtained by acetolysis of PET textiles fragments (500*X*). **f,** TPA obtained by acetolysis of PET powder (500*X*). It can be seen from FTIR and UV-Vis spectroscopy that the TPA obtained by acetolysis has high purity, whether it comes from high-quality bottles or low-quality textiles.

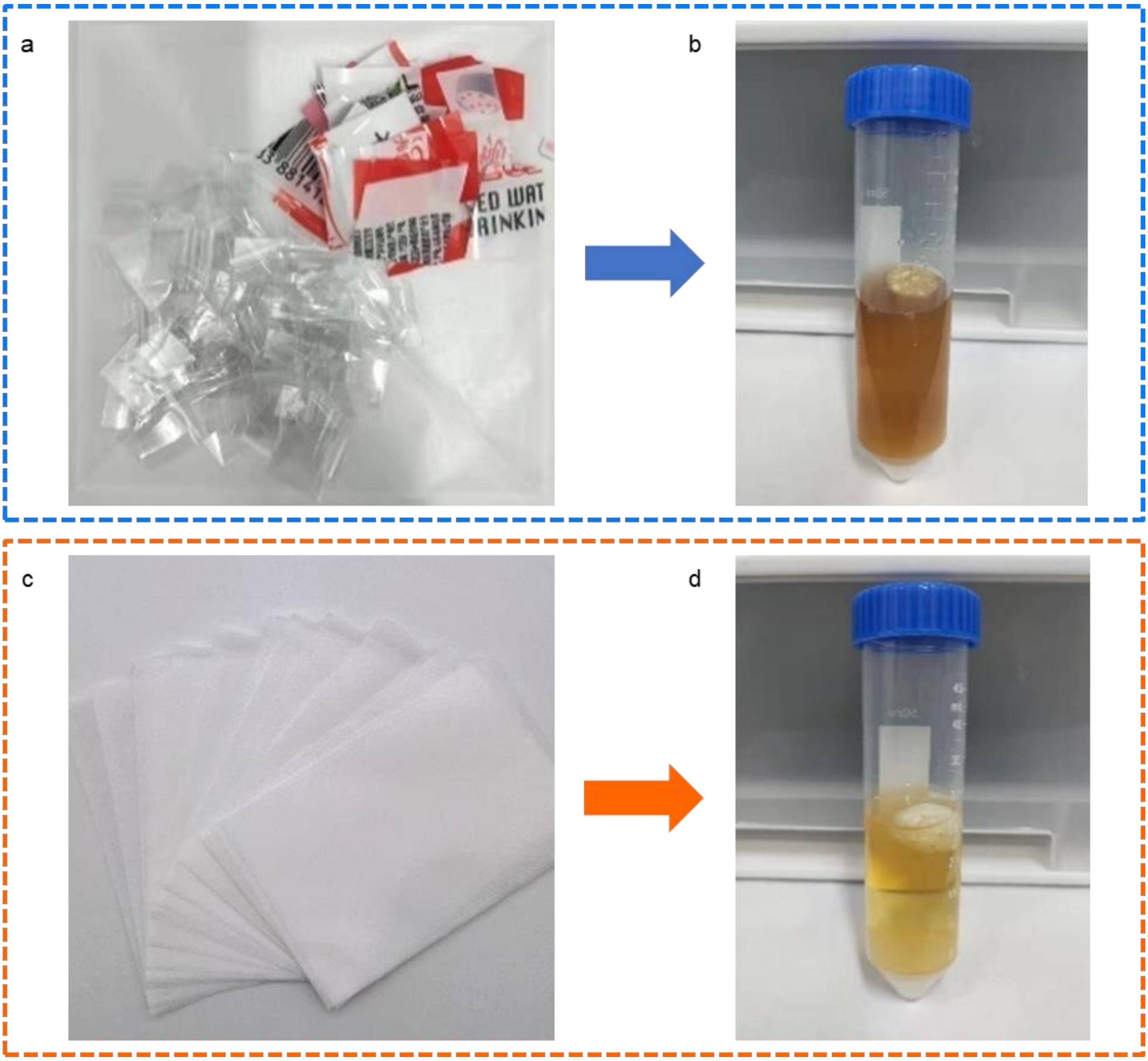

Supplementary Figure 9 Acetolysis of PET mixed with other plastics. a, Acetolysis of physically mixed flake PET with polyethylene (PE) labels. **b,** the reaction solution after acetolysis, PET is decomposed into TPA and settles, while PE melts, clumps, and floats on the solution. **c,** Acetolysis of PET-polypropylene (PP) composite films. **d,** the reaction solution after acetolysis, PET is decomposed into TPA and settles, while PP melts, clumps, and floats on the solution. This means that there is no need to separate the label for acetolysis of the PET bottles.

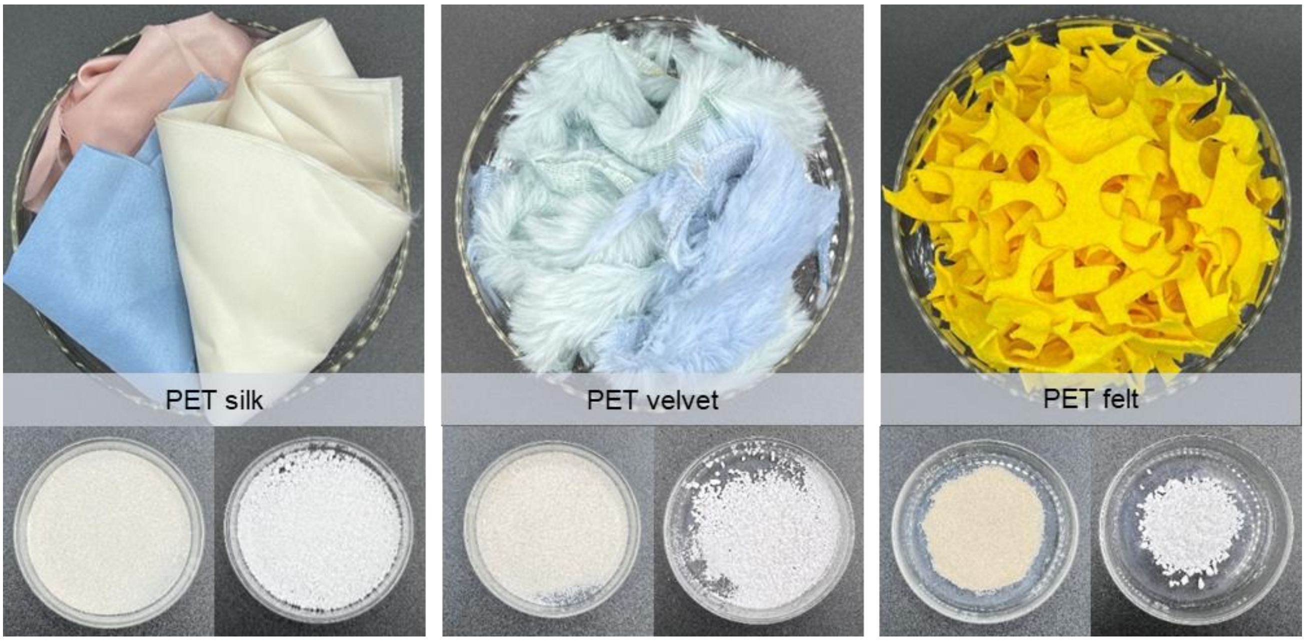

Supplementary Figure 10 Decolorized terephthalic acid obtained from different textiles yields greater than 90% and purity over 99.7% by decolored method 1. We have tried the acetolysis of all PET fibers available around us, and all of them can achieve good results.

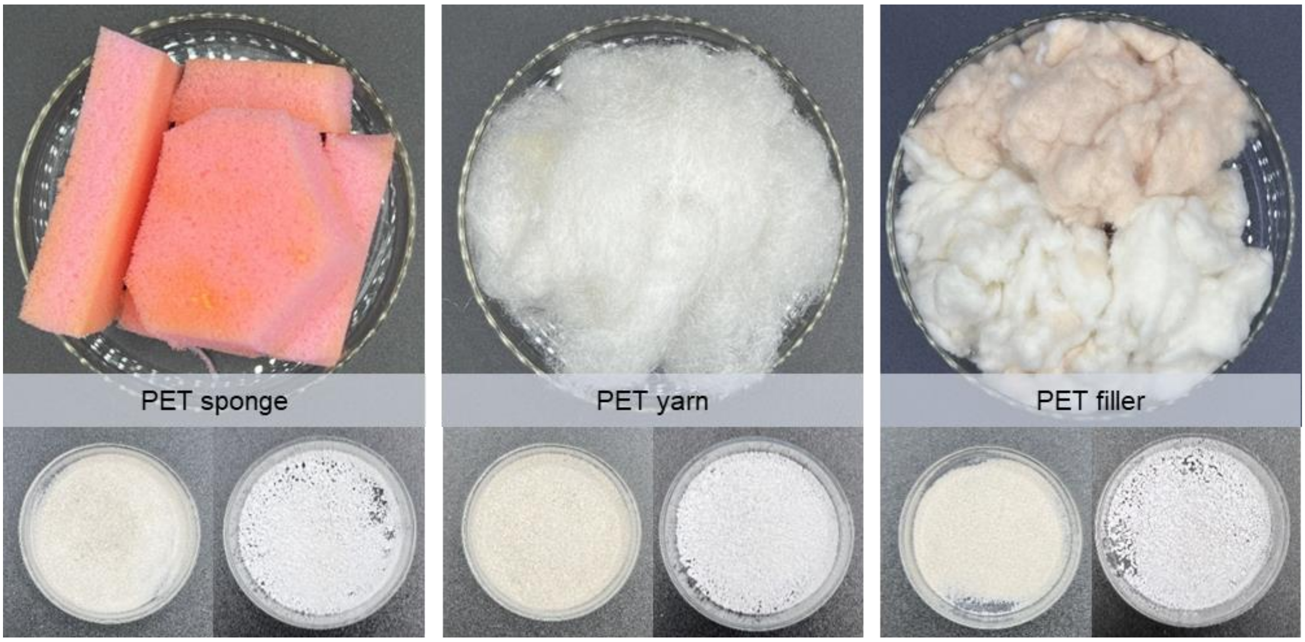

Supplementary Figure 11 Acetolysis of various PET fillers. Decolorized terephthalic acid obtained from different textiles yields greater than 90% and purity over 99.7% by decolored method 1. We have tried the acetolysis of all PET fibers available around us, and all of them can achieve good results.

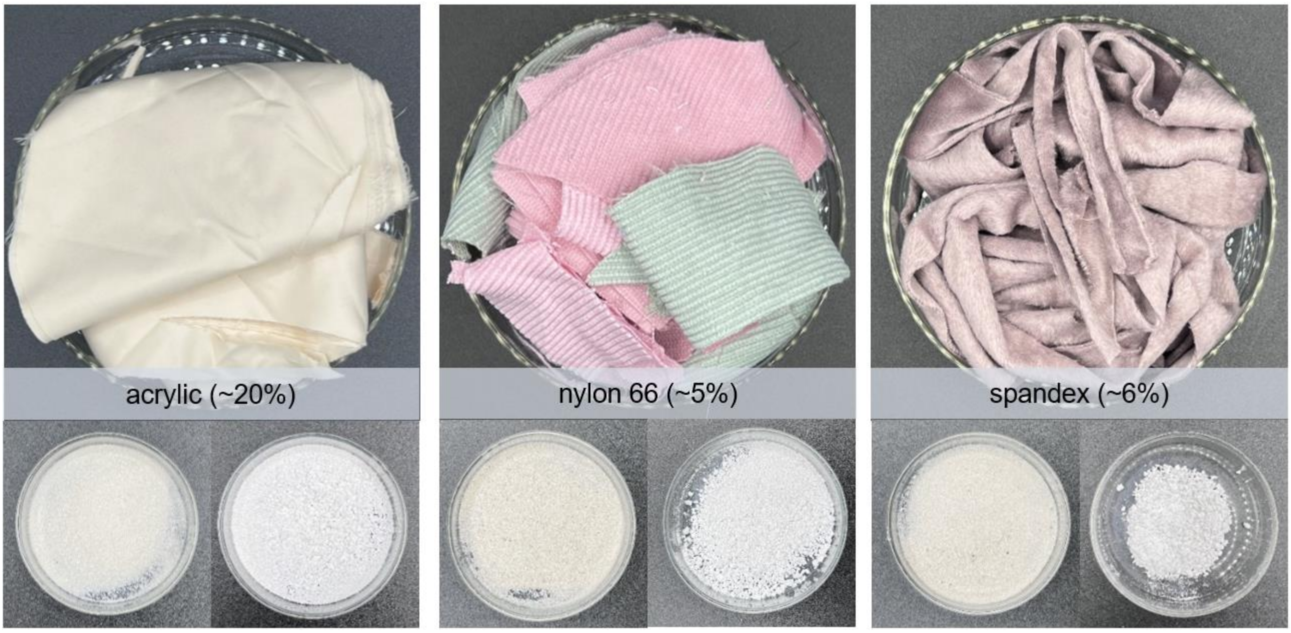

Supplementary Figure 12 Acetolysis of various textiles blended with PET. Decolorized terephthalic acid obtained from different textiles yields greater than 90% and purity over 99.7% by decolored method 1. Satisfactory results of acetolysis are due to the excellent dissolution/decomposition effect of acetic acid on most impurities.

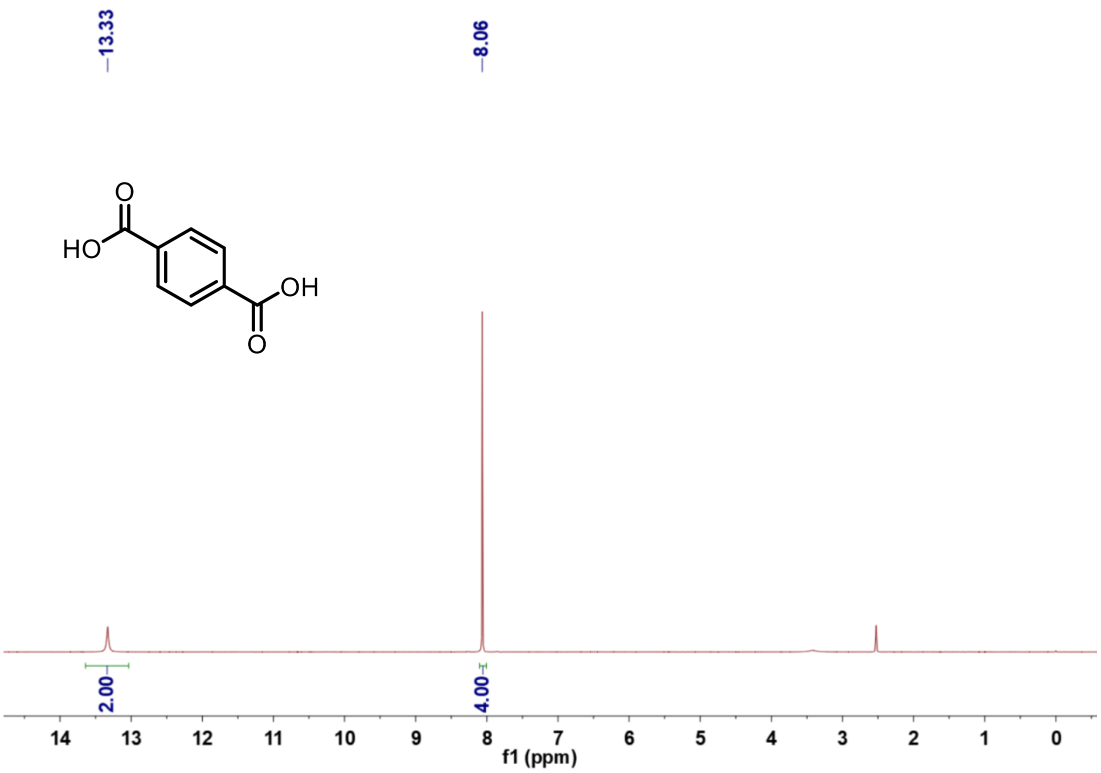

Supplementary Figure 13 ^{1}H-NMR spectrum of terephthalic acid in DMSO-d_6.

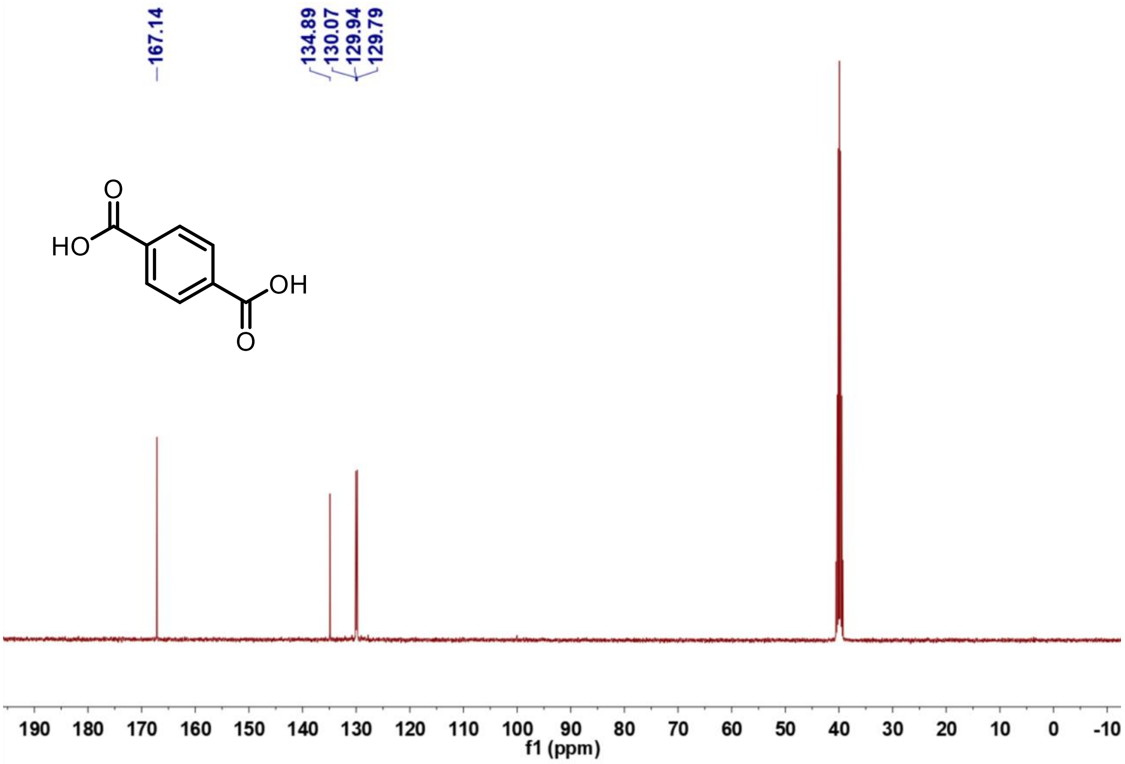

Supplementary Figure 14 ^{13}C-NMR spectrum of terephthalic acid in DMSO-d_6.

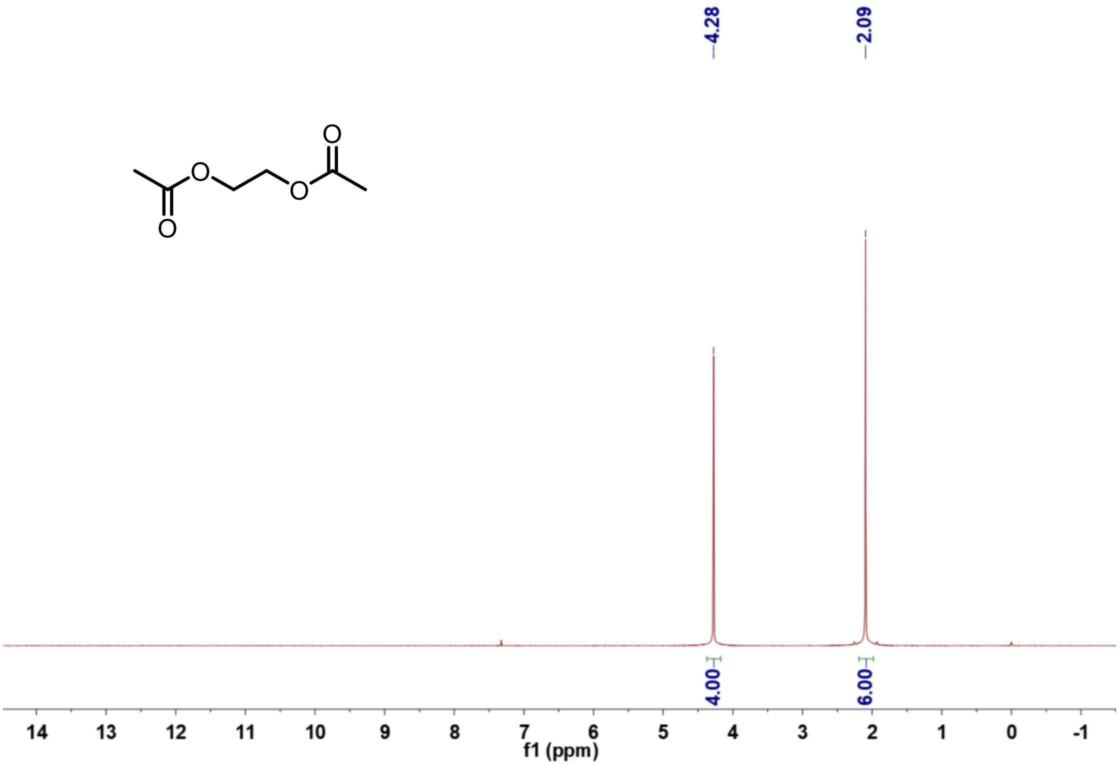

Supplementary Figure 15 ^{1}H-NMR spectrum of ethylene glycol diacetate in $CDCl_3$.

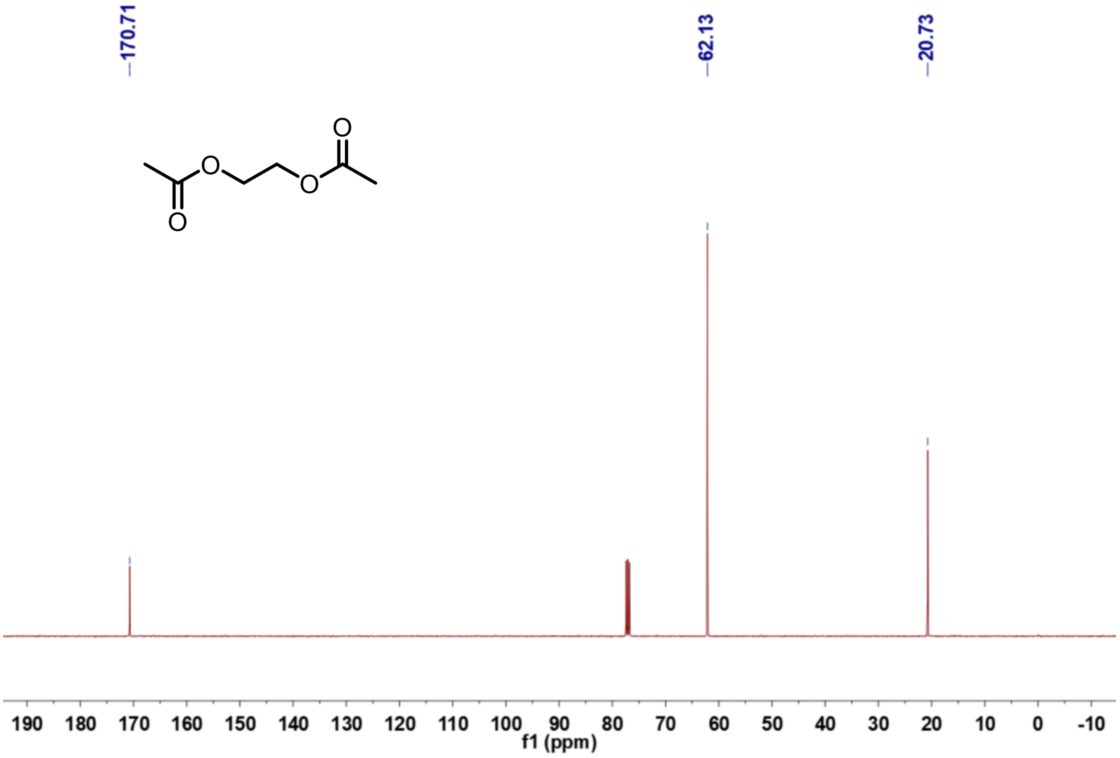

Supplementary Figure 16 ^{13}C-NMR spectrum of ethylene glycol diacetate in $CDCl_3$.

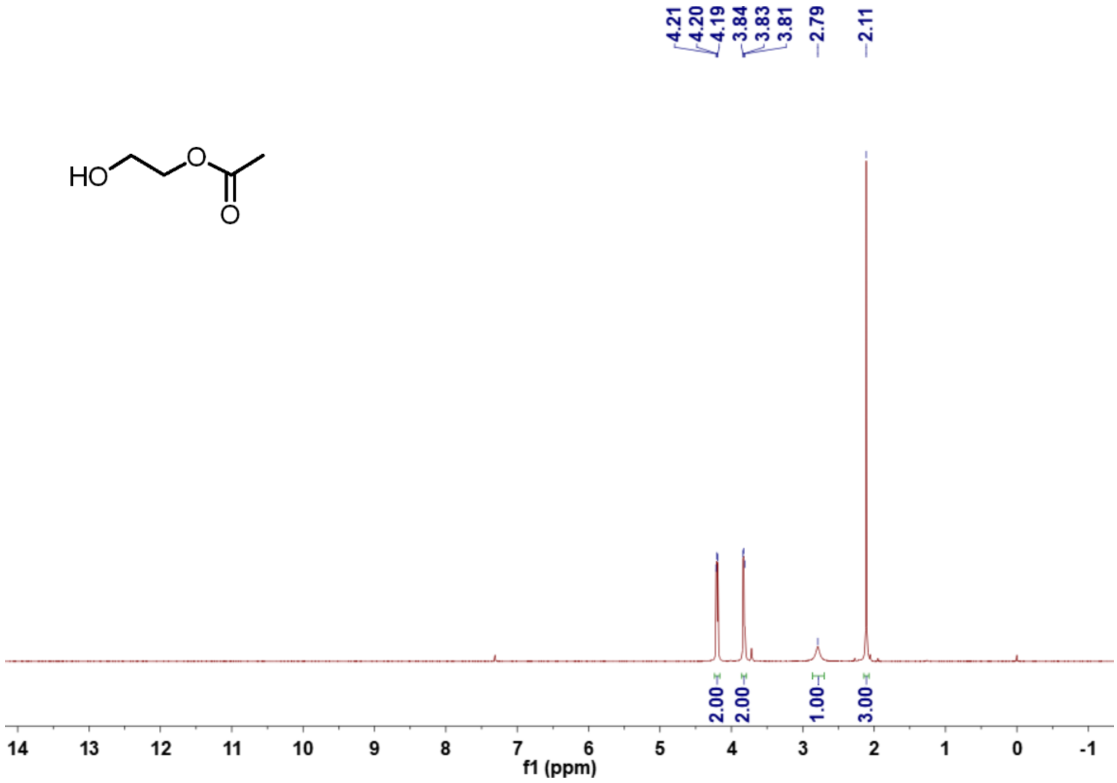

Supplementary Figure 17 ^{1}H-NMR spectrum of ethylene glycol monoacetate in $CDCl_3$.

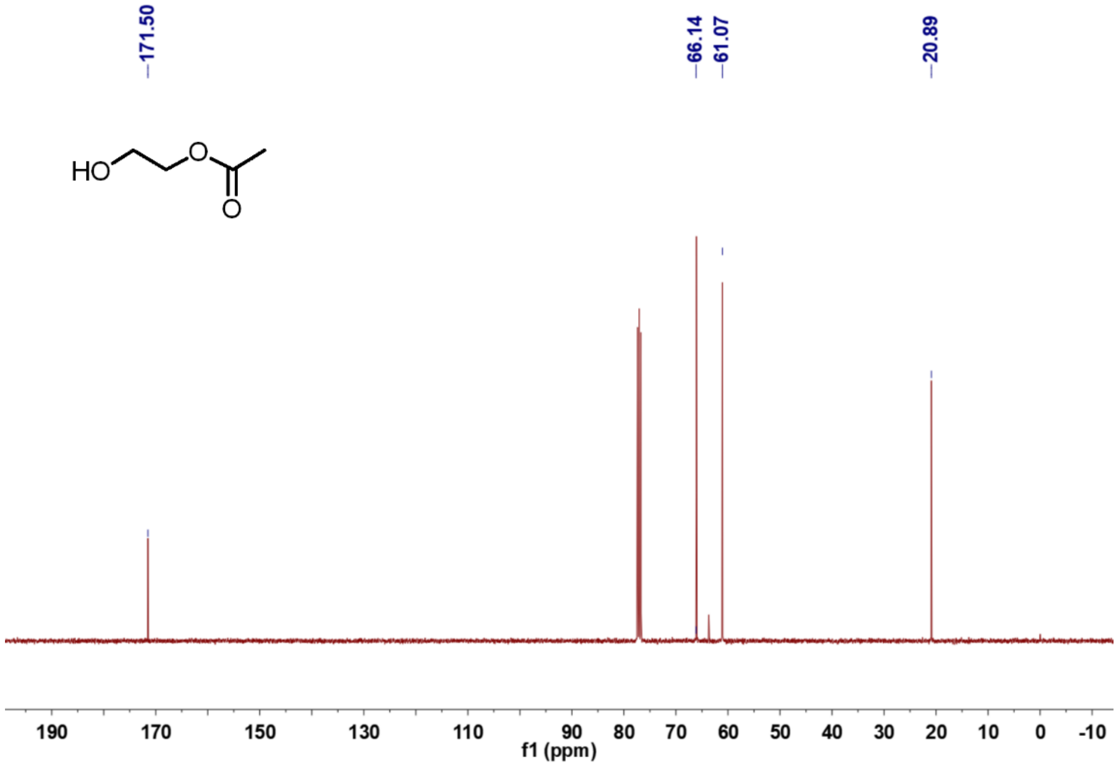

Supplementary Figure 18 ^{13}C-NMR spectrum of ethylene glycol monoacetate in $CDCl_3$.

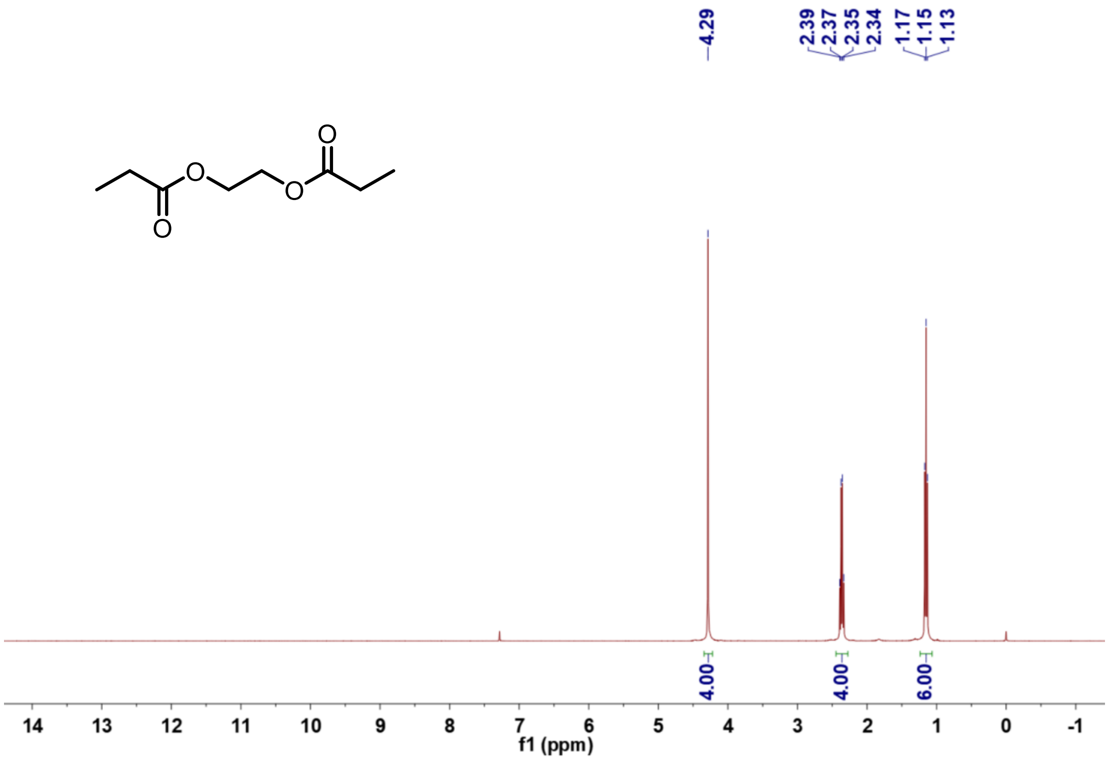

Supplementary Figure 19 ^{1}H-NMR spectrum of ethylene glycol dipropionate in $CDCl_3$.

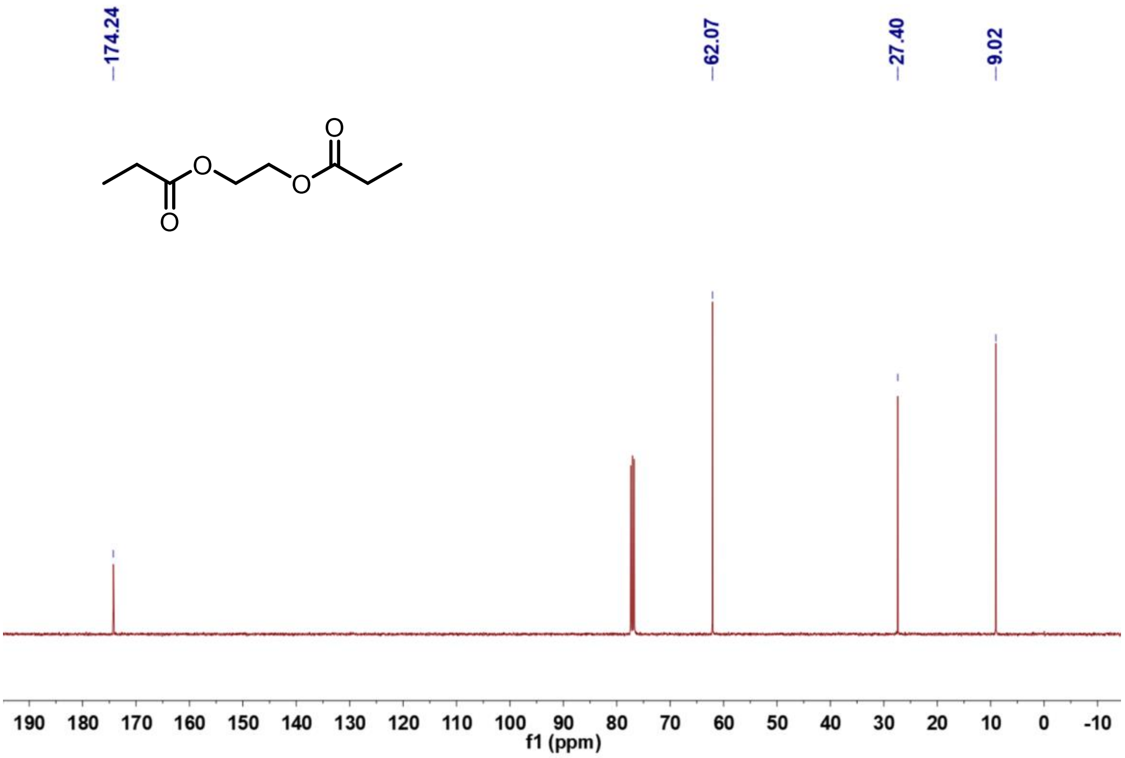

Supplementary Figure 20 ^{13}C-NMR spectrum of ethylene glycol dipropionate in $CDCl_3$.

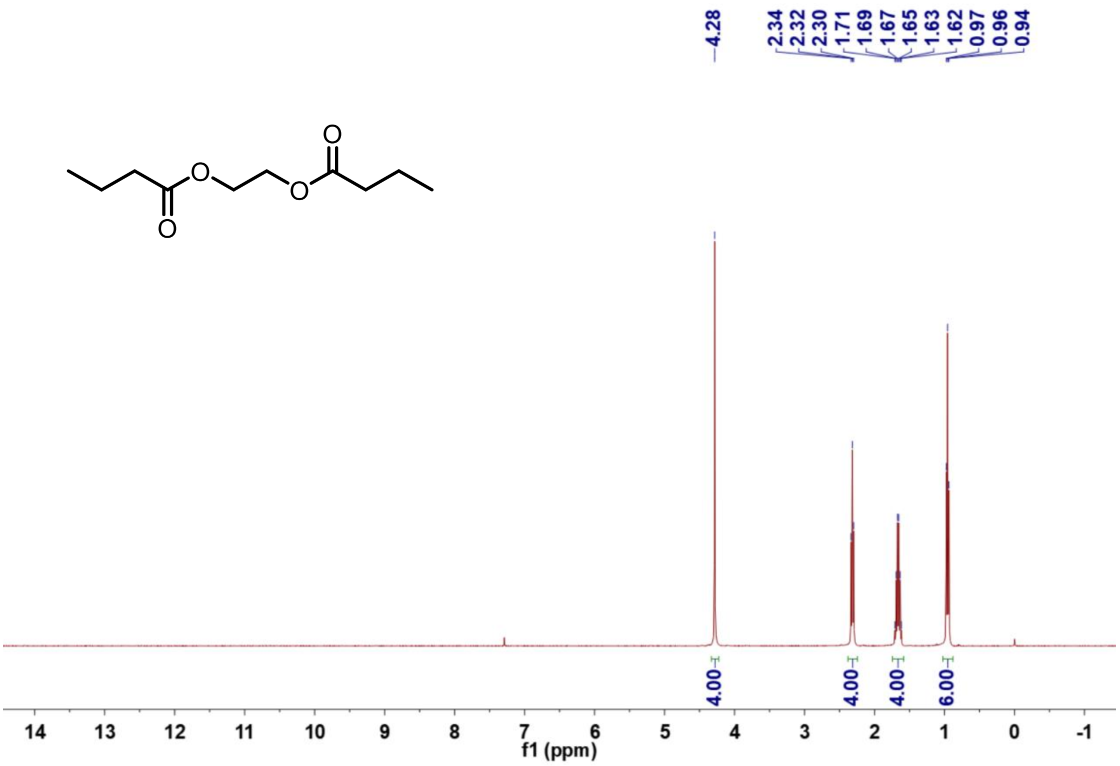

Supplementary Figure 21 ^{1}H-NMR spectrum of ethylene glycol di-*n*-butyrate in $CDCl_3$.

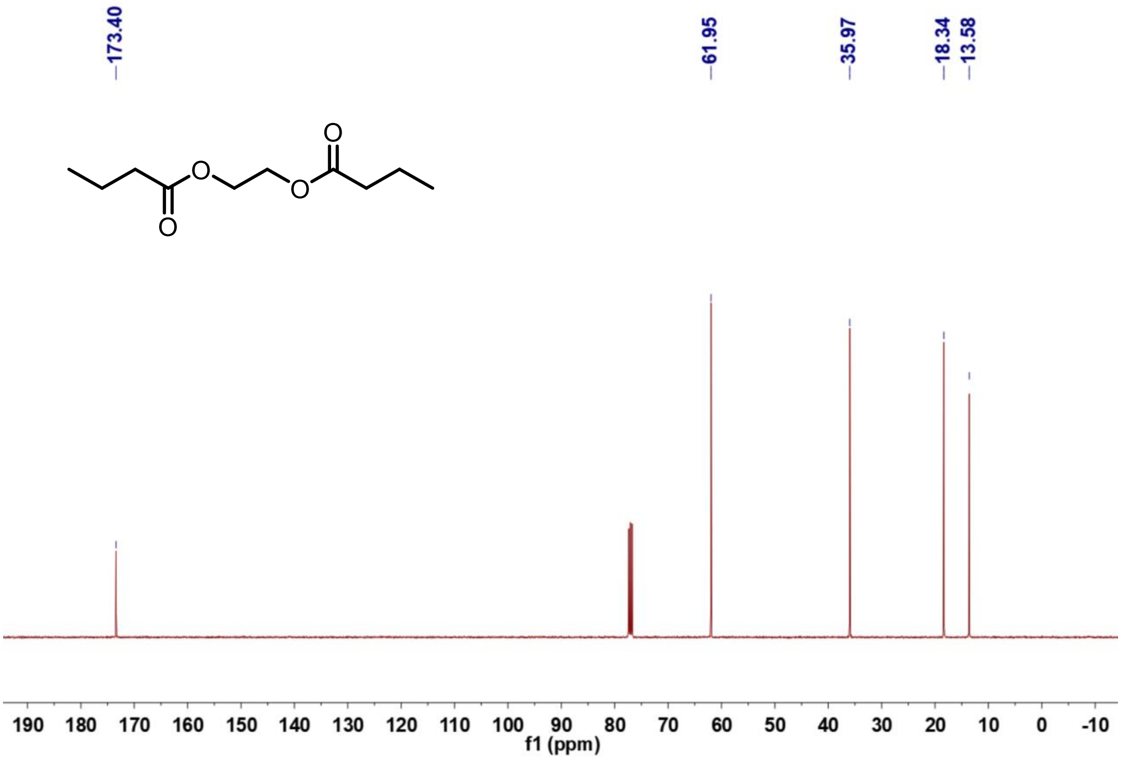

Supplementary Figure 22 ^{13}C-NMR spectrum of ethylene glycol di-*n*-butyrate in $CDCl_3$.

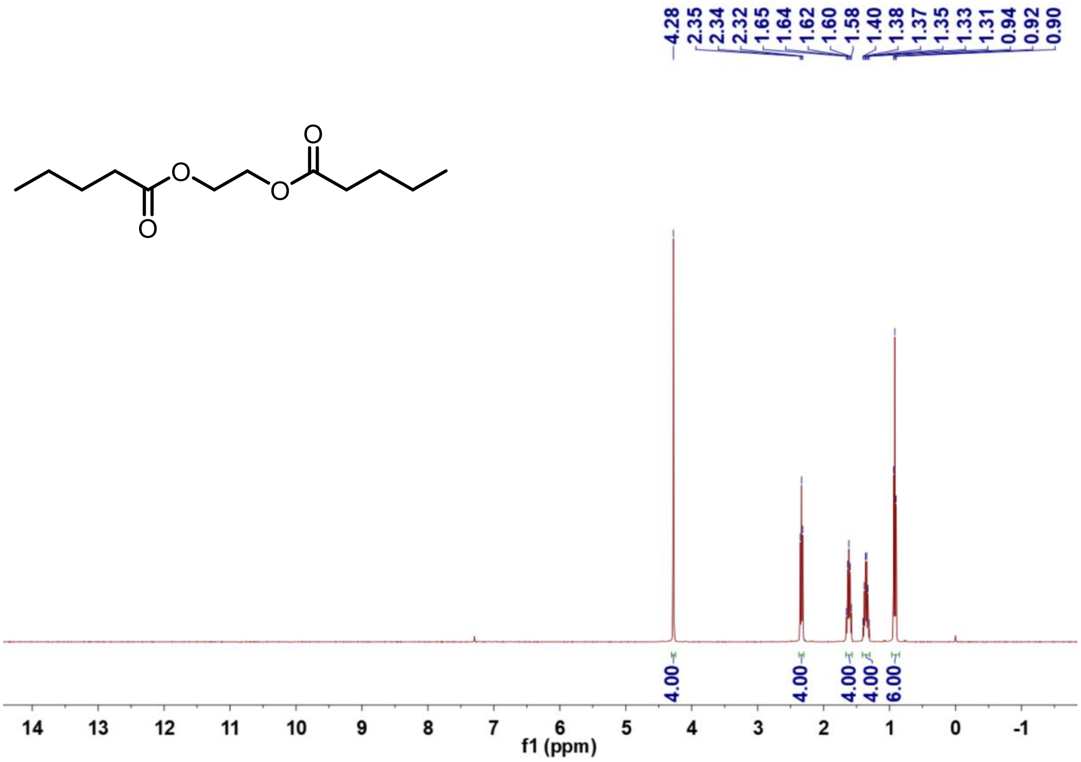

Supplementary Figure 23 ^{1}H-NMR spectrum of ethylene glycol valproate in $CDCl_3$.

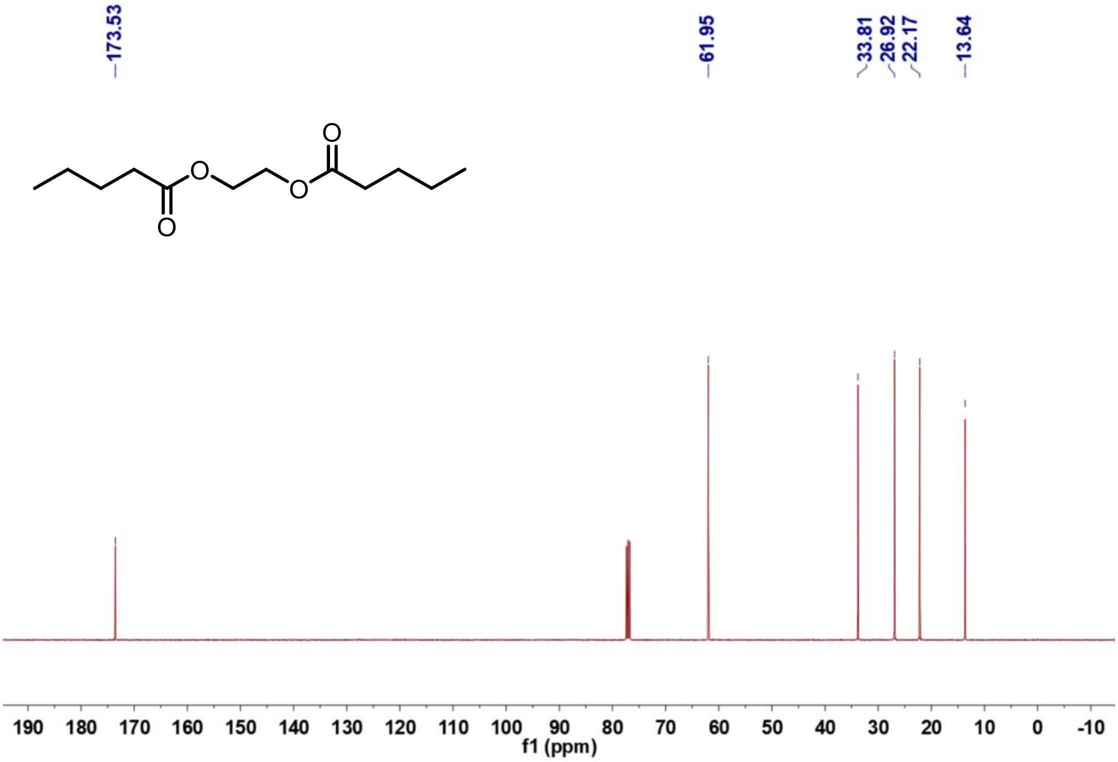

Supplementary Figure 24 ^{13}C-NMR spectrum of ethylene glycol valproate in $CDCl_3$.

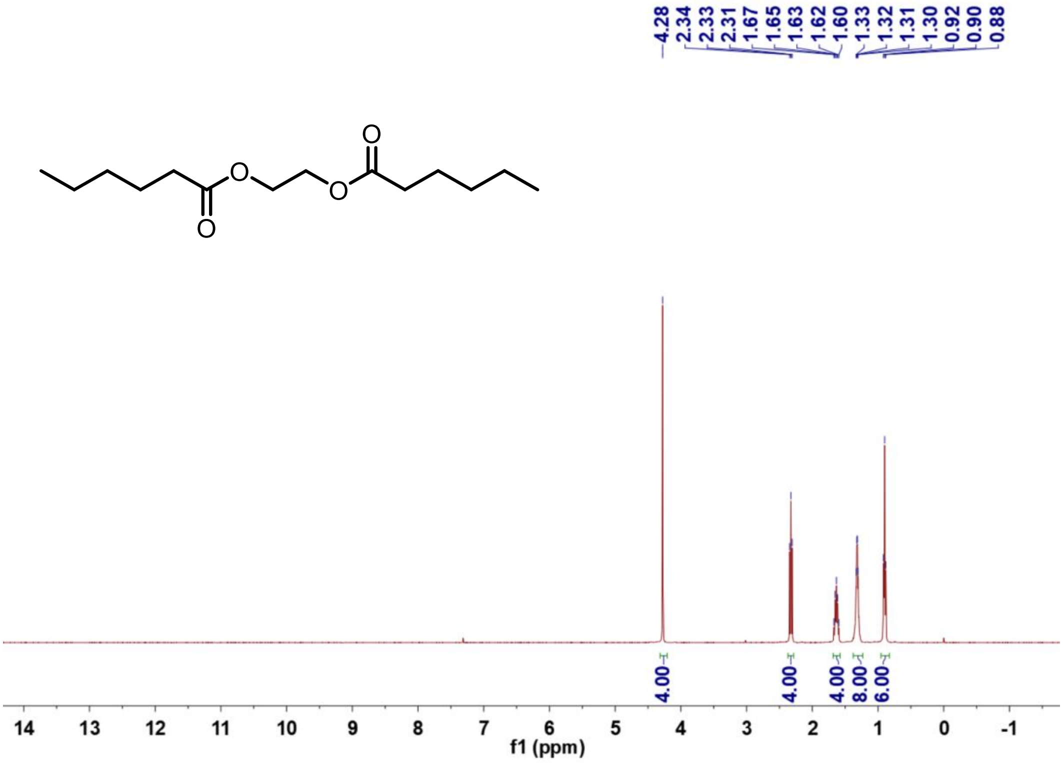

Supplementary Figure 25 ^{1}H-NMR spectrum of ethylene glycol di-*n*-hexanoate in $CDCl_3$.

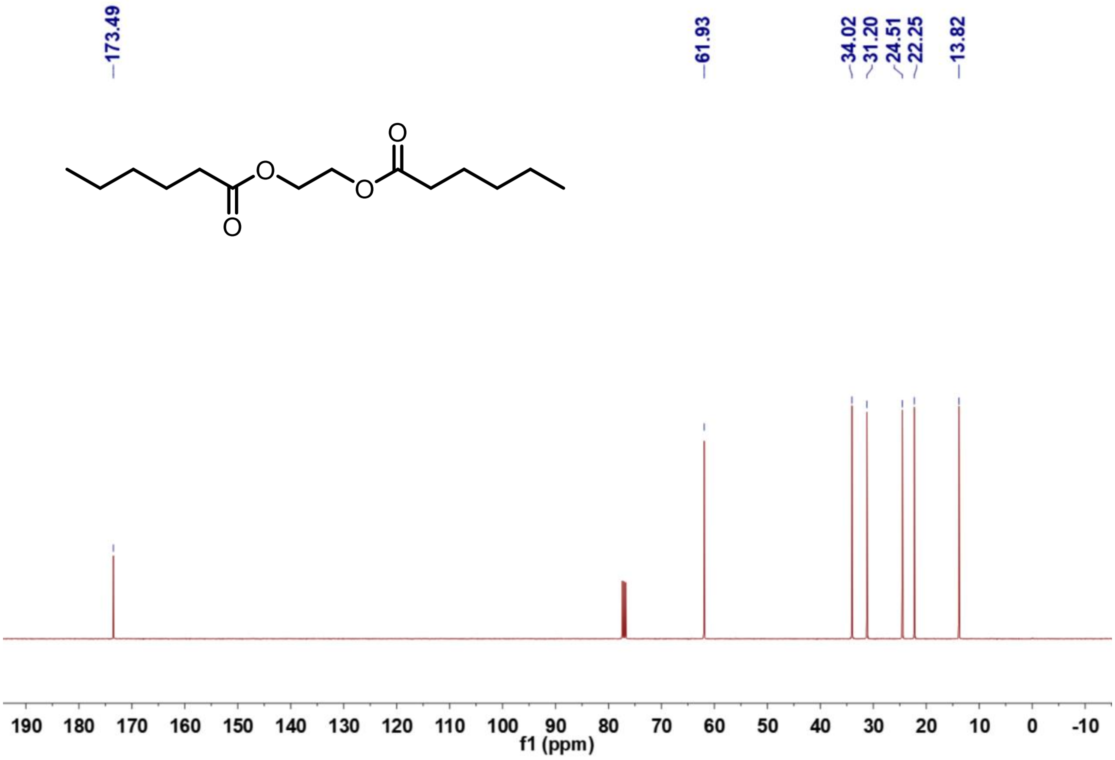

Supplementary Figure 26 ^{13}C-NMR spectrum of ethylene glycol di-*n*-hexanoate in $CDCl_3$.

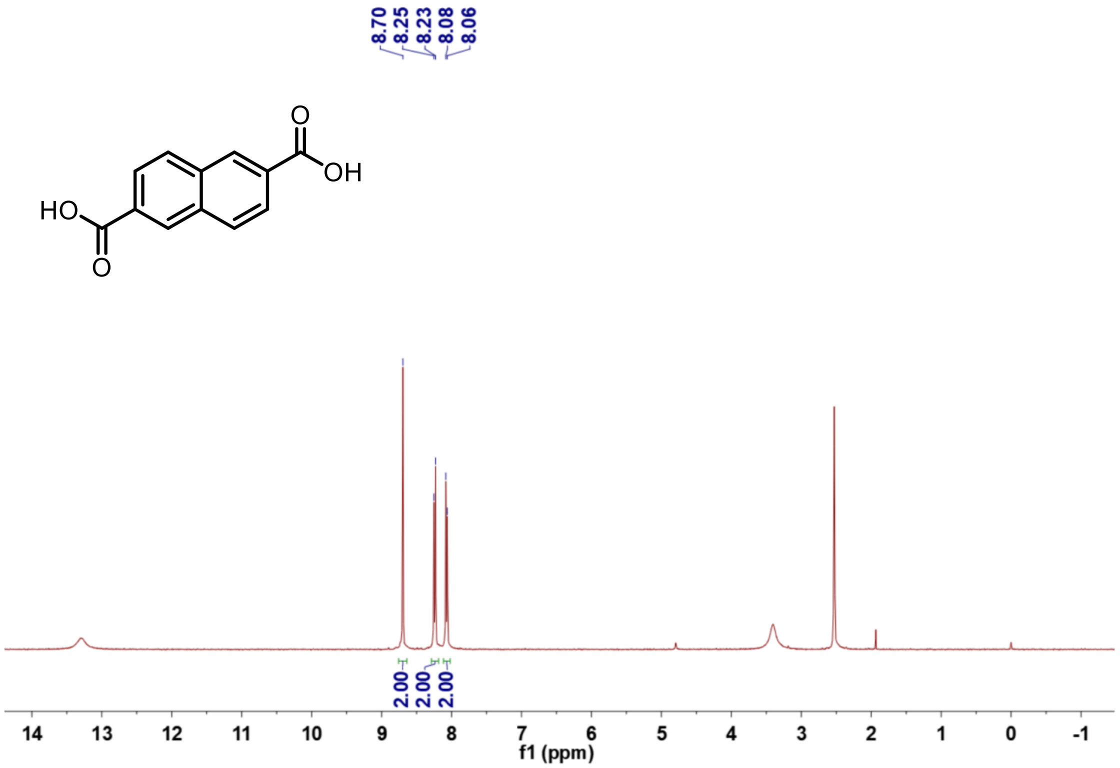

Supplementary Figure 27 ^{1}H-NMR spectrum of 2,6-naphthalene dicarboxylic acid in DMSO-d_6.

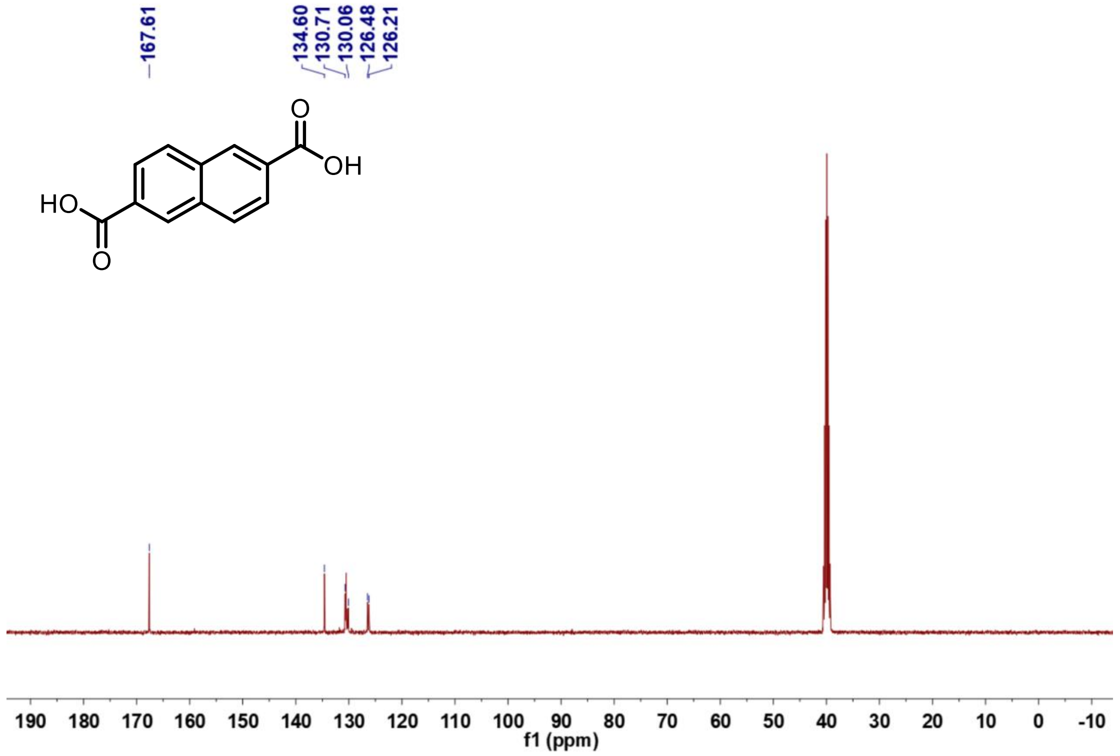

Supplementary Figure 28 ^{13}C-NMR spectrum of 2,6-naphthalene dicarboxylic acid in DMSO-d_6.

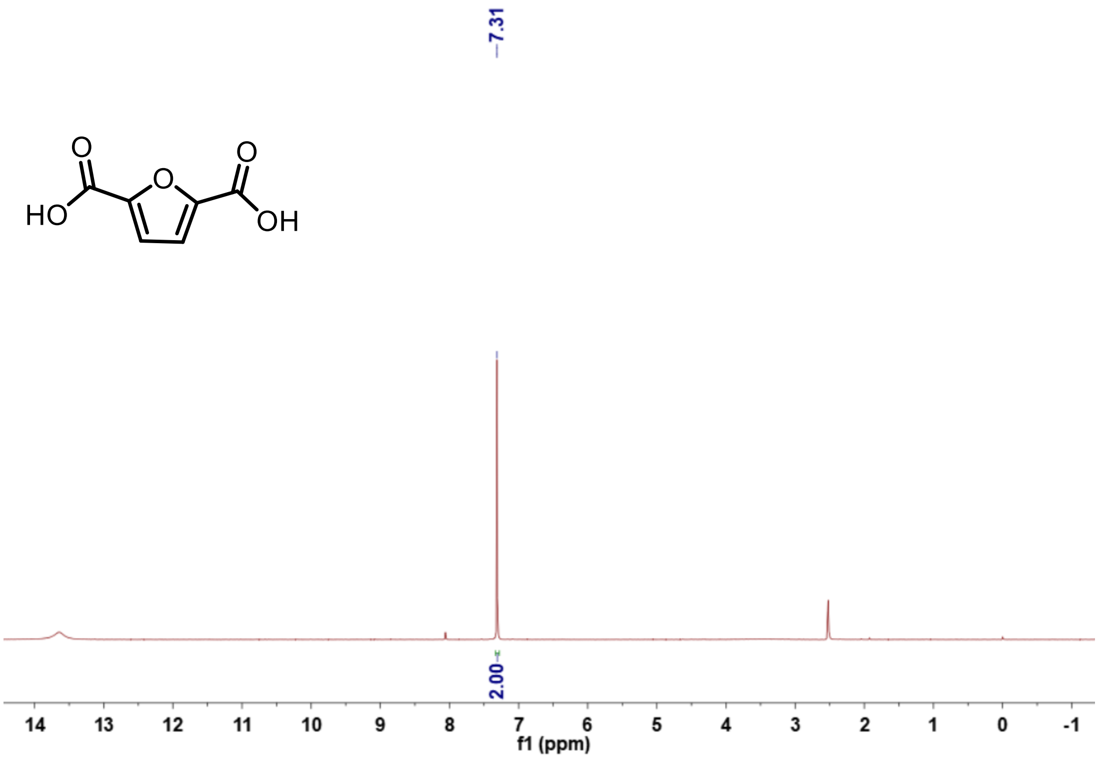

Supplementary Figure 29 ^{1}H-NMR spectrum of 2,5-furan dicarboxylic acid in DMSO-d_6.

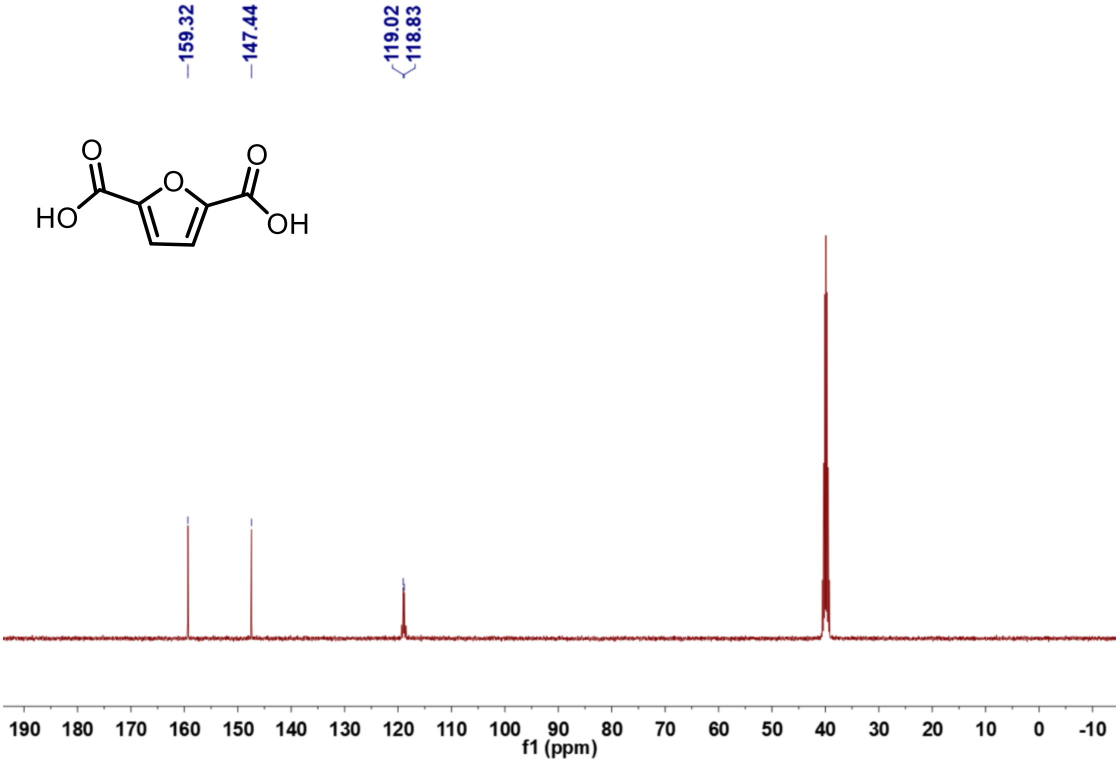

Supplementary Figure 30 ^{13}C-NMR spectrum of 2,5-furan dicarboxylic acid in DMSO-d_6.

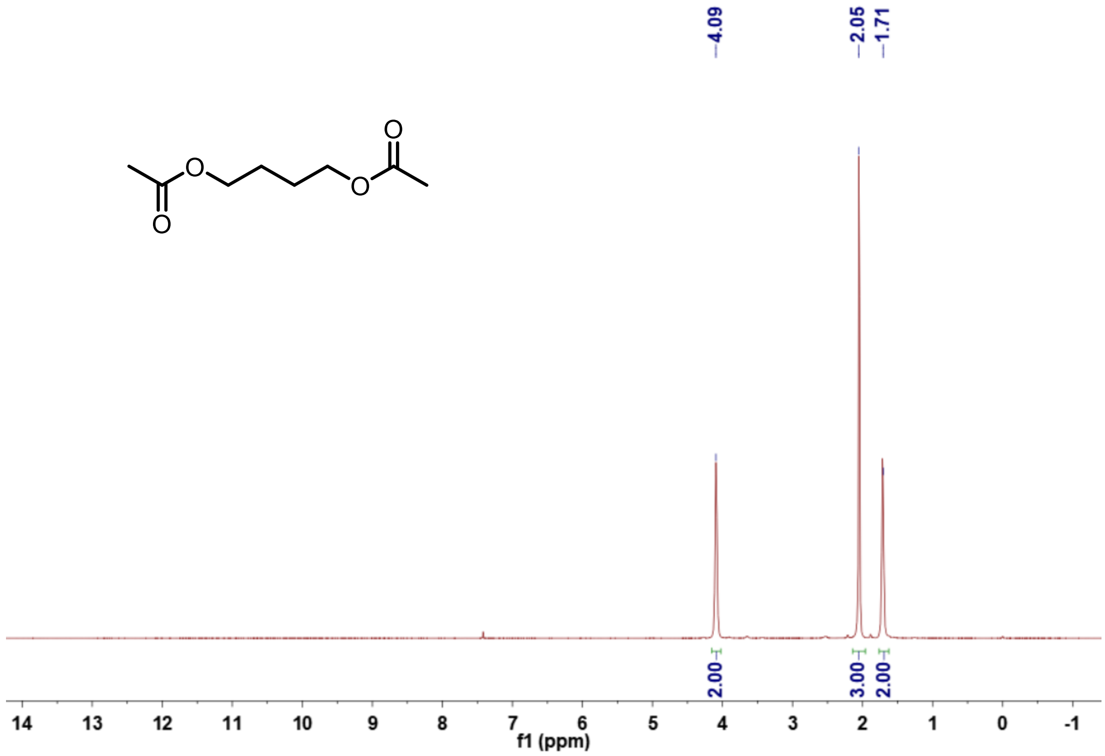

Supplementary Figure 31 ^{1}H-NMR spectrum of 1,4-diacetoxybutane in $CDCl_3$.

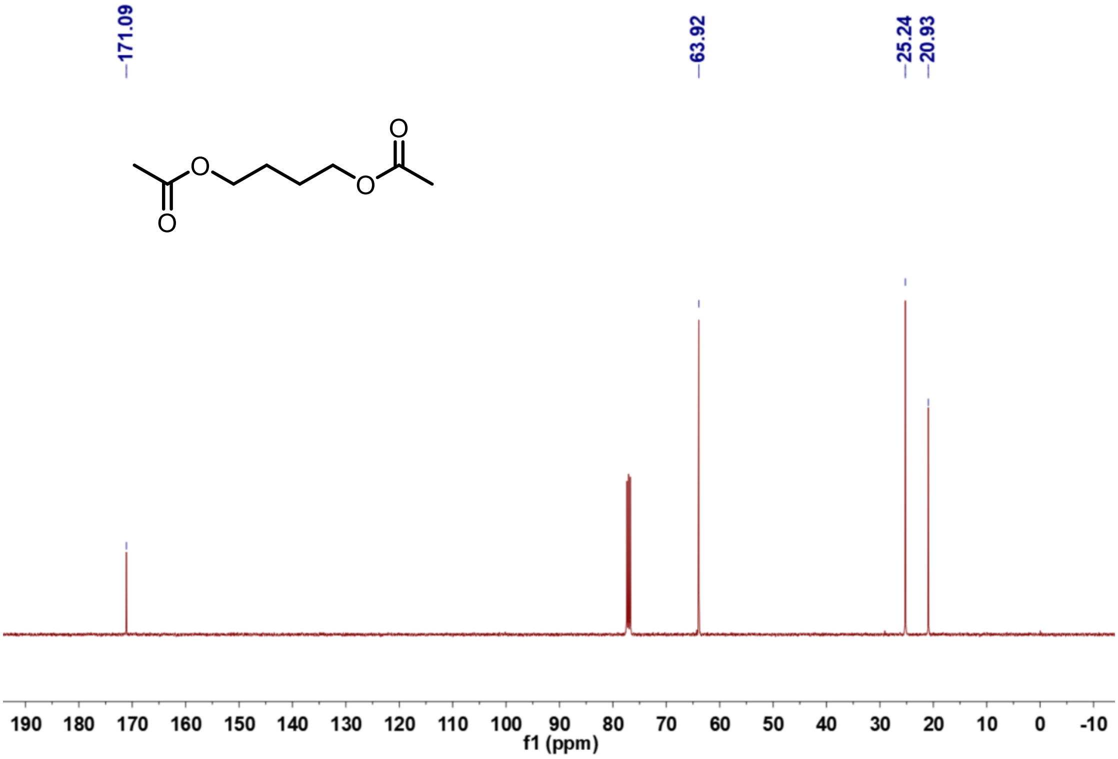

Supplementary Figure 32 ^{13}C-NMR spectrum of 1,4-diacetoxybutane in $CDCl_3$.

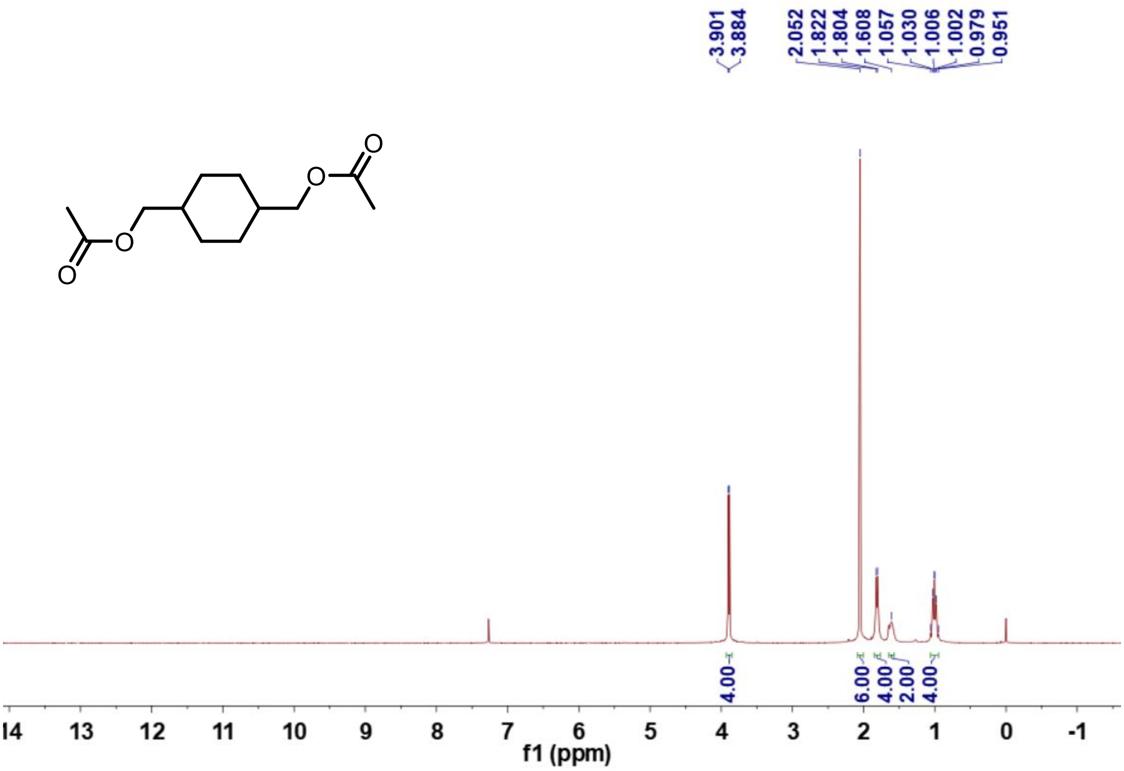

Supplementary Figure 33 ^{1}H-NMR spectrum of cyclohexane dimethyl diacetate in $CDCl_3$.

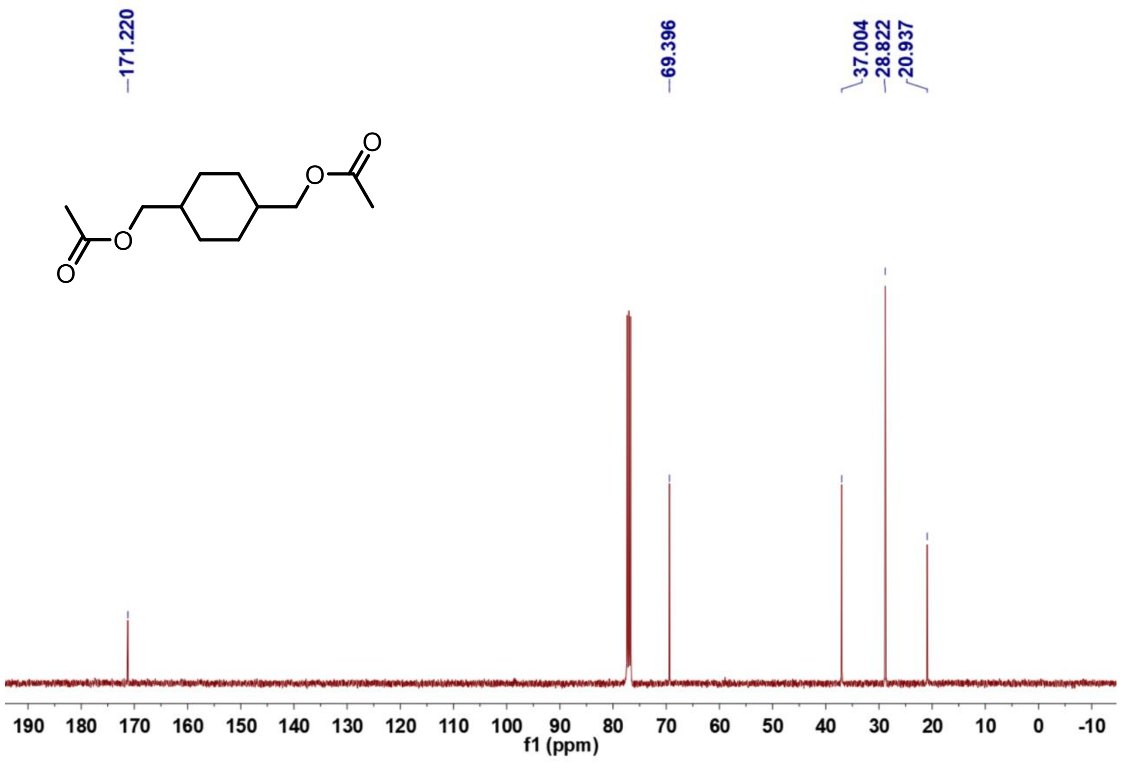

Supplementary Figure 34 ^{13}C-NMR spectrum of cyclohexane dimethyl diacetate in $CDCl_3$.

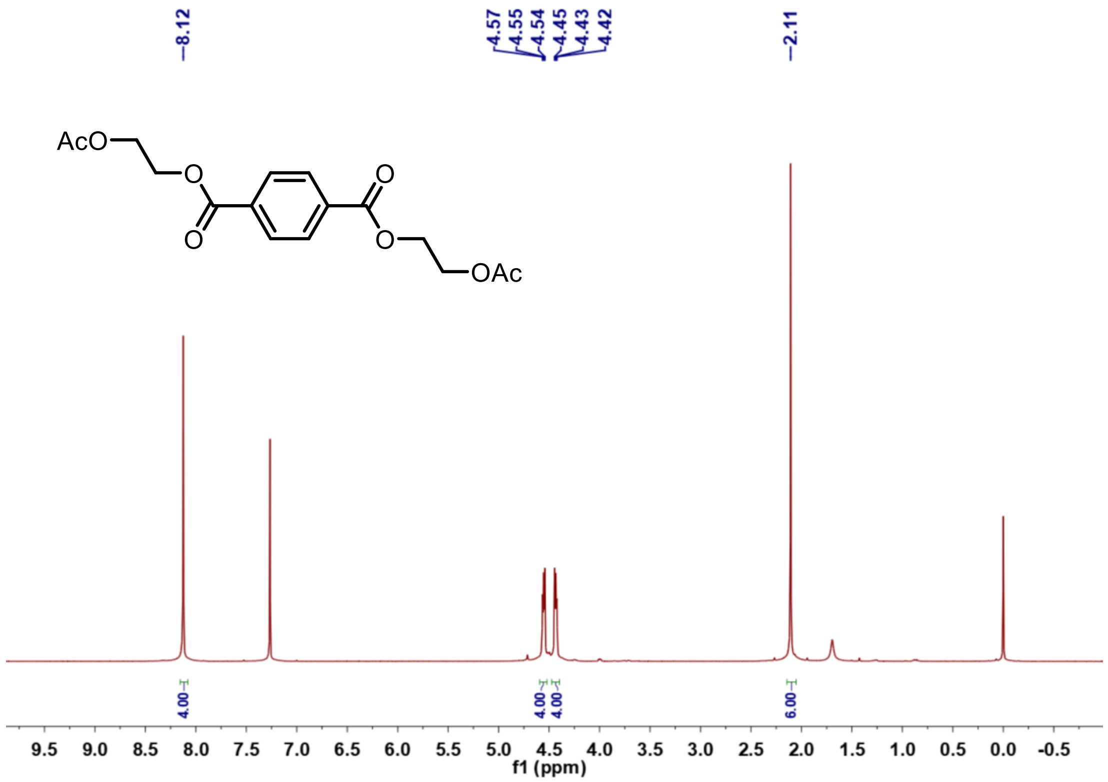

Supplementary Figure 35 ^{1}H-NMR spectrum of bis(2-acetoxyethyl) terephthalate in $CDCl_3$.

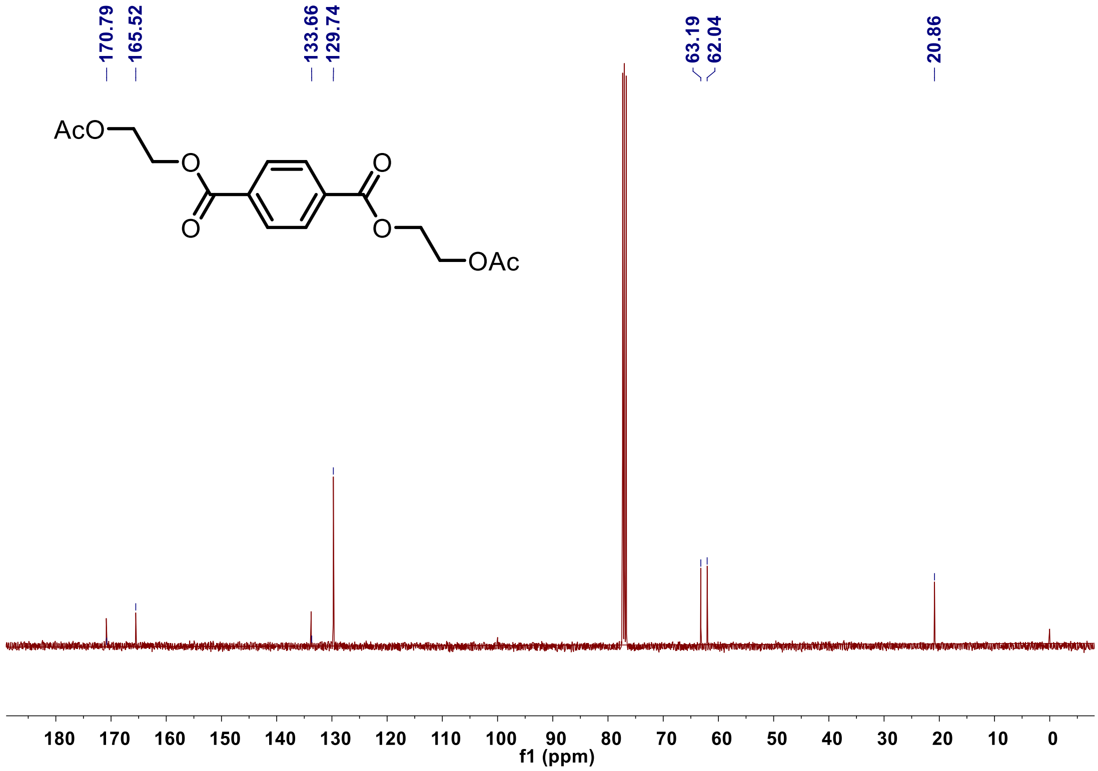

Supplementary Figure 36 ^{13}C-NMR spectrum of bis(2-acetoxyethyl) terephthalate in $CDCl_3$.

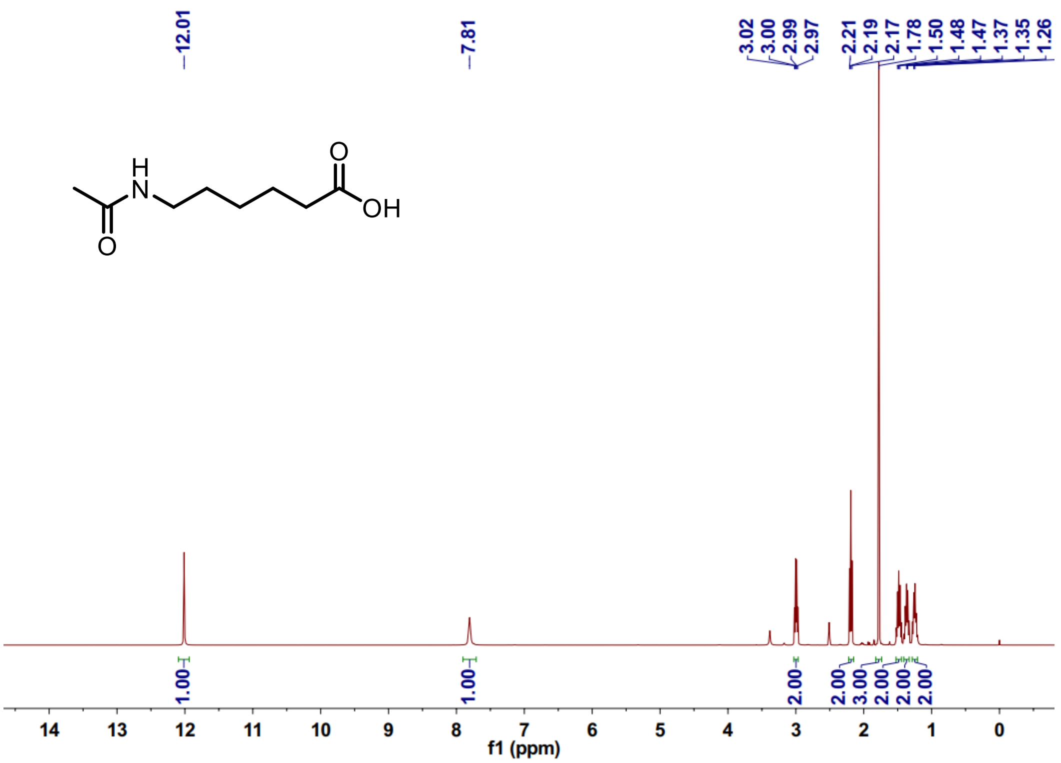

Supplementary Figure 37 ^{1}H-NMR spectrum of 6-acetylaminocaproic acid in DMSO.

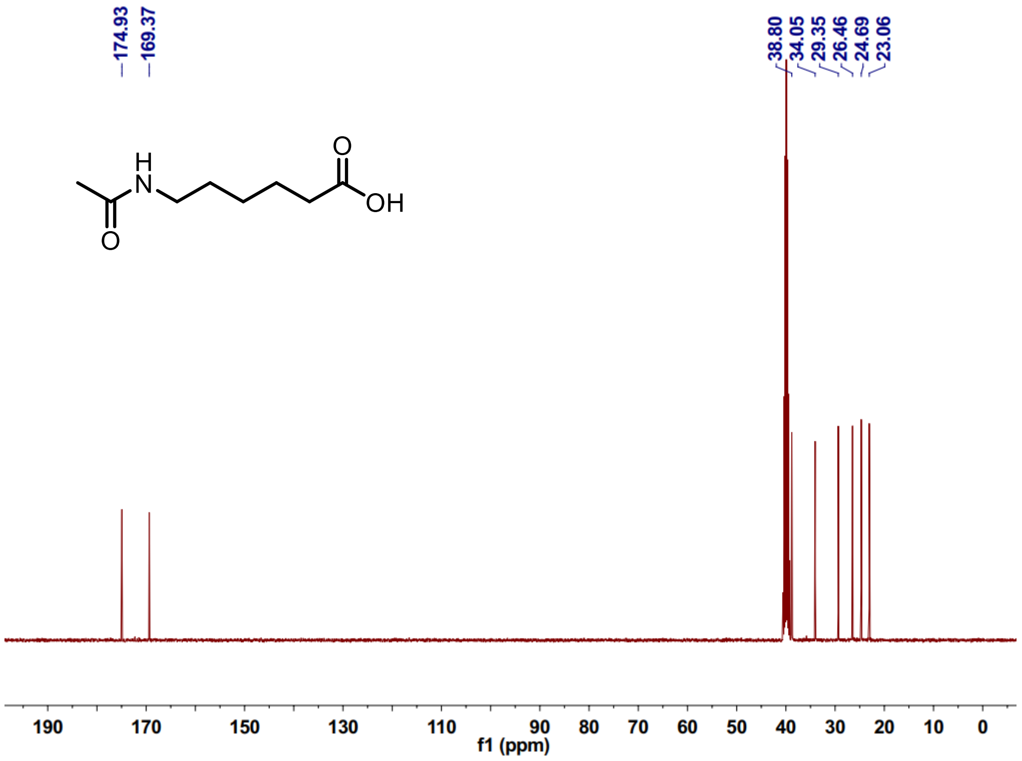

Supplementary Figure 38 ^{13}C-NMR spectrum of 6-acetylaminocaproic acid in DMSO.

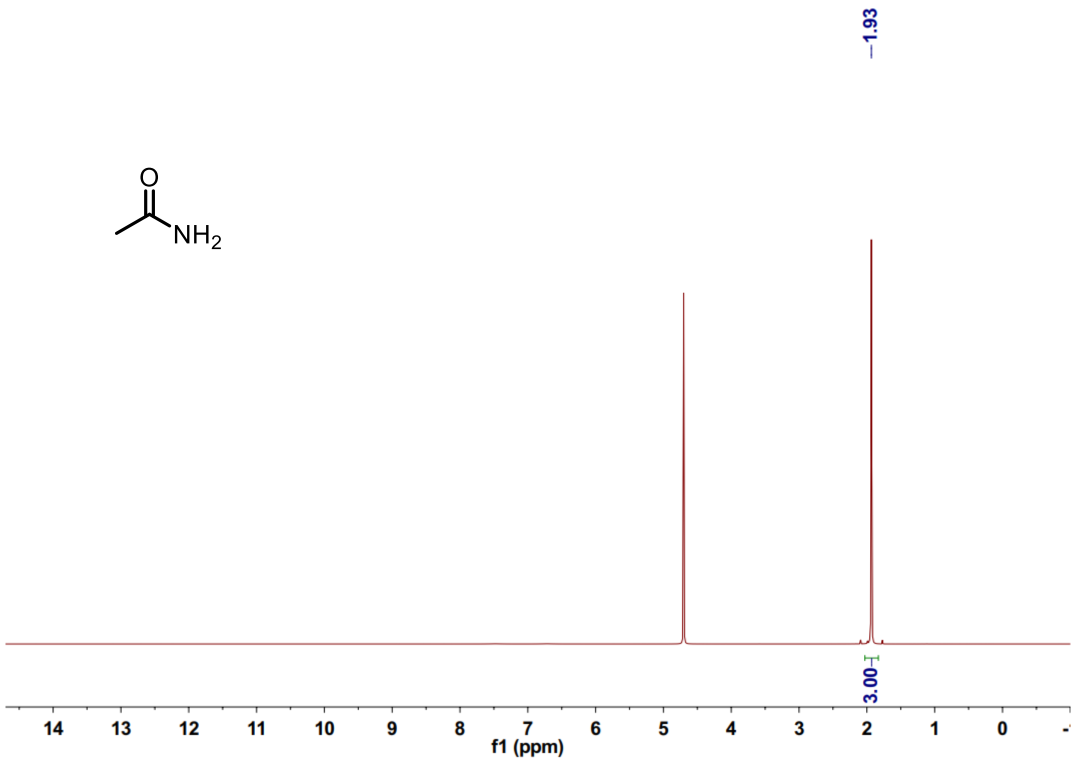

Supplementary Figure 39 ^{1}H-NMR spectrum of acetamide in D_2O.

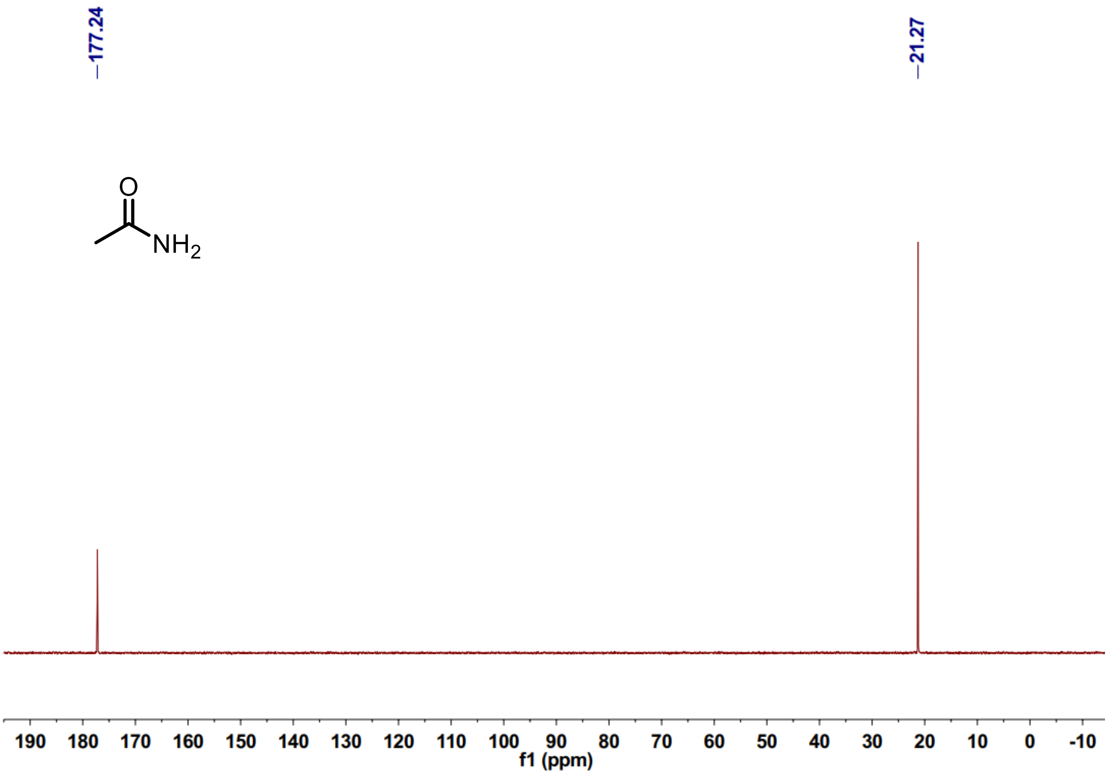

Supplementary Figure 40 ^{13}C-NMR spectrum of acetamide in D_2O.

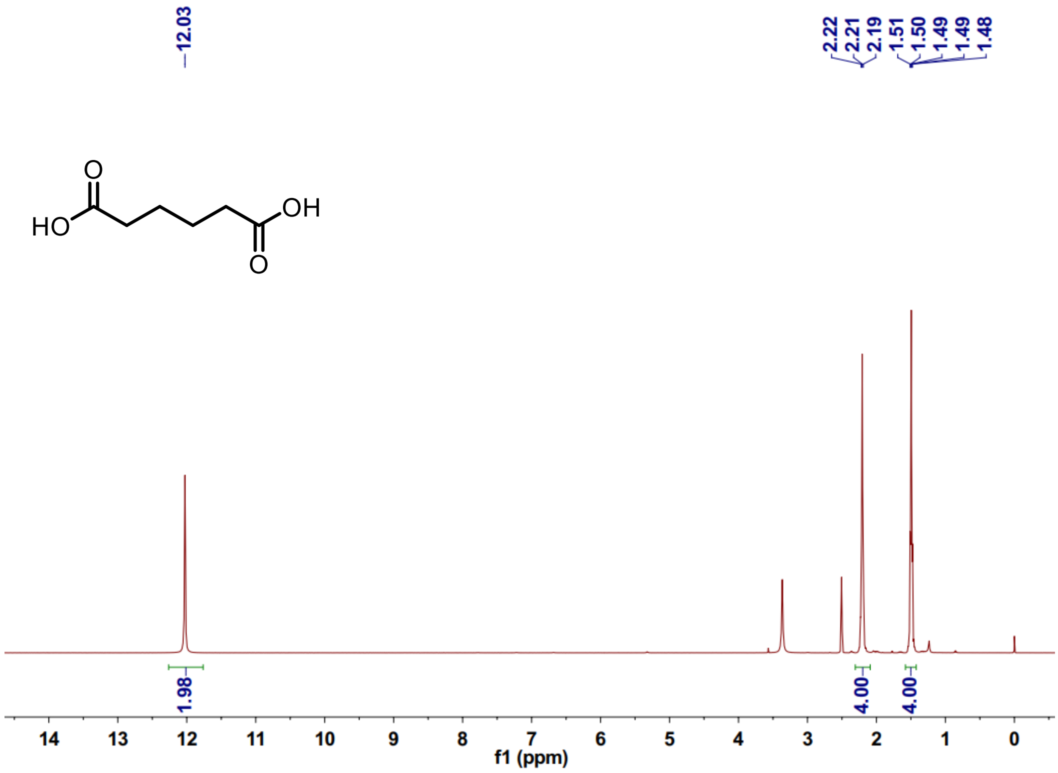

Supplementary Figure 41 ^{1}H-NMR spectrum of adipic acid in DMSO.

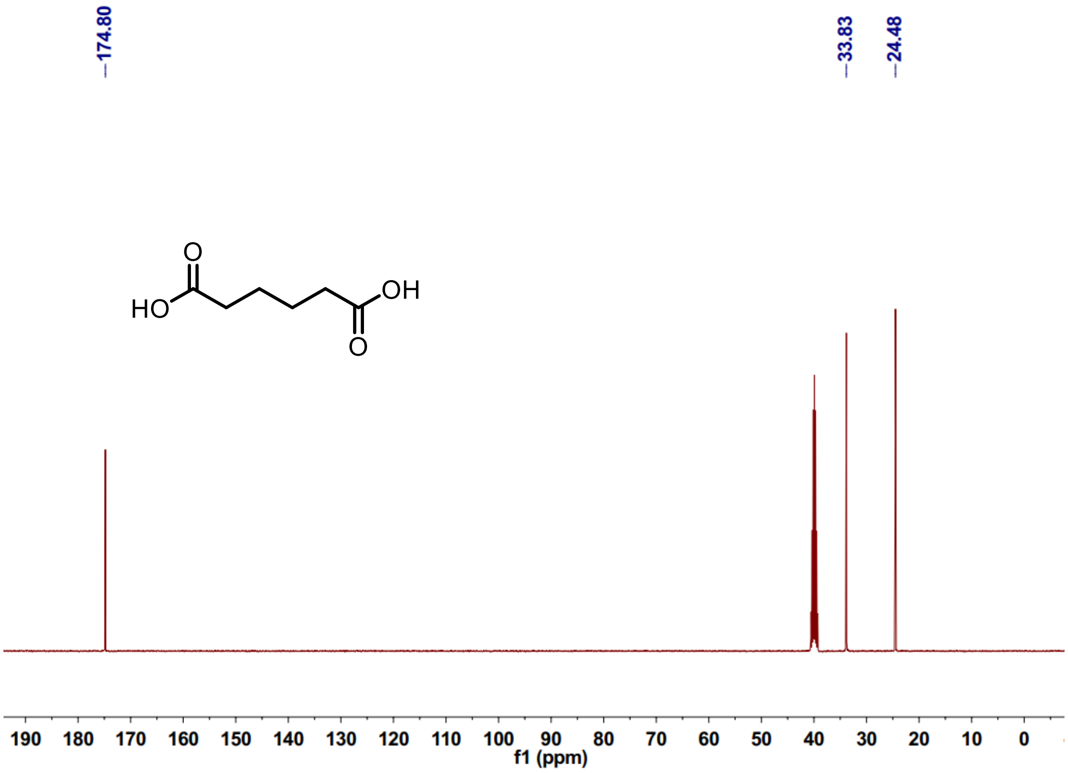

Supplementary Figure 42 ^{13}C-NMR spectrum of adipic acid in DMSO.

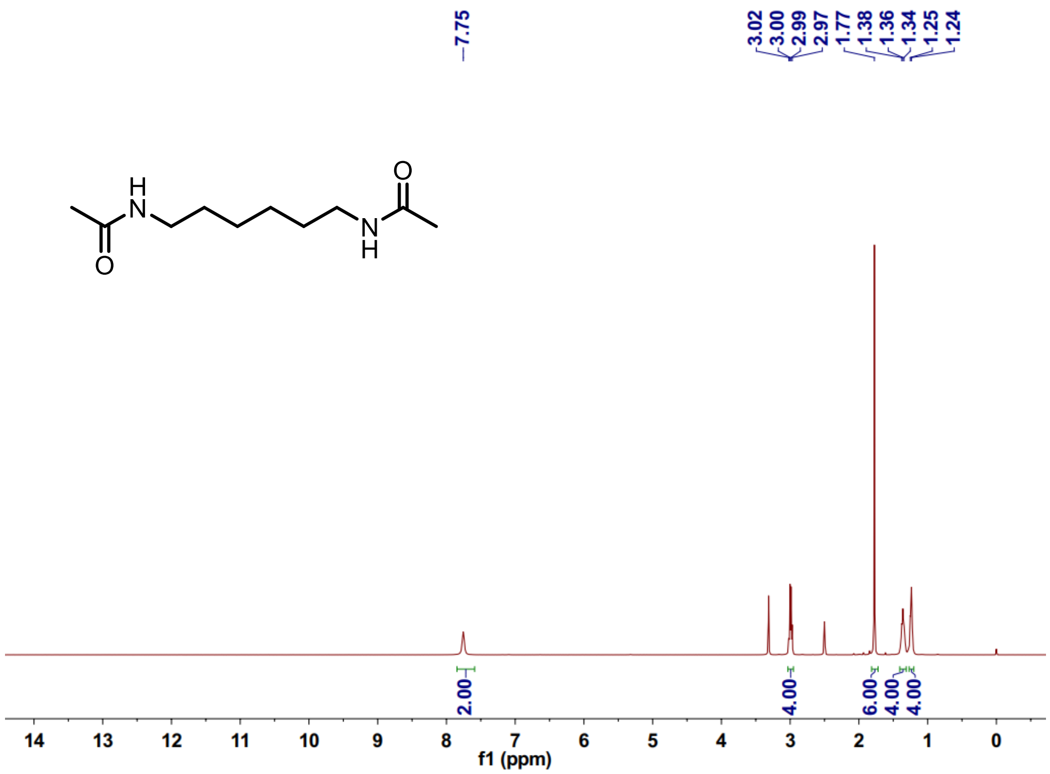

Supplementary Figure 43 ^{1}H-NMR spectrum of diacetyl hexamethylene diamine in DMSO.

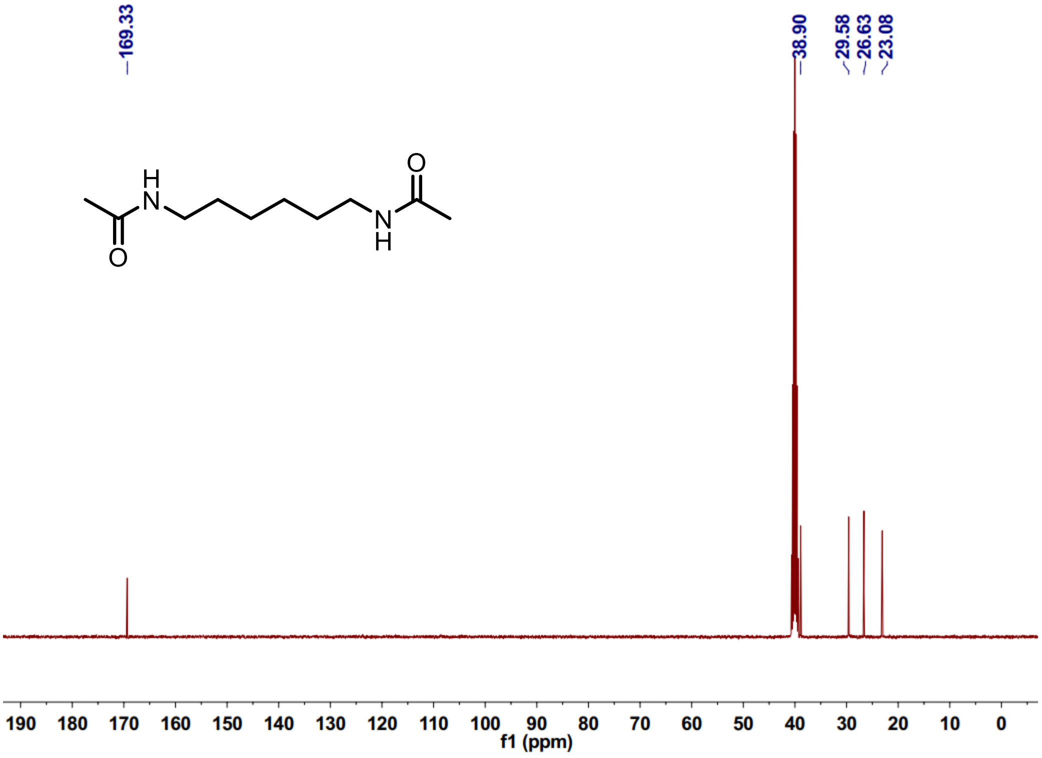

Supplementary Figure 44 ^{13}C-NMR spectrum of diacetyl hexamethylene diamine in DMSO.

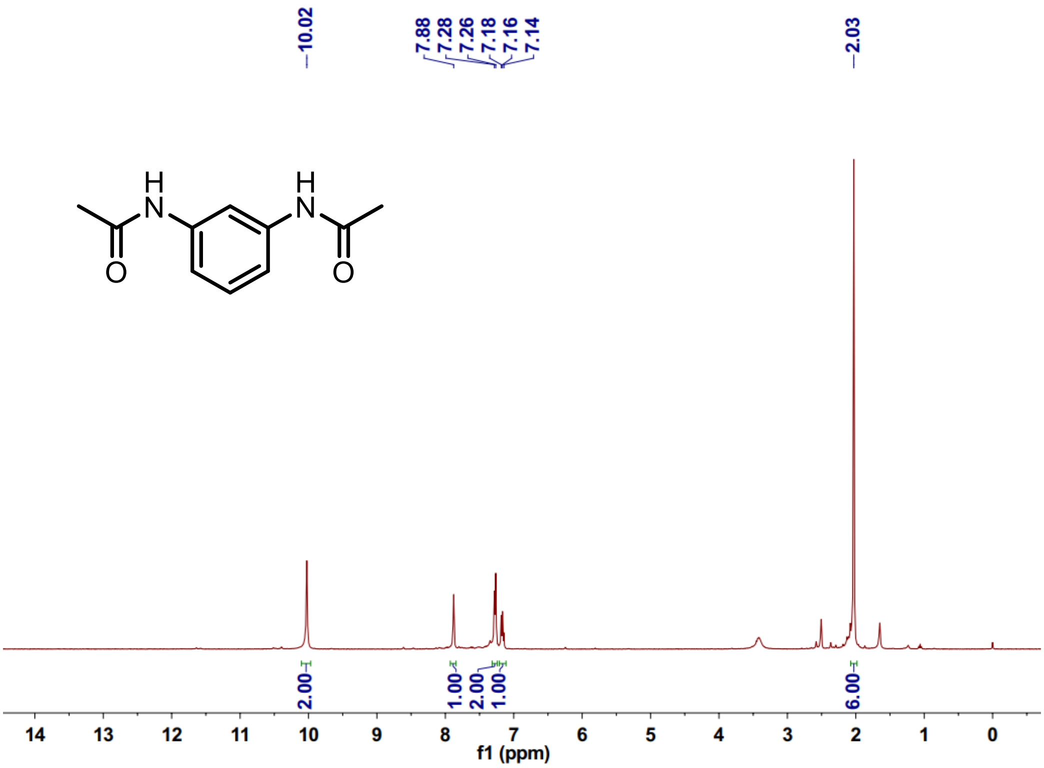

Supplementary Figure 45 ^{1}H-NMR spectrum of acetanilide in DMSO.

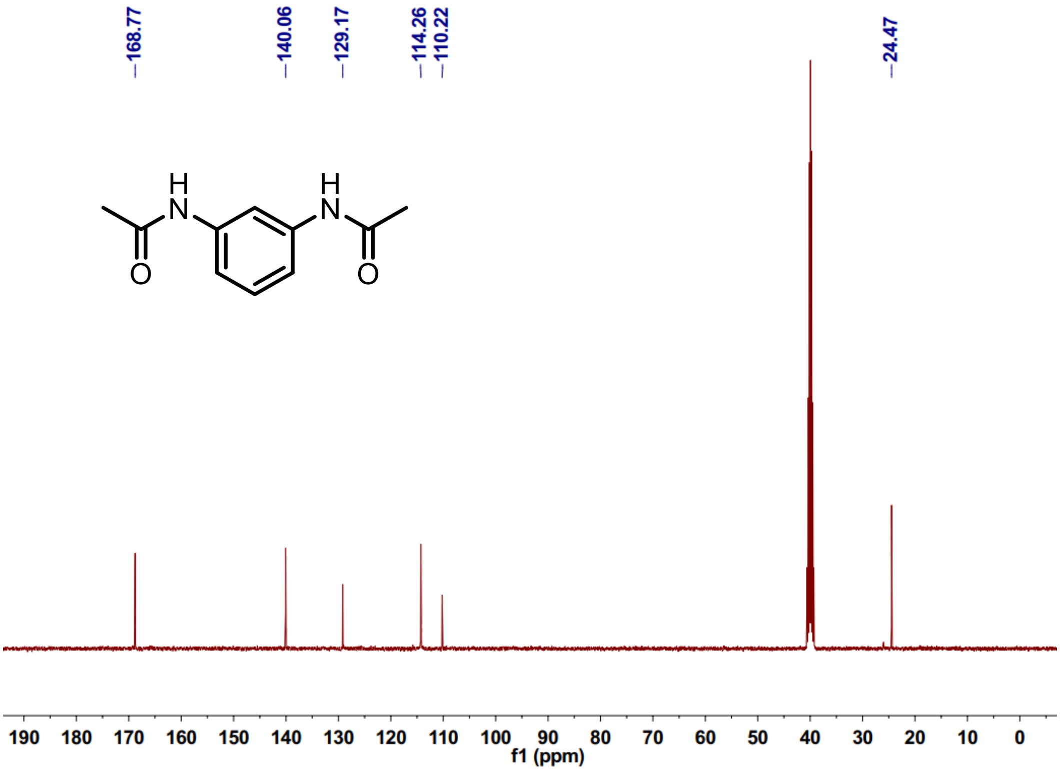

Supplementary Figure 46 ^{13}C-NMR spectrum of acetanilide in DMSO.

Supplementary Note 4: Process details and descriptions for Case 1 and Case 2 in ASPEN

In this simulation, poly-NRTL physical property method was selected to deal with PET depolymerization and polymerization process [7-10] (RBATCH1, CSTR1-2, RPLUG), At the same time, for other processes such as distillation, NRTL physical property method was used when dealing with acetic acid aqueous solution system, NRTL-HOC physical property method is used to accurately simulate.

Case 1(Supplementary Figure 47; Supplementary Figure 49): The discarded PET fragments were mixed with acetic acid and then enter the intermittent reaction kettle (RBATCH1). The depolymerization was carried out at 280 °C for 2 h. Then the reacted material was cooled (the heat from this process can produce high-pressure steam) and filtered to separate solid TPA. The liquid phase entered the rectification column (RADFRAC1) to further separate EGDA and HOAc. A small amount of PET oligomer remained in the liquid phase of the tower kettle (OLI-PET1) after depolymerization. Gaseous EGDA was extracted near the sideline of the bottom and a small amount of EGDA was separated from the oligomer by flash evaporation (FLASH1). The second section is EGDA hydrolysis, where the combined EGDA was mixed with a hydrochloric acid solution (HCl-H_2O) with a concentration of 0.2 M and then hydrolyzed in an intermittent reactor (RBATCH2) with hydrolysis kinetics data from Julio F. Mata-Segreda et al [11]. The hydrolysate was separated from the mixture of HCl, H_2O, HOAc and EG by a distillation column (RADFRAC2), from strong to weak the order of volatility is HCl>H_2O>HOAc>EG. Compared with H_2O/HOAc, HOAc/EG has a greater volatility difference and is easier to separate. At the same time, to avoid subsequent re-esterification inversion, high-purity EG should be separated as far as possible in the presence of HCl. Therefore, it is selected to be separated from HOAc/EG here, HOAc is the light key component, and EG is the heavy key component. To save energy, the fraction at the top of the tower directly entered the next distillation column (RADFRAC3) without cooling. The high purity acetic acid (HOAC2) at the bottom of the tower (RADFRAC3) has a high recovery rate which can be returned to the first section for re-depolymerization of PET. To further reduce energy consumption, RADFRAC3 uses heat pump distillation technology. Similarly, hydrochloric acid aqueous solution (R-H_2O) at the top of the tower can also be recovered and returned to the second section for EGDA hydrolysis. The third section is re-polymerization, where high-purity EG was mixed with TPA (when dealing with textiles and colored bottles, TPA from SEP1 was mixed with activated carbon (ACT-C in Supplementary Figure 49) and high temperature water for decolorization (DECOLOER in Supplementary Figure 48) to obtain TPA of qualified chromaticity) and then esterified in an all-mixing reactor (CSTR1, CSTR2). After each reactor, residual EG was collected and separated from water in RADFRAC4 and returned to the aggregate. Finally, PET polyester with high molecular weight (PRO-PET) was obtained by high vacuum operation (280 °C, 1 mmHg) in the post-polymerization reactor (RPLUG), and the kinetic data of the polymerization process were obtained from Ref. 10.

Case 2 is very similar to Case 1 except for Section 2 (Supplementary Figure 48; Supplementary Figure 50). Section 3 is a re-polymerization stage. Since there is no model for polymerization between EGDA and TPA, we estimated this process based on the dynamic data from the polymerization process between EG and TPA. At the same time, new polymer segments and oligomers are redefined (Supplementary Table 24).

Supplementary Note 5: Process details and descriptions for the decolorization process in ASPEN

We simulate three decolorization methods for dark TPA.

Method 1 (Supplementary Figure 51): Activated carbon and crude TPA were added to the NaOH solution, and the container was heated to 110 °C for 20 mins. Here NaOH reacted with TPA to form terephthalic anions and Na^+, which dissolved in water. Activated carbon with pigment was filtered and left behind. Since pigment components are difficult to define in Aspen, according to the final yield, 4% of TPA was treated as pigment which was trapped with the activated carbon. The filtrate was mixed with HCl solution to regenerate TPA, and purified TPA was obtained after filtration again. The waste liquid was neutralized by NaOH and discharged.

Method 2 (Supplementary Figure 52): Activated carbon and crude TPA were added to the N, N-dimethylacetamide, and the container was heated to 150 °C for 20 mins. Similarly, 5% of TPA was trapped with activated carbon. TPA/DMAC adduct crystals were precipitated from the filtrate after freeze-recrystallization. (The energy required for cooling in this step was measured in electricity) After filtration and adding water, the adduct was dissociated to obtain TPA and DMAC. DMAC was separated by distillation and reused.

Method 3 (Supplementary Figure 53): Activated carbon and crude TPA were added to the water, and the container was heated to 280 °C for 30 mins. 8% of TPA was trapped with activated carbon. After filtration and cooling, pure TPA is obtained.

Supplementary Note 6: Life-cycle assessment

The goal and scope of the LCA study in this work are shown in Supplementary Table 11. Besides, the system boundary of LCA was set as "cradle to gate" (i.e., collection and transportation of waste PET; Production of chemical raw materials; And the whole process of post-depolymerization and re-polymerization to produce recycled amorphous PET resin in forms of chips) because the subsequent use and disposal of various PET products vary widely. Since there is no data on the pretreatment process of waste PET plastic collected in the early stage, we can only assume that it is consistent with mechanical recycling [12]. At the same time, our distribution principle followed the “cut-off” rule that the first life cycle (original plastic) and the second life cycle (recycled plastic) are independent of each other. For inventory analysis, our material balance and energy balance were derived from actual experimental results and Aspen simulation results. These were our “prospective processes”, and the functional unit was set at 1 kg of amorphous PET resin in forms of chips (PRO-PET in Aspen Plus). The impact assessment was carried out on professional software OpenLCA 1.10.3, and the assessment methods were IPCC AR5 for GWP, CML-IA baseline for NREU. “Background process” data were obtained from the database Ecoinvent V.3.7.1. The carbon footprint corresponding to electricity, steam, cooling water, and chemical raw materials and their reference sources were detailed in Supplementary Table 16-17. The recently held 26th UN Climate Change Conference of the Parties pointed out that global warming from greenhouse gas emissions is likely to have "profound implications for current and future generations". Many countries, including China, have reached a consensus on the implementation rules of the Paris Agreement. Focusing on the greenhouse effect and achieving carbon reduction is an important goal in our LCA study. Herein, we mainly focused on non-renewable energy use (NREU, the result is expressed by MJ per kg amorphous PET resin in forms of chips) and Global Warming Potential (GWP, the result is expressed by kg CO_2 equivalent/kg amorphous PET resin in forms of chips, namely kg CO_2-eq per kg amorphous PET resin in forms of chips).

Note: In the mechanical shredding process, due to the high-water tolerance of the follow depolymerization system (i.e., complete depolymerization can be guaranteed even when the mass ratio of water to PET is up to 25%), the drying here means drain off instead of heating. In the extrusion process, the high-temperature PET resin from the reactor is directly involved in the granulation process, so there is no additional heat supply. In the decolorization process, the intercepted activated carbon can be recycled, which corresponds to the "Treatment of spent activated carbon" process in OpenLCA. The result of this activity is 0.9 kg of reactivated carbon from 1 kg of spent activated carbon.

**Supplementary Table 11** Goal and scope definition of this LCA study	
Goal	
Reason for conducting the study	1. This ex-ante LCA study will focus on carbon dioxide emissions and fossil energy consumption.<br>2. To assess the global warming potential (GWP) and Non-renewable energy use (NREU) of recycling of PET bottles via acetolysis, and compare it with published commercial depolymerization protocols in Europe or China (Fig. 5a b in main text);<br>3. To assess the global warming potential (GWP) and Non-renewable energy use (NREU) of recycling of PET textiles via acetolysis, and compare it with fossil-based PET resin production in Europe or China (Fig. 5c in the main text).
Audience	Industrial stakeholders, the research community, and the public
Application	Provide technical and theoretical support for carbon emission reduction policies and circular economy
The intention of using results in a comparative study	Yes, the results are to be compared and disclosed to the public through this article publication
Scope	
Product system	The waste PET depolymerization section with the recycled amorphous PET resin in forms of chips production is based on Case 1 and Case 2 in EU and CN, respectively.
Functional unit	1 kg of amorphous PET resin in forms of chips
System boundary	Cradle-to-factory gate, See Fig. 4 in the main text
Allocation	Waste PET cut-off, all environmental effects are allocated to amorphous PET resin chips.
Assumptions	(I) The pre-treatment discharge of waste PET is consistent with the mechanical method<br>(II) This system deals with 12,500 kg waste PET per hour over 8,000 hours per year<br>(III) This system is located in European countries or China<br>(IV) Status-quo average technology as of 2020 in the background.
Requirements on data and quality	Foreground material and energy consumption data were obtained from simulation in Aspen Plus and the background processes were chosen based on Ecoinvent V.3.7.1 in Open LCA 1.10.3 to cover the technological and geographical representativeness
LCIA methodology	IPCC AR5 for GWP; CML-IA baseline for NREU;
Impact categories assessed in the study	(I) Global Warming Potential (GWP, 100a), kg CO_2 equivalent;<br>(II) Non-renewable energy use (NREU), MJ;
Limitations	In addition to the above-mentioned assumptions, the following aspects are not assessed in this study:<br>Plant construction and equipment maintenance.
Report requirements	To present the outcome via journal publication which is openly accessible to everyone.

Supplementary Table 12 Cradle-to-factory gate LCA results of acetolysis of post-consumer transparent PET bottles, functional unit = 1 kg amorphous PET resin in forms of chips.

Section	Case 1 (EU)		Case 2 (EU)		Case 1 (CN)		Case 2 (CN)	
	NREU	GWP	NREU	GWP	NREU	GWP	NREU	GWP
**Mechanical shredding**	4.35[a]	0.2999[a]	4.35[a]	0.2999[a]	5.29[b]	0.4594[b]	5.29[b]	0.4594[b]
**Acetolysis**	2.11[c]	0.1531[c]	2.30[d]	0.1592[d]	2.31[e]	0.2027[e]	2.51[f]	0.2108[f]
**EGDA hydrolysis**	4.29[g]	0.3723[g]	-	-	7.95[h]	0.9009[h]	-	-
**Repolymerization**	2.79[i]	0.2480[i]	3.81[j]	0.3696[j]	5.49[k]	0.6338[k]	7.51[l]	0.8674[l]
**Extrusion**	2.51[m]	0.2129[m]	2.51[m]	0.2129[m]	4.56[n]	0.5100[n]	4.56[n]	0.5100[n]
**Total**	16	1.29	13	1.04	26	2.71	20	2.05

*Units: NREU: MJ / kg amorphous PET resin in forms of chips; GWP: kg $CO_{2\text{-eq}}$ / kg amorphous PET resin in forms of chips.

Supplementary Table 13 Superscript index table of Supplementary Table 12

	Interpretation	Page
a	The NREU and GWP of mechanical shredding in Case 1 (EU) and Case 2 (EU) can be obtained in Supplementary Table 18	S41
b	The NREU and GWP of mechanical shredding in Case 1 (CN) and Case 2 (CN) can be obtained in Supplementary Table 18	S41
c	The NREU and GWP of acetolysis (Section 1) in Case 1 (EU) can be obtained in Supplementary Table 23-I	S48
d	The NREU and GWP of acetolysis (Section 1) in Case 2 (EU) can be obtained in Supplementary Table 28-I	S53
e	The NREU and GWP of acetolysis (Section 1) in Case 1 (CN) can be obtained in Supplementary Table 23-I	S48
f	The NREU and GWP of acetolysis (Section 1) in Case 2 (CN) can be obtained in Supplementary Table 28-I	S53
g	The NREU and GWP of EGDA hydrolysis (Section 2) in Case 1 (EU) can be obtained in Supplementary Table 23-II	S48
h	The NREU and GWP of EGDA hydrolysis (Section 2) in Case 1 (CN) can be obtained in Supplementary Table 23-II	S48
i	The NREU and GWP of repolymerization (Section 3) in Case 1 (EU) can be obtained in Supplementary Table 23-III	S48
j	The NREU and GWP of repolymerization (Section 3) in Case 2 (EU) can be obtained in Supplementary Table 28-II	S53
k	The NREU and GWP of repolymerization (Section 3) in Case 1 (CN) can be obtained in Supplementary Table 23-III	S48
l	The NREU and GWP of repolymerization (Section 3) in Case 2 (CN) can be obtained in Supplementary Table 28-II	S53
m	The NREU and GWP of extrusion in Case 1 (EU) / Case 2 (EU) can be obtained in Supplementary Table 37	S67
n	The NREU and GWP of extrusion Case 1 (CN) / Case 2 (CN) can be obtained in Supplementary Table 37	S67

Supplementary Table 14 Cradle-to-factory gate LCA results of acetolysis of post-consumer PET textiles, functional unit = 1 kg amorphous PET resin in forms of chips.

Section	Case 1 (EU)		Case 2 (EU)		Case 1 (CN)		Case 2 (CN)	
	NREU	GWP	NREU	GWP	NREU	GWP	NREU	GWP
**Mechanical shredding**	5.30[a]	0.3822[a]	5.30[a]	0.3822[a]	6.42[b]	0.5738[b]	6.42[b]	0.5738[b]
**Acetolysis**	2.12[c]	0.1537[c]	2.39[d]	0.1626[d]	2.32[e]	0.2035[e]	2.61[f]	0.2154[f]
**Decolorization**	1.62[g]	0.2241[g]	1.61[h]	0.2228[h]	6.54[i]	0.8862[i]	6.51[j]	0.8818[j]
**EGDA hydrolysis**	4.31[k]	0.3738[k]	-	-	7.98[l]	0.9045[l]	-	-
**Repolymerization**	2.79[m]	0.2477[m]	3.80[n]	0.3382[n]	5.48[o]	0.6324[o]	7.49[p]	0.8654[p]
**Extrusion**	2.53[q]	0.2154[q]	2.53[q]	0.2154[q]	4.59[r]	0.5136[r]	4.59[r]	0.5136[r]
**Total**	19	1.60	16	1.32	33	3.71	28	3.05

*Units: NREU: MJ / kg amorphous PET resin in forms of chips; GWP: kg $CO_{2\text{-eq}}$ / kg amorphous PET resin in forms of chips.

Supplementary Table 15 Superscript index table of Supplementary Table 14

	Interpretation	Page
a	The NREU and GWP of mechanical shredding in Case 1 (EU) and Case 2 (EU) can be obtained in Supplementary Table 19	S41
b	The NREU and GWP of mechanical shredding in Case 1 (CN) and Case 2 (CN) can be obtained in Supplementary Table 19	S41
c	The NREU and GWP of acetolysis (Section 1) in Case 1 (EU) can be obtained in Supplementary Table 32-I	S61
d	The NREU and GWP of acetolysis (Section 1) in Case 2 (EU) can be obtained in Supplementary Table 36-I	S66
e	The NREU and GWP of acetolysis (Section 1) in Case 1 (CN) can be obtained in Supplementary Table 32-I	S61
f	The NREU and GWP of acetolysis (Section 1) in Case 2 (CN) can be obtained in Supplementary Table 36-I	S66
g	The NREU and GWP of decolorization in Case 1 (EU) can be obtained in Supplementary Table 32-II	S61
h	The NREU and GWP of decolorization in Case 2 (EU) can be obtained in Supplementary Table 36-II	S66
i	The NREU and GWP of decolorization in Case 1 (CN) can be obtained in Supplementary Table 32-II	S61
j	The NREU and GWP of decolorization in Case 2 (CN) can be obtained in Supplementary Table 36-II	S66
k	The NREU and GWP of EGDA hydrolysis (Section 2) in Case 1 (EU) can be obtained in Supplementary Table 32-III	S62
l	The NREU and GWP of EGDA hydrolysis (Section 2) in Case 1 (CN) can be obtained in Supplementary Table 32-III	S61
m	The NREU and GWP of repolymerization (Section 3) in Case 1 (EU) can be obtained in Supplementary Table 32-IV	S61
n	The NREU and GWP of repolymerization (Section 3) in Case 2 (EU) can be obtained in Supplementary Table 36-III	S66
o	The NREU and GWP of repolymerization (Section 3) in Case 1 (CN) can be obtained in Supplementary Table 32-IV	S61
p	The NREU and GWP of repolymerization (Section 3) in Case 2 (CN) can be obtained in Supplementary Table 36-III	S66
q	The NREU and GWP of extrusion in Case 1 (EU) / Case 2 (EU) can be obtained in Supplementary Table 37	S67
r	The NREU and GWP of extrusion in Case 1 (CN) / Case 2 (CN) can be obtained in Supplementary Table 37	S67

Supplementary Table 16 NREU values of each raw material of PET acetic acid hydrolysis process used in the Open LCA

Process	Value	Unit	Location
Acetic acid production (Celanese process)	38.43051	MJ per kg	Europe
	49.52249		Rest of the world
Tap water production	0.00302	MJ per kg	Europe without Switzerland
	0.00392		Rest of the world
Market group for electricity, low voltage	4.62500	MJ per kwh	Europe
	9.20186		China
Steam production, as an energy carrier, in the chemical industry	1.46170	MJ per MJ	Europe
	1.46650		Rest of the world
water production, deionized (Ion exchange)	0.00537	MJ per kg	Europe without Switzerland
	0.00550		Rest of the world
Chlor-alkali electrolysis, mercury cell | sodium hydroxide, without water	9.83737	MJ per kg	Europe
	15.36107		Rest of the world
Hydrochloric acid production	11.30373	MJ per kg	Europe
	16.57359		Rest of the world
Activated carbon production	99.87865	MJ per kg	Europe
	93.15432		Rest of the world
Treatment of spent activated carbon	7.38692	MJ per kg	Europe
	8.39949		Rest of the world
Heat, district or industrial, natural gas	1.20180	MJ per MJ	Europe without Switzerland
	1.11158		Rest of the world
Sulfuric acid production	4.58669	MJ per kg	Europe
	4.77874		Rest of the world
Transport, freight, lorry >32 metric ton	1.40536	MJ per t*km	Europe
	1.43261		Rest of the world
Dimethylacetamide production	72.94525	MJ per kg	Global
treatment of waste polyethylene terephthalate, sanitary landfill	0.24821	MJ per kg	Global

**Supplementary Table 17** GWP values of each raw material of PET acetic acid hydrolysis process used in the Open LCA			
Process	Value	Unit	Location
Acetic acid production (Celanese process)	1.29232	kg $CO_{2\text{-eq}}$ per kg	Europe
	1.73095		Rest of the world
Tap water production (Conventional treatment)	0.00026	kg $CO_{2\text{-eq}}$ per kg	Europe without Switzerland
	0.00034		Rest of the world
Market group for electricity, low voltage	0.41329	kg $CO_{2\text{-eq}}$ per kwh	Europe
	1.06648		China
Steam production, as an energy carrier, in the chemical industry	0.10339	kg $CO_{2\text{-eq}}$ per MJ	Europe
	0.12099		Rest of the world
water production, deionized (Ion exchange)	0.00044	kg $CO_{2\text{-eq}}$ per kg	Europe without Switzerland
	0.00047		Rest of the world
Chlor-alkali electrolysis, mercury cell | sodium hydroxide, without water	0.89524	kg $CO_{2\text{-eq}}$ per kg	Europe
	1.45887		Rest of the world
Hydrochloric acid production	0.91757	kg $CO_{2\text{-eq}}$ per kg	Europe
	1.45403		Rest of the world
Activated carbon production	7.81673	kg $CO_{2\text{-eq}}$ per kg	Europe
	8.45886		Rest of the world
Treatment of spent activated carbon	0.83255	kg $CO_{2\text{-eq}}$ per kg	Europe
	0.98737		Rest of the world
Heat, district or industrial, natural gas	0.07483	kg $CO_{2\text{-eq}}$ per MJ	Europe without Switzerland
	0.07579		Rest of the world
Sulfuric acid production	0.09529	kg $CO_{2\text{-eq}}$ per kg	Europe
	0.11506		Rest of the world
Transport, freight, lorry >32 metric ton	0.09057	kg $CO_{2\text{-eq}}$ per t*km	Europe
	0.09353		Rest of the world
Dimethylacetamide production	3.33865	kg $CO_{2\text{-eq}}$ per kg	Global
treatment of waste polyethylene terephthalate, sanitary landfill	0.09048	kg $CO_{2\text{-eq}}$ per kg	Global

Supplementary Table 18 Material and energy input-output of Mechanical shredding PET transparent bottles, functional unit = 1 kg PET flakes.

Item	Quantity	Unit
Input:		
Transportation distance	400	km
Waste transparent PET bottles	1088	kg
electricity, low voltage	229.84	kwh
Heat (from natural gas)	2066.87	MJ
Sodium hydroxide	10	kg
Sulfuric acid	20	kg
Output:		
By-products (e.g. caps, labels)	88	kg
PET flakes	1000	kg

Case 1 (EU): NREU 4.3487 MJ / kg PET flakes; GWP 0.29993 kg $CO_{2\text{-eq}}$ / kg PET flakes ;

Case 1 (CN): NREU 5.2851 MJ / kg PET flakes; GWP 0.45936 kg $CO_{2\text{-eq}}$ / kg PET flakes;

Case 2 (EU): NREU 4.3487 MJ / kg PET flakes; GWP 0.29993 kg $CO_{2\text{-eq}}$ / kg PET flakes;

Case 2 (CN): NREU 5.2851 MJ / kg PET flakes ; GWP 0.45936 kg $CO_{2\text{-eq}}$ / kg PET flakes;

All environmental effects are assigned to PET flakes.

Supplementary Table 19 Material and energy input-output of Mechanical shredding PET textiles, functional unit = 1 kg PET strips.

Item	Quantity	Unit
Input:		
Transportation distance	400	km
Waste PET textiles	1361	kg
electricity, low voltage	278	kwh
Heat (from natural gas)	2500	MJ
Sodium hydroxide	10	kg
Sulfuric acid	20	kg
Output:		
By-products (e.g. buttons, zippers)	86	kg
PET strips	1000	kg
Solid waste	275	kg

Case 1 (EU): NREU 5.30054 MJ / kg PET strips; GWP 0.38223 kg $CO_{2\text{-eq}}$ / kg PET strips;

Case 1 (CN): NREU 6.42127 MJ / kg PET strips; GWP 0.57385 kg $CO_{2\text{-eq}}$ / kg PET strips;

Case 2 (EU): NREU 5.30054 MJ / kg PET strips; GWP 0.38223 kg $CO_{2\text{-eq}}$ / kg PET strips;

Case 2 (CN): NREU 6.42127 MJ / kg PET strips; GWP 0.57385 kg $CO_{2\text{-eq}}$ / kg PET strips;

All environmental effects are assigned to PET strips.

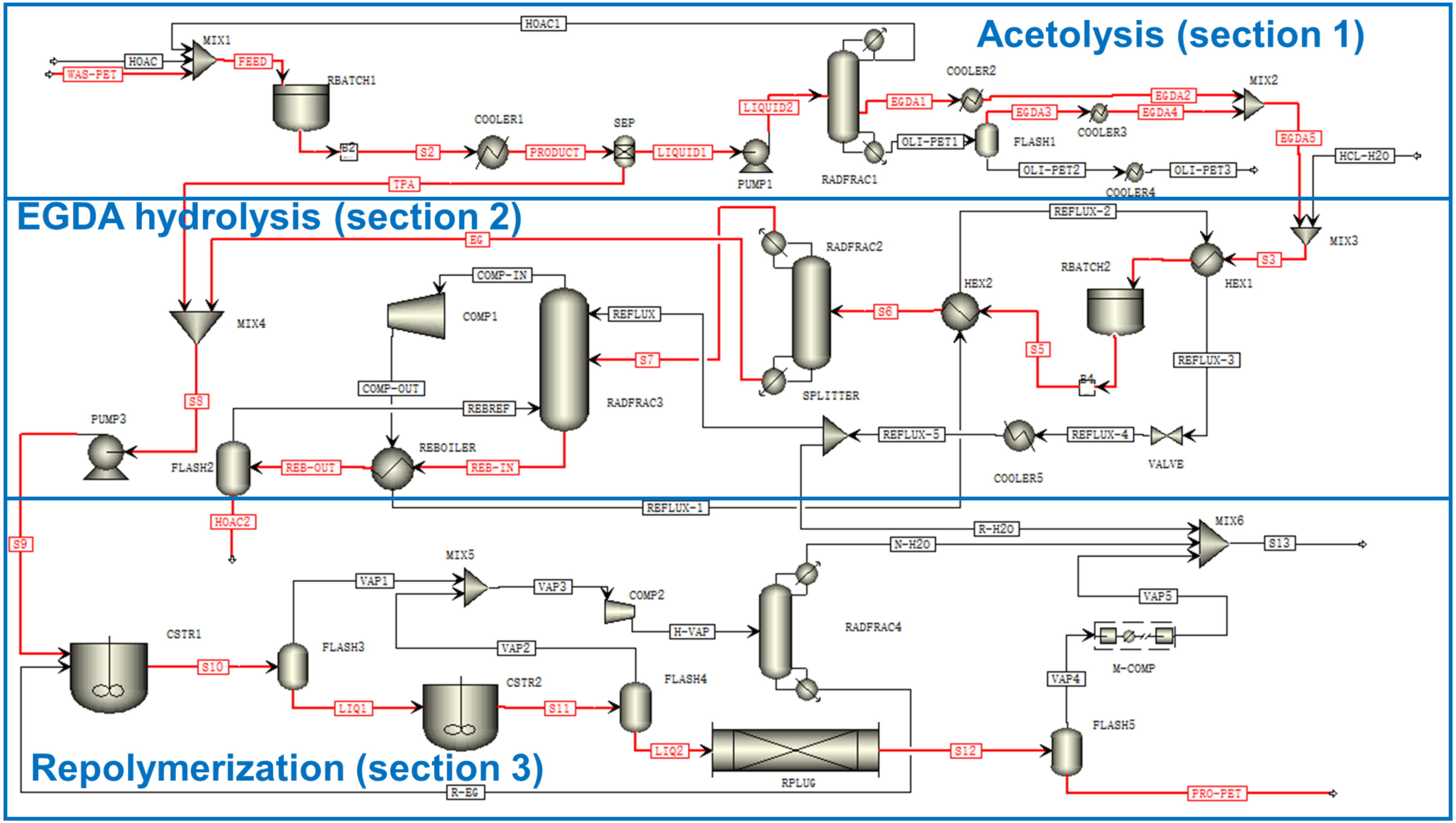

Supplementary Figure 47 Flowsheet diagram from Aspen model for Case 1 (transparent bottles)

Supplementary Table 20 Mass balance of Case 1 (transparent bottles)

Para.	HOAC	WAS-PET	HOAC1	S2	PRODUCT	LIQUID1	TPA	LIQUID2	EGDA1	OLI-PET1	EGDA2	EGDA3	EGDA4	OLI-PET2	OLI-PET3
Temp./°C	25.00	25.00	118.23	280.00	30.10	30.10	30.10	30.10	216.03	216.03	188.35	224.69	190.11	224.69	35.00
Pres./kPa	101.33	101.33	102.00	3240.00	110.00	110.00	110.00	121.59	122.27	122.27	101.33	10.00	101.33	10.00	101.33
W-PET	0	12500.00	0	68.47	68.47	68.47	0	68.47	6.67E-22	68.47	6.67E-22	1.75E-76	1.75E-76	68.47	68.47
EGDA	0	0	17.89	9471.87	9471.87	9471.87	0	9471.87	9357.45	96.52	9357.45	94.10	94.10	2.42	2.42
TPA	0	0	0	10747.10	10747.10	0	10747.10	0	0	0	0	0	0	0	0
HOAC	7812.20	0	54517.00	54559.70	54559.70	54559.70	0	54559.70	42.54	0.076	42.54	0.076	0.076	0.00035	0.00035
H_2O	0	0	0	0	0	0	0	0	0	0	0	0	0	0	0
HCL	0	0	0	0	0	0	0	0	0	0	0	0	0	0	0
EG	0	0	0	0	0	0	0	0	0	0	0	0	0	0	0
PET	0	0	0	0	0	0	0	0	0	0	0	0	0	0	0
BHET	0	0	0	0	0	0	0	0	0	0	0	0	0	0	0

the unit in the material list is kg/h

**Supplementary Table 20** continued															
Para.	EGDA5	HCL-H2O	S3	S4	S5	S6	S7	EG	REFLUX	COMP-IN	COMP-OUT	REFLUX-1	REB-IN	REB-OUT	HOAC2
Temp./°C	188.32	25.00	42.27	90.00	90.00	125.82	105.91	205.89	88.33	99.55	259.51	138.72	123.45	123.47	123.47
Pres./kPa	101.33	101.33	101.33	101.33	121.59	116.52	110.00	130.00	100.00	100.00	350	350	120	120	120
W-PET	6.67E-22	0	6.67E-22	6.67E-22	0	0	0	0	0	0	0	0	0	0	0
EGDA	9451.55	0	9451.55	9451.55	0.27	0.27	1.04E-06	0.27	1.15E-42	2.32E-42	2.32E-42	2.32E-42	2.54E-06	2.54E-06	1.04E-06
TPA	0	0	0	0	0	0	0	0	0	0	0	0	0	0	0
HOAC	42.62	0	42.62	42.62	7810.00	7810	7809.48	0.52	5.42E-08	1.09E-07	1.09E-07	1.09E-07	199736	199736	7809.29
H_2O	0	38375.20	38375.20	38375.20	36045.10	36045.10	36045.1	1.46E-10	35440.40	71451.50	71451.50	71451.50	1019.65	1019.65	33.90
HCL	0	279.60	279.60	279.60	279.60	279.60	279.60	8.68E-61	275.17	554.77	554.77	554.77	6.55E-113	6.55E-113	0
EG	0	0	0	0	4014.06	4014.06	5.55E-06	4014.06	2.11E-48	4.25E-48	4.25E-48	4.25E-48	1.06E-05	1.06E-05	5.55E-06
PET	0	0	0	0	0	0	0	0	0	0	0	0	0	0	0
BHET	0	0	0	0	0	0	0	0	0	0	0	0	0	0	0

**Supplementary Table 20** continued															
Para.	REBREF	REFLUX-2	REFLUX-3	REFLUX-4	REFLUX-5	S8	S9	R-EG	S10	LIQ1	VAP1	S11	LIQ2	VAP2	VAP3
Temp./°C	123.47	136.05	120.20	97.87	88.33	99.92	100.25	201.46	260	260	260	260	260	260	255.31
Pres./kPa	120.00	344.50	344.50	100.00	100.00	110.00	810.60	121.59	810.60	810.60	810.60	101.33	101.33	101.33	101.33
W-PET	0	0	0	0	0	0	0	0	0	0	0	0	0	0	0
EGDA	1.50E-06	2.32E-42	2.32E-42	2.32E-42	2.32E-42	0.27	0.27	1.12	1.38	0.85	0.53	0.85	0.27	0.58	1.12
TPA	0	0	0	0	0	10747.10	10747.10	15.65	1258.03	1250.27	7.76	388.19	380.30	7.89	15.65
HOAC	191927	1.09E-07	1.09E-07	1.09E-07	1.09E-07	0.52	0.52	0.049	0.57	0.15	0.41	0.15	0.014	0.15	0.55
H_2O	985.71	71451.50	71451.50	71451.50	71451.50	1.46E-10	1.46E-10	1.04	1707.06	254.59	1452.47	510.43	22.72	487.70	1940.17
HCL	0	554.77	554.77	554.77	554.77	8.68E-61	8.68E-61	0	8.68E-61	0	0	0	0	0	0
EG	5.01E-06	4.25E-48	4.25E-48	4.25E-48	4.25E-48	4014.06	4014.06	602.27	932.65	550.42	382.23	307.91	87.87	220.04	602.27
PET	0	0	0	0	0	0	0	0	10872.2	10872.2	1.29E-76	12023.7	12023.7	6.26E-76	7.55E-76
BHET	0	0	0	0	0	0	0	2.45	612.69	611.97	0.72	309.20	307.48	1.72	2.45

Supplementary Table 20 continued

Para.	H-VAP	N-H2O	S12	VAP4	PRO-PET	R-H2O	VAP5	S13
Temp./°C	269.18	100.02	280	258.93	258.93	88.33	292.72	97.86
Pres./kPa	111.46	101.33	0.13	0.13	0.13	100	101.33	100
W-PET	0	0	0	0	0	0	0	0
EGDA	1.12	8.79E-10	0.27	0.27	0.0002	1.17E-42	0.27	0.27
TPA	15.65	1.60E-43	0.0006	0.0005	4.38E-05	0	0.0005	0.0005
HOAC	0.55	0.50	0.014	0.014	2.38E-06	5.51E-08	0.014	0.52
H_2O	1940.17	1939.14	379.70	379.67	0.029	36011.5	379.67	38330.30
HCL	0	0	0	0	0	279.60	0	279.60
EG	602.27	1.83E-09	0	0	0	2.14E-48	0	1.83E-09
PET	7.55E-76	0	12442	4.48E-73	12442	0	4.48E-73	4.48E-73
BHET	2.45	1.50E-83	0.39	0.32	0.071	0	0.32	0.32

Supplementary Table 21 Utility of Case 1 (transparent bottles)

Unit processes used	Type of Utility	Quantity	Function
RBATCH1	Electricity	502.01 kwh	Heating
COOLER1	U-2	12139.10 kg/h	Cool the product and co-produce steam for RADFRAC1
PUMP1	Electricity	0.34 kwh	Increase the pressure of the fluid
RADFRAC1	U-1; U-3	U-1: 390571 kg/h;<br>U-3: 21669.20 kg/h	U-1: Cool the top fraction;<br>U-3: Heat the tower kettle;
FLASH1	U-3	16.70 kg/h	Heating
COOLER2	U-1	53111 kg/h	Cooling
COOLER3	U-1	547.62 kg/h	Cooling
COOLER4	U-1	16.87 kg/h	Cooling
RBATCH2	Electricity	3246.22 kwh	Heating
COOLER5	U-1	175167 kg/h	Cooling
RADFRAC2	U-1; U-3	U-1: 161412 kg/h<br>U-3: 2905.72 kg/h	U-1: Cool the top fraction;<br>U-3: Heat the tower kettle;
COMP1	Electricity	6678.85 kwh	Increase the pressure of the fluid
PUMP3	Electricity	2.91 kwh	Increase the pressure of the fluid
CSTR1	Electricity	5846.98 kwh	Heating
CSTR2	Electricity	716.11 kwh	Heating
RPLUG	Electricity	535.55 kwh	Heating
COMP2	Electricity	21.23 kwh	Increase the pressure of the fluid
RADFRAC4	U-1; U-3	U-1: 28005.90 kg/h;<br>U-3: 301.15 kg/h	U-1: Cool the top fraction;<br>U-3: Heat the tower kettle;
MCOMP	U-1; Electricity	U-1: 11882.90 kg/h<br>Electricity: 213.44 kwh	U-1: Compressor interstage cooling;<br>Electricity: Increase the pressure

**Supplementary Table 22** Details of the utilities of Case 1 (transparent bottles)		
Type of utility used	Initial and final state of utility	
	Initial	Final
U-1	20 ℃; 1 atm; liquid phase	35 ℃; 1 atm; liquid phase
U-2	20 ℃; 1 atm; liquid phase	250 ℃; 39.2 atm; gaseous phase
U-3	250 ℃; 39.2 atm; gaseous phase	249 ℃; 38.6 atm; liquid phase
Electricity	-	

Supplementary Table 23-I Energy balance of Case 1 （Transparent bottles）

Equipment	RBATCH1	COOLER1	SEP	PUMP1	RADFRAC1		COOLER2	FLASH1	COOLER3	COOLER4
Cost/kwh	502.01	-9155.76	-2.49	0.24	CON: -6789.89	REB: 10349.00	-923.31	7.97	-9.52	-0.29

Case 1-Acetolysis (section 1) (EU): NREU 2.11427 MJ / kg amorphous PET resin in forms of chips; GWP 0.15307 kg CO_{2-eq} / kg amorphous PET resin in forms of chips;

Case 1-Acetolysis (section 1) (CN): NREU 2.30611 MJ / kg amorphous PET resin in forms of chips; GWP 0.20268 kg CO_{2-eq} / kg amorphous PET resin in forms of chips;

Supplementary Table 23-II continued

Equipment	RBATCH2	PUMP3	RADFRAC2		RADFRAC3	
Cost/kwh	3246.22	2.91	CON: -2806.08	REB: 1387.74	COOLER5: -3045.27	COMP1: 6678.85

Case 1-EGDA hydrolysis (section 2) (EU): NREU 4.28897 MJ / kg amorphous PET resin in forms of chips; GWP 0.37231 kg CO_{2-eq} / kg amorphous PET resin in forms of chips;

Case 1-EGDA hydrolysis (section 2) (CN): NREU 7.94639 MJ / kg amorphous PET resin in forms of chips; GWP 0.90089 kg CO_{2-eq} / kg amorphous PET resin in forms of chips;

Supplementary Table 23-III continued

Equipment	CSTR1	FLASH2	CSTR2	FLASH3	COMP2	RPLUG	FLASH4	RADFRAC4		M-COMP	
Cost/kwh	5846.98	0	716.11	0	21.23	535.55	0	CON: -486.87	REB: 143.38	CON: -206.58	WORK: 213.44

Case 1-Repolymerization (section 3) (EU): NREU 2.78857 MJ / kg amorphous PET resin in forms of chips; GWP 0.24805 kg CO_{2-eq} / kg amorphous PET resin in forms of chips;

Case 1-Repolymerization (section 3) (CN): NREU 5.4869 MJ / kg amorphous PET resin in forms of chips; GWP 0.63382 kg CO_{2-eq} / kg amorphous PET resin in forms of chips;

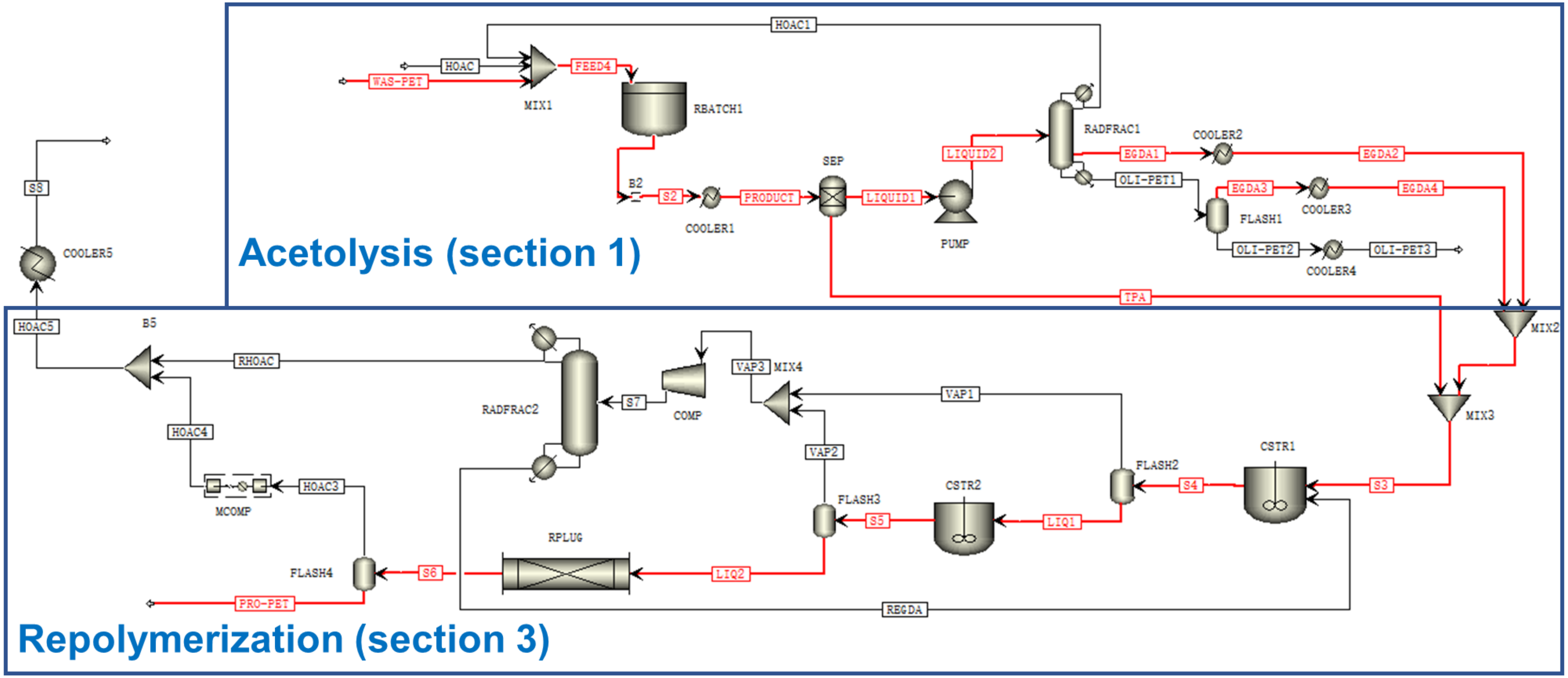

Supplementary Figure 48 Flowsheet diagram from Aspen model for Case 2 (transparent bottles)

**Supplementary Table 24** Properties of the components not included in the Aspen database in Case 2				
Compound	Heat of formation (kJ·kmol^{-1})	Density (kg·m^{-3})	Boiling points (°C)	Molecular weight (g·mol^{-1})
PET[13]	-485510	-	-	192.171
BAET[a]	-121527	1732.58	371.02	338.31
T-EGDA[b]	-	-	-	-

[a] BAET is the hypothesized intermediate during polymerization of the EGDA and TPA, it's estimated by the NIST ThermoData engine in Aspen Plus.

[b] T-EGDA is the hypothetical terminal segment of polymer. It's estimated using Flory-Huggins theoretical calculation[14-15] in Aspen Plus. The structural formula is as follows:

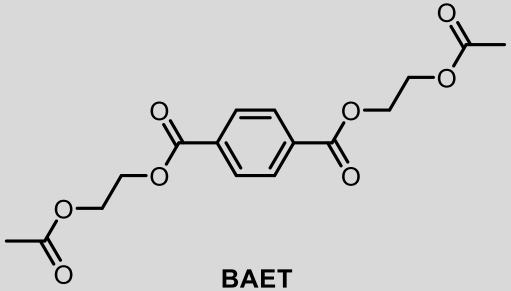

BAET

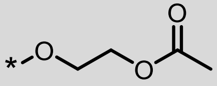

T-EGDA

Supplementary Table 25 Mass balance of Case 2 (transparent bottles)

Para.	HOAC	WAS-PET	HOAC1	S2	PRODUCT	LIQUID1	TPA	LIQUID2	EGDA1	OLI-PET1	EGDA2	EGDA3	EGDA4	OLI-PET2	OLI-PET3	EGDA5
Temp./°C	25.00	25.00	118.29	280.00	30.10	30.00	30.00	30.00	215.73	215.73	188.45	224.69	35.00	224.69	35.00	188.71
Pres./kPa	101.33	101.33	102.00	3240.00	105.00	105.00	105.00	121.59	122.27	122.27	101.33	10.00	101.33	10.00	101.33	101.33
W-PET	0	12500.00	0	67.22	67.22	67.22	0	67.22	6.54E-22	67.22	6.54E-22	1.77E-76	1.77E-76	67.22	67.22	6.54E-22
EGDA	0	0	18.75	9473.67	9473.67	9473.67	0	9473.67	9358.23	96.69	9358.23	94.16	94.16	2.53	2.53	9452.39
TPA	0	0	0	10748.20	10748.20	0	10748.20	0	0	0	0	0	0	0	0	0
HOAC	7812.20	0	54505.90	54547.80	54547.80	54547.80	0	54547.80	41.77	0.075	41.77	0.075	0.075	0.00040	0.00040	41.84
H2O	0	0	0	0	0	0	0	0	0	0	0	0	0	0	0	0
PET	0	0	0	0	0	0	0	0	0	0	0	0	0	0	0	0
BAET	0	0	0	0	0	0	0	0	0	0	0	0	0	0	0	0

Supplementary Table 25 continued

Para.	S3	REGDA	S4	VAP1	LIQ1	S5	VAP2	LIQ2	S6	VAP3	S7	RHOAC	HOAC3	HOAC4	PRO-PET	HOAC5	S8
Temp./°C	115.29	198.60	260.00	251.33	251.33	260.00	260.00	260.00	280.00	251.00	255.22	118.00	264.38	260.18	264.38	118.01	118.01
Pres./kPa	101.33	121.59	810.60	506.63	506.63	101.33	101.33	101.33	0.13	101.33	111.46	101.33	0.13	101.33	0.13	101.33	101.33
W-PET	6.54E-22	0	6.54E-22	0	0	0	0	0	0	0	0	0	0	0	0	0	0
EGDA	9452.39	1294.12	1853.93	777.05	1076.88	750.44	517.07	233.37	1.47E-10	1294.12	1294.12	1.32E-09	1.47E-10	1.47E-10	8.47E-14	1.46E-09	1.46E-09
TPA	10748.20	18.70	1164.31	7.27	1157.04	548.30	11.43	536.86	0.0028	18.70	18.70	2.08E-63	0.0027	0.0027	0.00015	0.0027	0.0027
HOAC	41.84	0.54	5853.81	4453.06	1400.75	1919.13	1744.18	175.95	1552.76	6197.22	6197.22	6196.67	1552.56	1552.56	0.20	7749.24	7749.24
H2O	0	0	0	0	0	0	0	0	0	0	0	0	0	0	0	0	0
PET	0	0	11834.70	1.57E-76	11834.7	12551.9	6.18E-76	12551.9	12491.7	7.75E-76	7.75E-76	0	5.49E-73	5.49E-73	12491.7	5.49E-73	5.49E-73
BAET	0	37.52	886.63	9.52	877.10	576.65	27.99	548.66	1.33	37.52	37.52	1.35E-79	1.31	1.31	0.021	1.31	1.31

**Supplementary Table 26** Utility of Case 2 (transparent bottles)			
Unit processes used	Type of Utility	Quantity	Function
RABTCH1	Electricity	500.58 kwh	Heating
COOLER1	U-2	12137.90 kg/h	Cool the product and co-produce steam for RADFRAC1
PUMP1	Electricity	0.34 kwh	Increase the pressure of the fluid
RADFRAC1	U-1; U-3	U-1: 390381 kg/h;<br>U-3: 21668.10 kg/h	U-1: Cool the top fraction;<br>U-3: Heat the tower kettle;
COOLER2	U-1	51445.20 kg/h	Cooling
FLAH1	U-3	16.70 kg/h	Heating
COOLER3	U-1	1014.36 kg/h	Cooling
COOLER4	U-1	17.21 kg/h	Cooling
CSTR1	Electricity	7868.60 kwh	Heating
CSTR2	Electricity	774.25 kwh	Heating
RPLUG	Electricity	1150.97 kwh	Heating
COMP	Electricity	20.02 kwh	Increase the pressure of the fluid
RADFRAC2	U-1; U-3	U-1: 77108 kg/h;<br>U-3: 361.30 kg/h	U-1: Cool the top fraction;<br>U-3: Heat the tower kettle;
MCOMP	U-1; Electricity	U-1: 15997.20 kg/h<br>Electricity: 274.25 kwh	U-1: Compressor interstage cooling;<br>Electricity: Increase the pressure
COOLER5	U-1	14510.90 kg/h	Cooling

**Supplementary Table 27** Details of the utilities of Case 2 (transparent bottles)		
Type of utility used	Initial and final state of utility	
	Initial	Final
U-1	20 ℃; 1 atm; liquid phase	35 ℃; 1 atm; liquid phase
U-2	20 ℃; 1 atm; liquid phase	250 ℃; 39.2 atm; gaseous phase
U-3	250 ℃; 39.2 atm; gaseous phase	249 ℃; 38.6 atm; liquid phase
Electricity	-	

Supplementary Table 28-I Energy balance of Case 2 (transparent bottles)

Equipment	RBATCH1	COOLER1	SEP	PUMP1	RADFRAC1		COOLER2	FLASH1	COOLER3	COOLER4
Cost/kwh	500.58	-9154.79	-2.49	0.34	CON: -6788.45	REB: 10348.50	-894.62	7.99	-17.64	-0.30

Case 2-Acetolysis (section 1) (EU): NREU 2.30254 MJ / kg amorphous PET resin in forms of chips; GWP 0.15917 kg CO_{2-eq} / kg amorphous PET resin in forms of chips;

Case 2-Acetolysis (section 1) (CN): NREU 2.51363 MJ / kg amorphous PET resin in forms of chips; GWP 0.21075 kg CO_{2-eq} / kg amorphous PET resin in forms of chips;

Supplementary Table 28-II continued

Equipment	CSTR1	FALSH2	CSTR2	FLASH3	COMP	RPLUG	RADFRAC2		FLASH4	M-COMP		COOLER5
Cost/kwh	7868.60	0	774.25	0	20.02	1150.97	CON: -1340.89	REB: 172.54	0	CON: -278.19	REB:274.25	-252.34

Case 2-Repolymerization (section 3) (EU): NREU 3.80839 MJ / kg amorphous PET resin in forms of chips; GWP 0.36965 kg CO_{2-eq} / kg amorphous PET resin in forms of chips;

Case 2-Repolymerization (section 3) (CN): NREU 7.50502 MJ / kg amorphous PET resin in forms of chips; GWP 0.86736 kg CO_{2-eq} / kg amorphous PET resin in forms of chips;

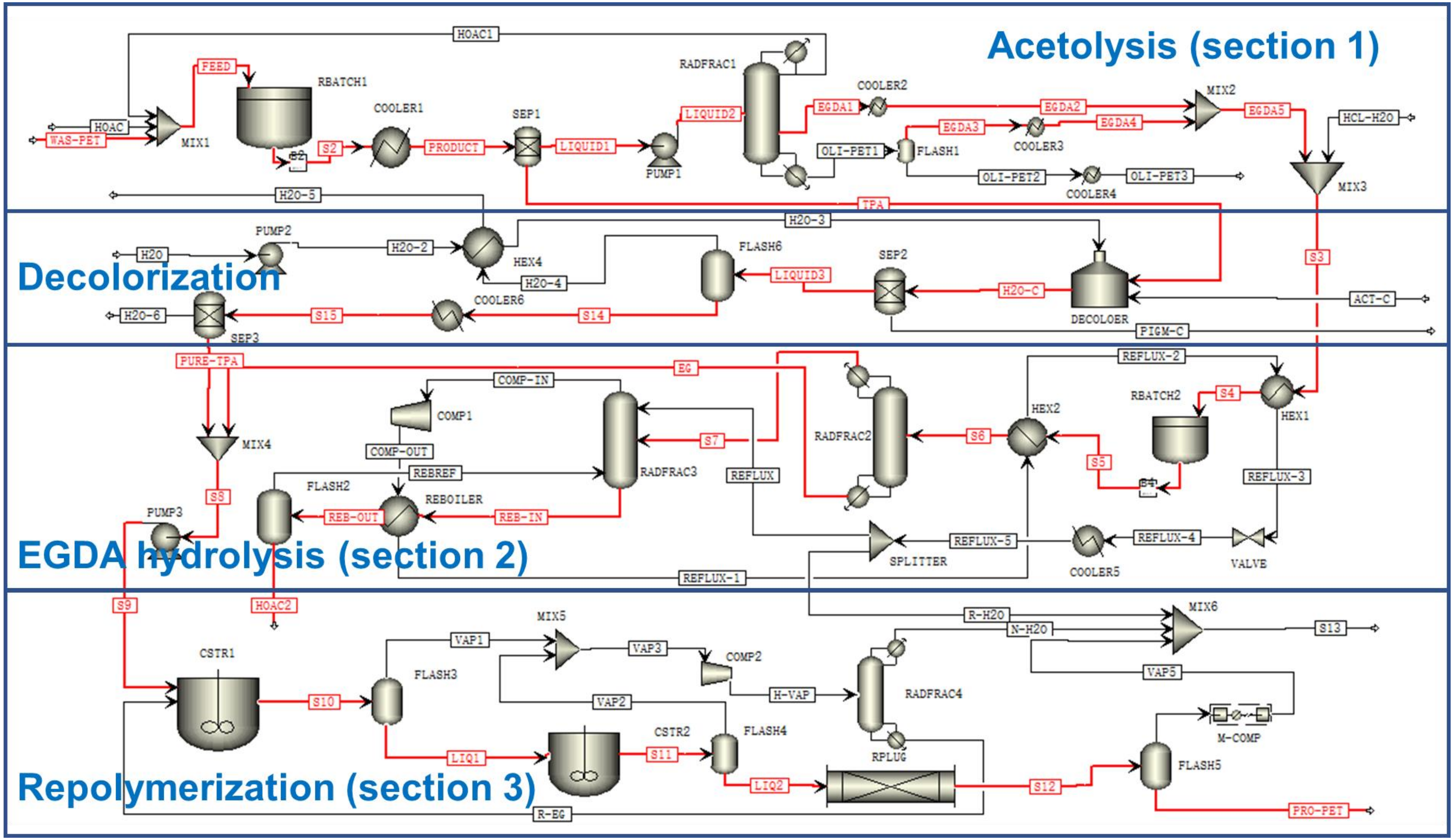

Supplementary Figure 49 Flowsheet diagram from Aspen model for Case 1 (Textile)

Supplementary Table 29 Mass balance of Case 1 (textiles)

Para.	HOAC	WAS-PET	HOAC1	S2	PRODUCT	LIQUID1	TPA	LIQUID2	EGDA1	OLI-PET1	EGDA2	EGDA3	EGDA4	OLI-PET2	OLI-PET3
Temp./°C	25.00	25.00	118.23	280.00	30.10	30.10	30.10	30.10	216.03	216.03	188.35	224.69	190.11	224.69	35.00
Pres./kPa	101.33	101.33	102.00	3240.00	110.00	110.00	110.00	121.59	122.27	122.27	101.33	10.00	101.33	10.00	101.33
W-PET	0	12500.00	0	68.47	68.47	68.47	0	68.47	6.67E-22	68.47	6.67E-22	1.75E-76	1.75E-76	68.47	68.47
EGDA	0	0	17.89	9471.87	9471.87	9471.87	0	9471.87	9357.46	96.52	9357.46	94.10	94.10	2.42	2.42
TPA	0	0	0	10747.10	10747.10	0	10747.10	0	0	0	0	0	0	0	0
HOAC	7812.20	0	54517.00	54559.60	54559.60	54559.60	0	54559.70	42.54	0.076	42.54	0.076	0.076	0.00035	0.00035
H_2O	0	0	0	0	0	0	0	0	0	0	0	0	0	0	0
HCL	0	0	0	0	0	0	0	0	0	0	0	0	0	0	0
EG	0	0	0	0	0	0	0	0	0	0	0	0	0	0	0
PET	0	0	0	0	0	0	0	0	0	0	0	0	0	0	0
BHET	0	0	0	0	0	0	0	0	0	0	0	0	0	0	0
CARBON	0	0	0	0	0	0	0	0	0	0	0	0	0	0	0

the unit in the material list is kg/h

Supplementary Table 29 continued

Para.	ACT-C	H2O-C	LIQUID3	PIGM-C	S14	H2O-4	S15	H2O-5	H2O	H2O-2	H2O-3	H2O-6	PURE-TPA
Temp./°C	25.00	280	280.00	280.00	200.47	200.47	35.00	199.01	25.00	25.32	156.07	35	35
Pres./kPa	101.33	6280.80	6280.80	6280.80	1519.88	1519.88	1418.55	1519.88	101.33	1215.90	1215.90	1418.55	1418.55
W-PET	0	0	0	0	0	0	0	0	0	0	0	0	0
EGDA	0	0	0	0	0	0	0	0	0	0	0	0	0
TPA	0	10747.10	10693.4	53.74	10692.10	1.28	10692.10	1.28	0	0	0	0	10692.10
HOAC	0	0	0	0	0	0	0	0	0	0	0	0	0
H_2O	0	53735.50	53735.50	0	37355.10	16380.40	37355.10	16380.40	53735.50	53735.50	53735.50	37355.10	0
HCL	0	0	0	0	0	0	0	0	0	0	0	0	0
EG	0	0	0	0	0	0	0	0	0	0	0	0	0
PET	0	0	0	0	0	0	0	0	0	0	0	0	0
BHET	0	0	0	0	0	0	0	0	0	0	0	0	0
CARBON	537.36	537.36	0	537.36	0	0	0	0	0	0	0	0	0

Supplementary Table 29 continued

Para.	EGDA5	HCL-H2O	S3	S4	S5	S6	S7	EG	REFLUX	COMP-IN	COMP-OUT	REFLUX-1	REB-IN	REB-OUT	HOAC2
Temp./°C	188.32	25.00	42.27	90.00	89.99	125.82	105.91	205.89	88.33	99.55	259.51	138.72	123.45	123.47	123.47
Pres./kPa	101.33	101.33	101.33	101.33	121.59	116.52	110.00	130.00	100.00	100.00	350	350	120	120	120
W-PET	6.67E-22	0	6.67E-22	6.67E-22	0	0	0	0	0	0	0	0	0	0	0
EGDA	9451.56	0	9451.56	9451.56	0.16	0.16	6.32E-07	0.16	6.99E-43	1.41E-42	1.41E-42	1.41E-42	1.55E-06	1.55E-06	6.32E-07
TPA	0	0	0	0	0	0	0	0	0	0	0	0	0	0	0
HOAC	42.62	0	42.62	42.62	7810.09	7810.09	7809.57	0.51	5.42E-08	1.09E-07	1.09E-07	1.09E-07	199738	199738	7809.36
H_2O	0	38375.20	38375.20	38375.20	36045.10	36045.10	36045.10	1.46E-10	35440.40	71451.50	71451.50	71451.50	1019.34	1019.34	33.89
HCL	0	279.60	279.60	279.60	279.60	279.60	279.60	8.67E-61	275.17	554.77	554.77	554.77	6.53E-113	6.53E-113	0
EG	0	0	0	0	4014.10	4014.10	5.55E-06	4014.10	2.11E-48	4.25E-48	4.25E-48	4.25E-48	1.06E-05	1.06E-05	5.55E-06
PET	0	0	0	0	0	0	0	0	0	0	0	0	0	0	0
BHET	0	0	0	0	0	0	0	0	0	0	0	0	0	0	0
CARBON	0	0	0	0	0	0	0	0	0	0	0	0	0	0	0

Supplementary Table 29 continued

Para.	REBREF	REFLUX-2	REFLUX-3	REFLUX-4	REFLUX-5	S8	S9	R-EG	S10	LIQ1	VAP1	S11	LIQ2	VAP2	VAP3
Temp./°C	123.47	136.05	120.20	97.87	88.33	103.15	100.25	203.90	260	260	260	260	260	260	255.31
Pres./kPa	120.00	344.50	344.50	100.00	100.00	130.00	810.60	121.59	810.60	810.60	810.60	101.33	101.33	101.33	101.33
W-PET	0	0	0	0	0	0	0	0	0	0	0	0	0	0	0
EGDA	9.15E-07	1.41E-42	1.41E-42	1.41E-42	1.41E-42	0.16	0.16	0.24	0.40	0.24	0.15	0.24	0.077	0.17	0.32
TPA	0	0	0	0	0	10629.1	10629.1	15.35	1239.02	1231.37	7.65	378.20	370.50	7.70	15.35
HOAC	191929	1.09E-07	1.09E-07	1.09E-07	1.09E-07	0.51	0.51	3.49E-19	0.51	0.14	0.38	0.14	0.013	0.13	0.50
H_2O	985.42	71451.50	71451.50	71451.50	71451.50	7.59E-08	7.59E-08	9.91E-26	1702.15	253.70	1448.44	508.39	22.61	485.78	1934.22
HCL	0	554.77	554.77	554.77	554.77	3.46E-63	3.46E-63	0	3.46E-63	0	0	0	0	0	0
EG	5.01E-06	4.25E-48	4.25E-48	4.25E-48	4.25E-48	4014.11	4014.11	605.07	938.99	553.99	385.00	311.69	88.88	220.81	607.82
PET	0	0	0	0	0	0	0	0	10828.10	10828.10	1.28E-76	11972.60	11972.60	6.25E-76	7.53E-76
BHET	0	0	0	0	0	0	0	2.50	620.84	620.10	0.73	316.40	314.64	1.77	2.50
CARBON	0	0	0	0	0	0	0	0	0	0	0	0	0	0	0

Supplementary Table 29 continued

Para.	H-VAP	N-H2O	S12	VAP4	PRO-PET	R-H2O	VAP5	S13
Temp./°C	269.15	100.55	280	258.99	258.99	88.33	292.65	97.86
Pres./kPa	111.46	101.33	0.13	0.13	0.13	100	101.33	100
W-PET	0	0	0	0	0	0	0	0
EGDA	0.32	0.086	0.077	0.077	5.93E-05	7.10E-43	0.077	0.16
TPA	15.35	3.87E-32	8.70E-05	8.01E-05	6.63E-06	0	8.01E-05	8.01E-05
HOAC	0.50	0.50	0.013	0.013	2.17E-06	5.51E-08	0.013	0.51
H_2O	1934.22	1934.22	377.28	377.25	0.029	36011.20	377.25	38322.60
HCL	0	0	0	0	0	279.60	0	279.60
EG	607.82	2.74	0	0	0	2.14E-48	0	2.74
PET	7.53E-76	0	12391.10	4.45E-73	12391.10	0	4.45E-73	4.45E-73
BHET	2.50	1.59E-63	0.89	0.72	0.16	0	0.72	0.72
CARBON	0	0	0	0	0	0	0	0

Supplementary Table 30 Details of the utilities of Case 1 (textiles)

Type of utility used	Initial and final state of utility	
	Initial	Final
U-1	20 °C; 1 atm; liquid phase	35 °C; 1 atm; liquid phase
U-2	20 °C; 1 atm; liquid phase	250 °C; 39.2 atm; gaseous phase
U-3	250 °C; 39.2 atm; gaseous phase	249 °C; 38.6 atm; liquid phase
U-4	20 °C; 1 atm; liquid phase	175 °C; 8.8 atm; gaseous phase
Electricity	-	

Supplementary Table 31 Utility of Case 1 (textiles)

Unit processes used	Type of Utility	Quantity	Function
RABTCH1	Electricity	502.01 kwh	Heating
COOLER1	U-2	12139.10 kg/h	Cool the product and co-produce steam for RADFRAC1
PUMP1	Electricity	0.24 kwh	Increase the pressure of the fluid
RADFRAC1	U-1; U-3	U-1: 390452 kg/h;<br>U-3: 21669.20 kg/h	U-1: Cool the top fraction;<br>U-3: Heat the tower kettle;
FLASH1	U-3	16.70 kg/h	Heating
COOLER2	U-1	53094.5 kg/h	Cooling
COOLER3	U-1	547.46 kg/h	Cooling
COOLER4	U-1	16.86 kg/h	Cooling
DECOLOER	Electricity	13289.70 kwh	Heating
COOLER5	U-1	175045 kg/h	Cooling
COOLER6	U-4	12247.4 kg/h	Cooling and co-produce steam
RBATCH2	Electricity	3245.22 kwh	Heating
RADFRAC2	U-1; U-3	U-1: 161363 kg/h<br>U-3: 2905.72 kg/h	U-1: Cool the top fraction;<br>U-3: Heat the tower kettle;
COMP1	Electricity	6678.85 kwh	Increase the pressure of the fluid
PUMP3	Electricity	3.08 kwh	Increase the pressure of the fluid
CSTR1	Electricity	5801.13 kwh	Heating
CSTR2	Electricity	713.48 kwh	Heating
RPLUG	Electricity	535.27 kwh	Heating
COMP2	Electricity	21.18 kwh	Increase the pressure of the fluid
RADFRAC4	U-1; U-3	U-1: 28617 kg/h;<br>U-3: 329.15 kg/h	U-1: Cool the top fraction;<br>U-3: Heat the tower kettle;
MCOMP	U-1; Electricity	U-1: 11807.1 kg/h<br>Electricity: 213.08 kwh	U-1: Compressor interstage cooling;<br>Electricity: Increase the pressure

Supplementary Table 32-I Energy balance of Case 1 (textiles)

Equipment	RBATCH1	COOLER1	SEP	PUMP1	RADFRAC1		COOLER2	FLASH1	COOLER3	COOLER4
Cost/kwh	502.01	-9155.76	0	0.24	CON: -6789.88	REB: 10349.00	-923.30	7.97	-9.52	-0.29

Case 1-Acetolysis (section 1) (EU): NREU 2.12296 MJ / kg amorphous PET resin in forms of chips; GWP 0.15370 kg $CO_{2\text{-eq}}$ / kg amorphous PET resin in forms of chips;

Case 1-Acetolysis (section 1) (CN): NREU 2.31559 MJ / kg amorphous PET resin in forms of chips; GWP 0.20351 kg $CO_{2\text{-eq}}$ / kg amorphous PET resin in forms of chips;

Supplementary Table 32-II continued

Equipment	DECOLOR	PUMP2	COOLER6	FLASH6
Cost/kwh	13289.7	21.88	-9137.58	0

Case 1-Decolorization (EU): NREU 1.61983 MJ / kg amorphous PET resin in forms of chips; GWP 0.22411 kg $CO_{2\text{-eq}}$ / kg amorphous PET resin in forms of chips;

Case 1-Decolorization (CN): NREU 6.54271 MJ / kg amorphous PET resin in forms of chips; GWP 0.88616 kg $CO_{2\text{-eq}}$ / kg amorphous PET resin in forms of chips;

Supplementary Table 32-III continued

Equipment	RBATCH2	PUMP3	RADFRAC2		RADFRAC3	
Cost/kwh	3245.22	3.08	CON: -2806.07	REB: 1387.73	COOLER5: -3044.04	COMP1: 6678.85

Case 1-EGDA hydrolysis (section 2) (EU): NREU 4.30627 MJ / kg amorphous PET resin in forms of chips; GWP 0.37381 kg $CO_{2\text{-eq}}$ / kg amorphous PET resin in forms of chips;

Case 1-EGDA hydrolysis (section 2) (CN): NREU 7.97842 MJ / kg amorphous PET resin in forms of chips; GWP 0.90451 kg $CO_{2\text{-eq}}$ / kg amorphous PET resin in forms of chips;

Supplementary Table 32-IV continued

Equipment	CSTR1	FLASH2	CSTR2	FLASH3	COMP2	RPLUG	FLASH4	RADFRAC4		M-COMP	
Cost/kwh	5801.13	0	713.48	0	21.18	533.27	0	CON: -498.12	REB: 157.20	CON: -205.29	WORK: 212.08

Case 1-Repolymerization (section 3) (EU): NREU 2.78625 MJ / kg amorphous PET resin in forms of chips; GWP 0.24773 kg $CO_{2\text{-eq}}$ / kg amorphous PET resin in forms of chips;

Case 1-Repolymerization (section 3) (CN): NREU 5.4764 MJ / kg amorphous PET resin in forms of chips; GWP 0.6324 kg $CO_{2\text{-eq}}$ / kg amorphous PET resin in forms of chips;

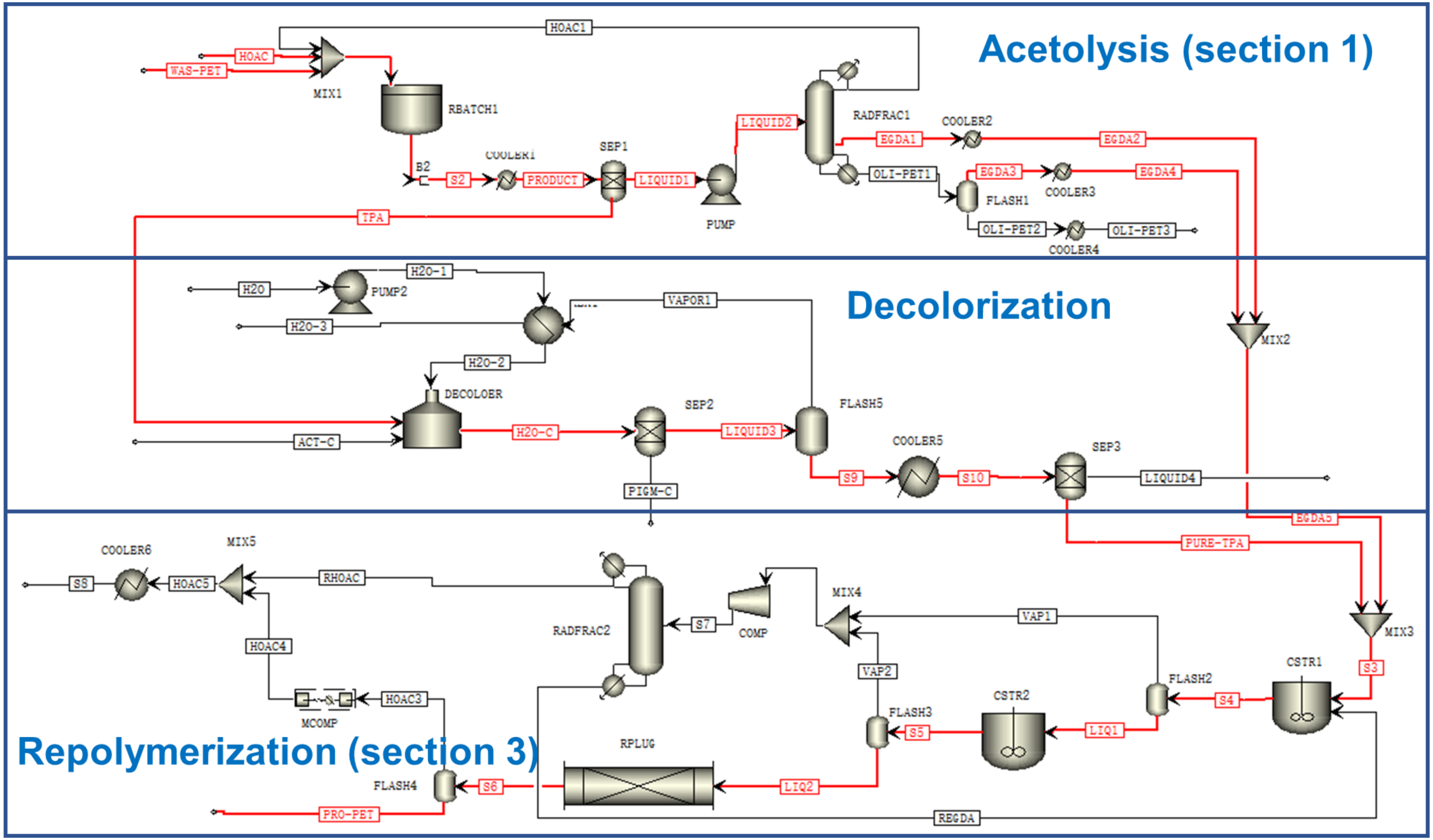

Supplementary Figure 50 Flowsheet diagram from Aspen model for Case 2 (Textiles)

Supplementary Table 33 Mass balance of Case 2 (textiles)

Para.	HOAC	WAS-PET	HOAC1	S2	PRODUCT	LIQUID1	TPA	LIQUID2	EGDA1	OLI-PET1	EGDA2	EGDA3	EGDA4	OLI-PET2	OLI-PET3	EGDA5
Temp./°C	25.00	25.00	118.23	280.00	30.10	30.00	30.00	30.00	216.28	216.28	193.90	224.69	35.00	224.69	35.00	188.65
Pres./kPa	101.33	101.33	102.00	3240.00	105.00	105.00	105.00	121.59	122.27	122.27	116.52	10.00	101.33	10.00	101.33	101.33
W-PET	0	12500.00	0	69.45	69.45	69.45	0	69.45	6.78E-22	69.45	6.78E-22	1.77E-76	1.77E-76	69.45	69.45	6.78E-22
EGDA	0	0	17.24	9470.47	9470.47	9470.47	0	9470.47	9356.84	96.39	9356.84	93.77	93.77	2.62	2.62	9450.61
TPA	0	0	0	10746.30	10746.30	0	10746.30	0	0	0	0	0	0	0	0	0
HOAC	7812.20	0	54525.90	54569.10	54569.10	54569.10	0	54569.10	43.16	0.077	43.16	0.077	0.077	0.00042	0.00042	43.24
H2O	0	0	0	0	0	0	0	0	0	0	0	0	0	0	0	0
PET	0	0	0	0	0	0	0	0	0	0	0	0	0	0	0	0
BAET	0	0	0	0	0	0	0	0	0	0	0	0	0	0	0	0
CARBON	0	0	0	0	0	0	0	0	0	0	0	0	0	0	0	0

Supplementary Table 33 continued

Para.	H2O	H2O-1	H2O-2	ACT-C	H2O-3	VAPOR1	H2O-C	PIGM-C	LIQUID3	S9	S10	LIQUID4	PURE-TPA
Temp./°C	25.00	25.32	156.13	25.00	199.01	200.47	280.00	280.00	280.00	200.47	35.00	35.00	35.00
Pres./kPa	101.33	1215.90	1215.90	101.33	1519.88	1519.88	6280.80	6280.80	6280.80	1519.88	1418.55	1418.55	1418.55
W-PET	0	0	0	0	0	0	0	0	0	0	0	0	0
EGDA	0	0	0	0	0	0	0	0	0	0	0	0	0
TPA	0	0	0	0	1.28	1.28	10746.3	53.73	10692.6	10691.3	10691.3	0	10691.3
HOAC	0	0	0	0	0	0	0	0	0	0	0	0	0
H2O	53741	53741	53741	0	16389.60	16389.60	53741	0	53741	37351.4	37351.4	37351.4	0
PET	0	0	0	0	0	0	0	0	0	0	0	0	0
BAET	0	0	0	0	0	0	0	0	0	0	0	0	0
CARBON	0	0	0	537.41	0	0	537.41	537.41	0	0	0	0	0

Supplementary Table 33 continued

Para.	S3	REGDA	S4	VAP1	LIQ1	S5	VAP2	LIQ2	S6	VAP3	S7	RHOAC	HOAC3	HOAC4	PRO-PET	HOAC5	S8
Temp./°C	117.66	198.80	260.00	251.32	251.32	260	260	260	280	251.01	255.21	118.03	264.41	260.15	264.41	118.04	118.03
Pres./kPa	101.33	121.59	810.60	506.62	506.62	101.33	101.33	101.33	0.13	101.33	111.458	101.33	0.13	101.33	0.13	101.33	101.33
W-PET	6.78E-22	0	6.78E-22	0	0	0	0	0	0	0	0	0	0	0	0	0	0
EGDA	9450.61	1292.73	1865.70	782.45	1083.25	757.26	522.00	235.26	0	1304.46	1304.46	11.73	0	0	0	11.73	11.73
TPA	10691.30	18.40	1148.18	7.18	1141.00	537.33	11.22	526.10	0.0011	18.40	18.40	2.17E-50	0.0009	0.0009	5.50E-05	0.0009	0.0009
HOAC	43.24	4.14E-10	5837.10	4441.45	1395.65	1911.85	1737.79	174.06	1544.86	6179.24	6179.24	6179.24	1544.66	1544.66	0.20	7723.90	7723.90
H2O	0	0	0	0	0	0	0	0	0	0	0	0	0	0	0	0	0
PET	0	0	11786.00	1.56E-76	11786.00	12501.00	6.17E-76	12501.00	12447.2	7.73E-76	7.73E-76	0	5.47E-73	5.47E-73	12447.2	5.47E-73	5.47E-73
BAET	0	38.16	897.45	9.65	887.80	586.21	28.51	557.70	2.12	38.16	38.16	6.48E-33	2.09	2.09	0.033	2.09	2.09
CARBON	0	0	0	0	0	0	0	0	0	0	0	0	0	0	0	0	0

Supplementary Table 34 Utility of Case 2 (textiles)

Unit processes used	Type of Utility	Quantity	Function
RBATCH1	Electricity	503.00 kwh	Heating
COOLER1	U-2	12140.20 kg/h	Cool the product and co-produce steam for RADFRAC1
PUMP1	Electricity	0.34 kwh	Increase the pressure of the fluid
RADFRAC1	U-1; U-3	U-1: 390509 kg/h;<br>U-3: 21679.00 kg/h	U-1: Cool the top fraction;<br>U-3: Heat the tower kettle;
COOLER2	U-1	51608 kg/h	Cooling
FLAH1	U-3	16.58 kg/h	Heating
COOLER3	U-1	1010.24 kg/h	Cooling
COOLER4	U-1	17.78 kg/h	Cooling
PUMP2	Electricity	21.88 kwh	Increase the pressure of the fluid
DECOLOR	Electricity	13290.50 kwh	Heating
COOLER5	U-4	12263.2 kg/h	Cooling the product and co-produce steam
CSTR1	Electricity	7818.82 kwh	Heating
CSTR2	Electricity	771.70 kwh	Heating
RPLUG	Electricity	1145.60 kwh	Heating
COMP	Electricity	19.98 kwh	Increase the pressure of the fluid
RADFRAC2	U-1; U-3	U-1: 77085.7 kg/h;<br>U-3: 363.74 kg/h	U-1: Cool the top fraction;<br>U-3: Heat the tower kettle;
MCOMP	U-1; Electricity	U-1: 15921.3 kg/h<br>Electricity: 272.87 kwh	U-1: Compressor interstage cooling;<br>Electricity: Increase the pressure
COOLER6	U-1	14439.2 kg/h	Cooling

**Supplementary Table 35** Details of the utilities of Case 2 (textiles)		
Type of utility used	Initial and final state of utility	
	Initial	Final
U-1	20 °C; 1 atm; liquid phase	35 °C; 1 atm; liquid phase
U-2	20 °C; 1 atm; liquid phase	250 °C; 39.2 atm; gaseous phase
U-3	250 °C; 39.2 atm; gaseous phase	249 °C; 38.6 atm; liquid phase
U-4	20 °C; 1 atm; liquid phase	175 °C; 8.8 atm; gaseous phase
Electricity	-	

Supplementary Table 36-I Energy balance of case 2 (textiles)

Equipment	RBATCH1	COOLER1	SEP	PUMP1	RADFRAC1		COOLER2	FLASH1	COOLER3	COOLER4
Cost/kwh	503.00	-9156.56	-2.49	0.34	CON: -6790.87	REB: 10353.70	-897.45	7.92	-17.57	-0.31

Case 2-Acetolysis (section 1) (EU): NREU 2.39115 MJ / kg amorphous PET resin in forms of chips; GWP 0.16259 kg $CO_{2\text{-eq}}$ / kg amorphous PET resin in forms of chips;

Case 2-Acetolysis (section 1) (CN): NREU 2.61320 MJ / kg amorphous PET resin in forms of chips; GWP 0.21545 kg $CO_{2\text{-eq}}$ / kg amorphous PET resin in forms of chips;

Supplementary Table 36-II continued

Equipment	PUMP2	DECOLOER	COOLER5
Cost/kwh	21.88	13290.50	-9148.91

Case 2-Decolorization (EU): NREU 1.60776 MJ / kg amorphous PET resin in forms of chips; GWP 0.22277 kg $CO_{2\text{-eq}}$ / kg amorphous PET resin in forms of chips;

Case 2-Decolorization (CN): NREU 6.50874 MJ / kg amorphous PET resin in forms of chips; GWP 0.88181 kg $CO_{2\text{-eq}}$ / kg amorphous PET resin in forms of chips;

Supplementary Table 36-III continued

Equipment	CSTR1	FALSH2	CSTR2	FLASH3	COMP	RPLUG	RADFRAC2		FLASH4	M-COMP		COOLER6
Cost/kwh	7817.82	0	771.70	0	19.98	1145.60	CON: -1340.50	REB: 17.72	0	CON: -276.83	REB:272.87	-251.06

Case 2-Repolymerization (section 3) (EU): NREU 3.80016 MJ / kg amorphous PET resin in forms of chips; GWP 0.33821 kg $CO_{2\text{-eq}}$ / kg amorphous PET resin in forms of chips;

Case 2-Repolymerization (section 3) (CN): NREU 7.48789 MJ / kg amorphous PET resin in forms of chips; GWP 0.86535 kg $CO_{2\text{-eq}}$ / kg amorphous PET resin in forms of chips;

Supplementary Table 37 Material and energy input-output of extrusion

Item	Quantity	Unit
Input:		
PET resin	1031	kg
electricity, low voltage	447	kwh
Heat (from natural gas)	252	MJ
Output:		
amorphous PET resin in forms of chips	1000	kg

Transparent bottles:

Case 1 (EU): NREU 2.50504 MJ / kg amorphous PET resin in forms of chips; GWP 0.2129 kg $CO_{2\text{-eq}}$ / kg amorphous PET resin in forms of chips;

Case 1 (CN): NREU 4.55719 MJ / kg amorphous PET resin in forms of chips; GWP 0.51005 kg $CO_{2\text{-eq}}$ / kg amorphous PET resin in forms of chips;

Case 2 (EU): NREU 2.50504 MJ / kg amorphous PET resin in forms of chips; GWP 0.2129 kg $CO_{2\text{-eq}}$ / kg amorphous PET resin in forms of chips;

Case 2 (CN): NREU 4.55719 MJ / kg amorphous PET resin in forms of chips; GWP 0.51005 kg $CO_{2\text{-eq}}$ / kg amorphous PET resin in forms of chips;

Textiles:

Case 1 (EU): NREU 2.53455 MJ / kg amorphous PET resin in forms of chips; GWP 0.21545 kg $CO_{2\text{-eq}}$ / kg amorphous PET resin in forms of chips;

Case 1 (CN): NREU 4.59241 MJ / kg amorphous PET resin in forms of chips; GWP 0.51360 kg $CO_{2\text{-eq}}$ / kg amorphous PET resin in forms of chips;

Case 2 (EU): NREU 2.53455 MJ / kg amorphous PET resin in forms of chips; GWP 0.21545 kg $CO_{2\text{-eq}}$ / kg amorphous PET resin in forms of chips;

Case 2 (CN): NREU 4.59241 MJ / kg amorphous PET resin in forms of chips; GWP 0.51360 kg $CO_{2\text{-eq}}$ / kg amorphous PET resin in forms of chips;

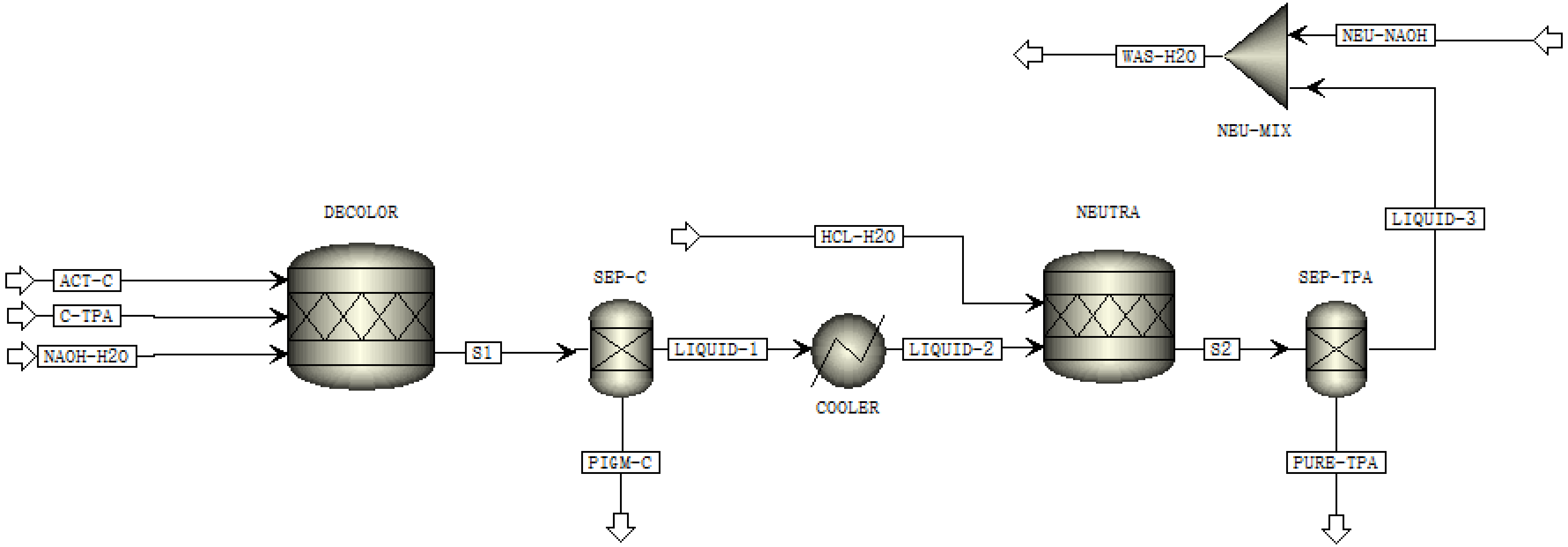

Supplementary Figure 51 Flowsheet diagram from Aspen model for Method 1 (decolorization in alkali)

Supplementary Table 38 Mass balance of method 1 (decolorization in alkali)

Para.	ACT-C	C-TPA	NAOH-H2O	S1	PIGM-C	LIQUID-1	LIQUID-2	HCL-H2O	S2	PURE-TPA	LIQUID-3	NEU-NAOH	WAS-H2O
Temp./°C	25	25	25	100	100	100	30	25	44.39	44.39	44.39	25	45.33
Pres./kPa	100	100	100	100	100	100	100	100	100	100	100	100	100
H2O	0	0	30	30.62	0	30.62	30.62	1.84	33.27	0	33.27	0.00032	33.33
TEREP-01	0	3	0	0.12	0.12	0	0	0	2.88	2.88	0	0	0
1:4-B-01	0	0	0	0	0	0	0	0	0	0	0	0	0
NAOH	0	0	0	0	0	0	0	0	0	0	0	0	0
CARBO-01	0.03	0	0	0.03	0.03	0	0	0	0	0	0	0	0
HCL	0	0	0	0	0	0	0	9.45E-11	8.59E-16	0	8.59E-16	0	4.56E-21
H3O+	0	0	1.37E-11	1.37E-11	0	1.37E-11	1.96E-11	0.79	0.036	0	0.036	1.22E-27	1.71E-.07
NA+	0	0	0.91	0.91	0	0.91	0.91	0	0.91	0	0.91	0.043	0.96
NACL(S)	0	0	0	0	0	0	0	0	0	0	0	0	0
CL-	0	0	0	0	0	0	0	1.48	1.48	0	1.48	0	1.48
OH-	0	0	0.68	0.86	0	0.86	0.86	1.99E-20	7.56E-13	0	7.56E-13	0.032	1.68E-07
TEREP--	0	0	0	2.85	0	2.85	2.85	0	0	0	0	0	0

Description of several components：

Name	Molecular formula	Chemical name
TEREP-01	$C_8H_6O_4$	P-phthalic acid
1:4-B-01	$C_8H_4Na_2O_4$	Terephthalic Acid Disodium Salt
CARBO-01	C	Active carbon
TEREP--	$C_8H_4O_4^{2-}$	Terephthalic anion

Supplementary Table 39 Utility of method 1 (decolorization in alkali)

Unit processes used	Type of Utility	Quantity	Function
DECOLOR	Electricity	2.57 kwh	Heating
COOLER	U-1	36.79 kg/h	Cooling

Supplementary Table 40 Details of the utilities of method 1 (decolorization in alkali)

Type of utility used	Initial and final state of utility	
	Initial	Final
U-1	20 ℃; 1 atm; liquid phase	80 ℃; 1 atm; liquid phase
Electricity	-	

Supplementary Table 41 Energy balance of method 1 (decolorization in alkali)

Equipment	DECOLOER	SEP-C	NEUTRA	SEP-TPA	NEU-MIX	COOLER
Cost/kwh	2.57	0	0	0	0	-2.56

Method 1: NREU 16 MJ / kg pure TPA; GWP 1.39 kg $CO_{2\text{-eq}}$ / kg pure TPA;

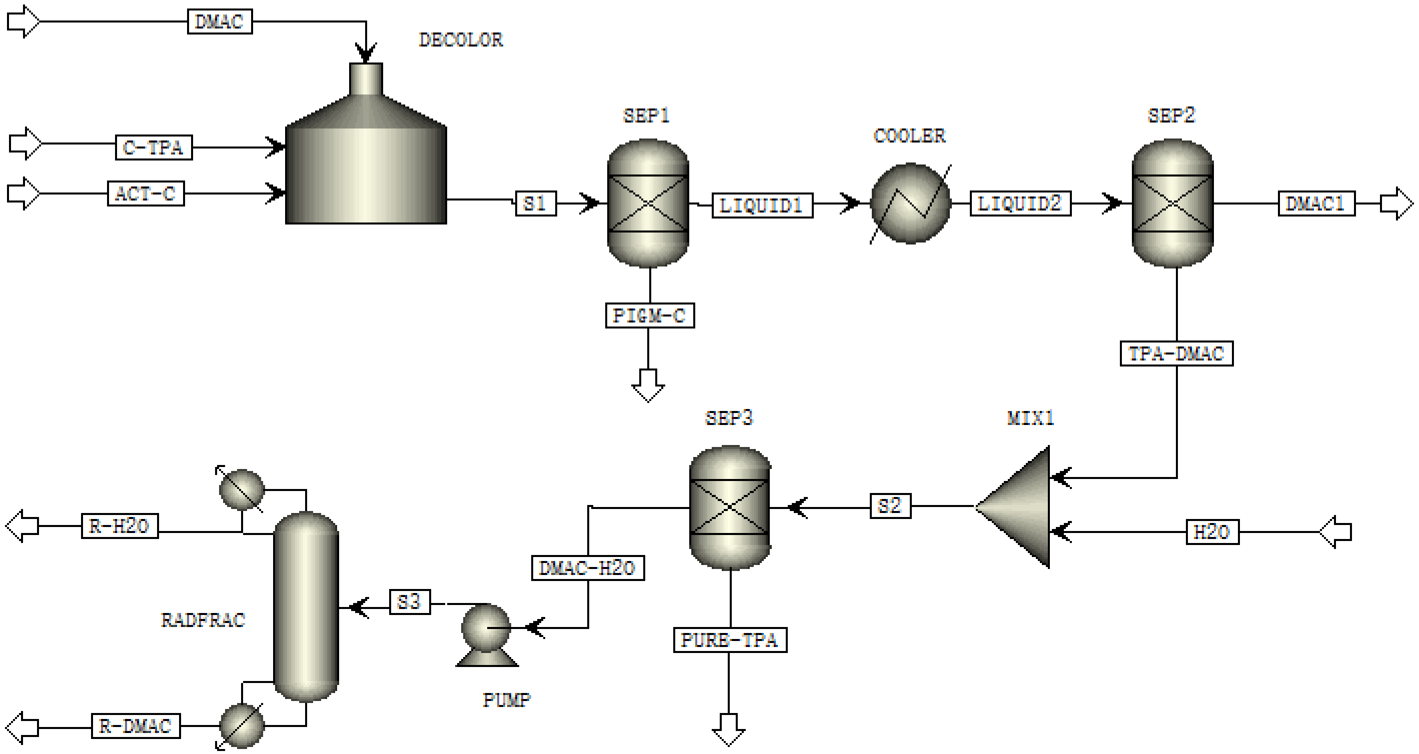

Supplementary Figure 52 Flowsheet diagram from Aspen model for Method 2 (decolorization in DMAc)

Supplementary Table 42 Mass balance of method 2 (decolorization in DMAc)

Para.	ACT-C	DMAC	C-TPA	S1	PIGM-C	LIQUID1	LIQUID2	DMAC1	TPA-DMAC	H2O	S2	PURE-TPA	DMAC-H2O	S3	R-H2O	R-DMAC
Temp./°C	25	25	25	150	150	150	0	0	0	25	24.66	24.66	24.66	24.66	99.68	167.1
Pres./kPa	100	100	100	100	100	100	100	100	100	100	100	100	100	120	100	120
H2O	0	0	0	0	0	0	0	0	0	6	6	0	6	6	5.99	0.0062
TEREP-01	0	0	1.2	1.2	0.06	1.14	1.14	0	1.14	0	1.14	1.14	0	0	0	0
N:N-D-01	0	6	0	6	0	6	6	4.5	1.5	0	1.5	0	1.5	1.5	0.0025	1.50
CARBO-01	0.012	0	0	0.012	0.012	0	0	0	0	0	0	0	0	0	0	0

Description of several components：

Name	Molecular formula	Chemical name
TEREP-01	$C_8H_6O_4$	P-phthalic acid
1:4-B-01	$C_8H_4Na_2O_4$	Terephthalic Acid Disodium Salt
N:N-D-01	C_4H_9NO	N,N-Dimethylacetamide
CARBO-01	C	Active carbon

**Supplementary Table 43** Utility of method 2 (decolorization in DMAc)			
Unit processes used	Type of Utility	Quantity	Function
DECOLOR	Electricity	0.48 kwh	Heating
COOLER	Electricity	0.58 kwh	Refrigeration
PUMP	Electricity	4.95E-5 kwh	Increase the pressure of the fluid
RADFRAC	U-1; U-2	U-1:63.27 kg/h<br>U-2:10.64 kg/h	U-1: Cool the top fraction;<br>U-2: Heat the tower kettle;

**Supplementary Table 44** Details of the utilities of method 2 (decolorization in DMAc)		
Type of utility used	Initial and final state of utility	
	Initial	Final
U-1	20 °C; 1 atm; liquid phase	80 °C; 1 atm; liquid phase
U-2	250 °C; 39.2 atm; gaseous phase	249 °C; 38.6 atm; liquid phase
Electricity	-	

**Supplementary Table 45** Energy balance of method 2 (decolorization in DMAc)									
Equipment	DECOLOR	SEP1	COOLER	SEP2	MIX1	SEP3	PUMP	RADFRAC	
Cost/kwh	0.48	0	-0.58	0	0	0	4.95E-5	CON:-4.41	REB:5.08

Method 2: NREU 28 MJ / kg pure TPA; GWP 2.08 kg $CO_{2\text{-eq}}$ / kg pure TPA;

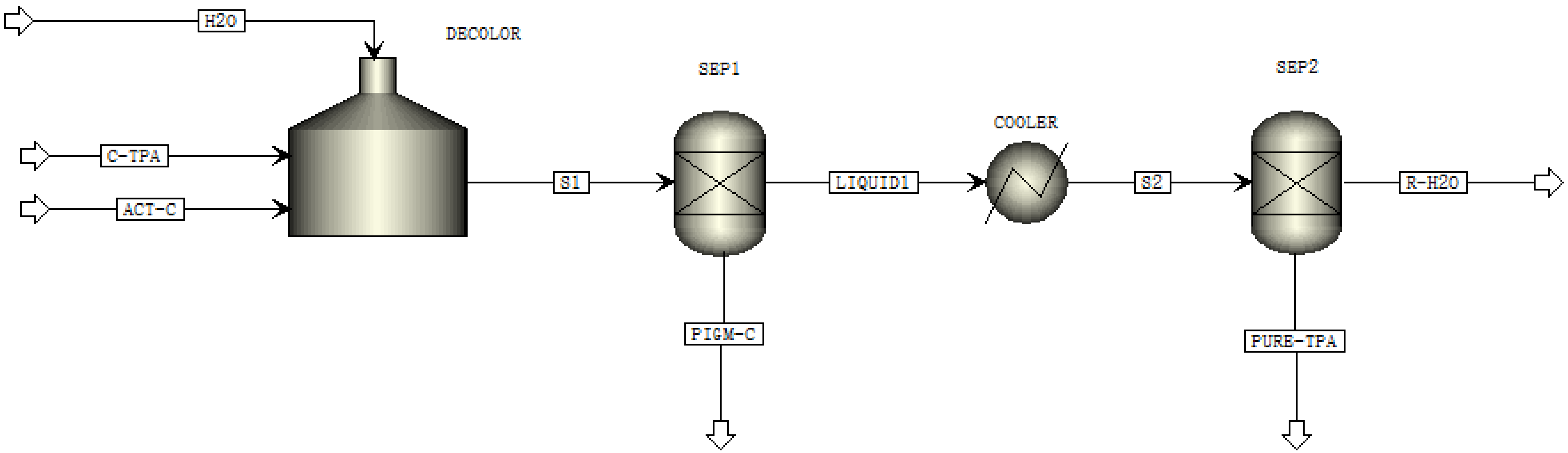

Supplementary Figure 53 Flowsheet diagram from Aspen model for Method 3 (decolorization in hot water)

Supplementary Table 46 Mass balance of method 3 (decolorization in hot water)

Para.	ACT-C	C-TPA	H2O	S1	PIGM-C	LIQUID1	S2	PURE-TPA	R-H2O
Temp./°C	25	25	25	280	270	270	30	30	30
Pres./kPa	100	100	100	6280.8	100	5401.52	100	100	100
H2O	0	0	6	6	0	6	6	0	6
TEREP-01	0	1.2	0	1.2	0.096	1.104	1.104	1.104	0
CARBO-01	0.06	0	0	0.06	0.06	0	0	0	0

Description of several components：

Name	Molecular formula	Chemical name
TEREP-01	$C_8H_6O_4$	P-phthalic acid
CARBO-01	C	Active carbon

Supplementary Table 47 Utility of method 3 (decolorization in hot water)

Unit processes used	Type of Utility	Quantity	Function
DECOLOR	Electricity	2.48 kwh	Heating
COOLER	U-1	24.44 kg/h	Cooling

Supplementary Table 48 Details of the utilities of method 3 (decolorization in hot water)

Type of utility used	Initial and final state of utility	
	Initial	Final
U-1	20 ℃; 1 atm; liquid phase	100 ℃; 1 atm; liquid phase
Electricity	-	

Supplementary Table 49 Energy balance of method 3 (decolorization in hot water)

Equipment	DECOLOR	SEP1	COOLER	SEP2
Cost/kwh	2.48	-0.14	-2.29	0

Method 3: NREU 11 MJ / kg pure TPA; GWP 1.02 kg $CO_{2\text{-eq}}$ / kg pure TPA.

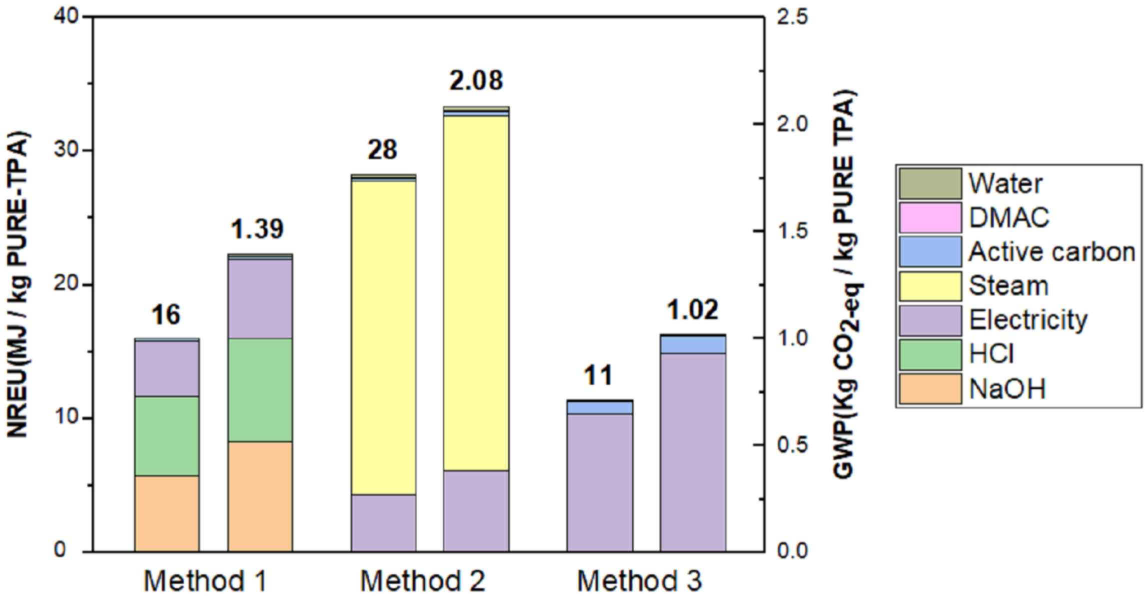

Supplementary Figure 54 LCA results of different decolorization methods. Method 1 is the decolorization of dark TPA in an alkaline solution by activated carbon. Method 2 is the decolorization of dark TPA by activated carbon in DMAc. Method 3 is the decolorization of dark TPA by recrystallization in hot water (280 ºC). These three decolorization treatments are treated as separate units in the process simulation, where heat exchange between material streams is not considered. If the heat exchange is integrated into the entire process, it will consume less energy and have a smaller impact on the environment. The specific process, energy consumption, and material balance of Method 1, Method 2, and Method 3 were described in supplementary Figure 50-52, Supplementary Table 38-49, and supplementary note 5, respectively.

Supplementary References

1 Yoshioka, T., Okayama, N. & Okuwaki, A. Kinetics of hydrolysis of PET powder in nitric acid by a modified shrinking-core model. *Industrial & Engineering Chemistry Research* **37**, 336-340 (1998).

2 Ravens, D. A. S. The chemical reactivity of poly(ethylene terephthalate): Heterogeneous hvdrolysis by hydrochloric acid. Polymer **1**, 375-383 (1960).

3 "Dissociation Constants of Organic Acids and Bases", in CRC Handbook of Chemistry and Physics, Internet Version 2005, <http://www.hbcpnetbase.com>, CRC Press, Boca Raton, FL, 2005.

4 American Society for Testing and Materials. Determination of 4-carboxybenzaldehyde and p-toluic acid in purified terephthalic acid by reverse phase high performance liquid chromatography: ASTM D7884-2020. West Conshohocken: ASTM International, (2020).

5 General Administration of Quality Supervision, Inspection and Quarantine of the People's Republic of China, Standardization Administration of the People's Republic of China. Purified terephthalic acid (PTA) for industrial use: GB/T 32685-2016. Beijing: Standards Press of China, (2016).

6 American Society for Testing and Materials. Specification for purified terephthalic acid (PTA): ASTM D7976-2020. West Conshohocken: ASTM International, (2020).

7 Wang, J. T., Zhou, X. G., Zhao, L., Wang, Y. W., Steady-state simulation of a five-autoclave process polyester industrial plant. *Synthetic Fiber Industry* **35**, 59-63 (2012).

8 Lu, Q. H., Gu, A. J., Zhang, Y. H., & Xie, Z. W., Study and process simulation of polyester polymerization reaction based on Aspen Polymer. *Progress in Chemical Industry* **33**, 1086-1092 (2014).

9 Lu, Q. H., Li, L. J., Zeng, W. B., & Xie, Z. W., Four kettle process modeling for polyester production based on Aspen Polymer. *Synthetic Fiber Industry* **37**, 56-60 (2014).

10 C, K., Y, S. & Liu, A. Step-Growth Polymerization Process Modeling and Product Design. 651-673 (John Wiley & Sons Inc. Press, 2008).

11 Mata-Segreda, J. F. Isergonic relationship in the acid-catalyzed hydrolysis of carboxylic esters with hydrogen-bonding capability. *Journal of Physical Organic Chemistry* **17**, 567-571 (2004).

12 Shen, L., Worrell, E. & Patel, M. K. Open-loop recycling: A LCA case study of PET bottle-to-fibre recycling. Resources, Conservation and Recycling **55**, 34-52 (2010).

13 Gotze, R., Pivnenko, K., Boldrin, A., Scheutz, C. & Astrup, T. F. Physico-chemical characterisation of material fractions in residual and source-segregated household waste in Denmark. *Waste Manag* **54**, 13-26 (2016).

14 Huggins, M. L. Theory of solutions of high polymers. *Journal of the American Chemical Society* **64**, 1712-1719 (1942).

15 Huggins, M. L. Some properties of solutions of long-chain compounds. *J Phys Chem-Us* **46**, 151-158, (1942).
