# Supplementary material for: Acetolysis of waste polyethylene terephthalate for upcycling and life-cycle assessment study
Source: Nat Commun. 2023 Jun 5;14:3249. doi: 10.1038/s41467-023-38998-1 (PMC10241940; doi:10.1038/s41467-023-38998-1)
Supplement: Supplementary file 3 — Description of Additional Supplementary Files [file 41467_2023_38998_MOESM3_ESM.pdf]

Description of Additional Supplementary Files

File Name: Supplementary Movie 1
Description: The supplementary movie shows the depolymerization behavior of waste PET chips in carboxylic acid. In this movie, valeric acid with low saturated vapor pressure instead of acetic acid was used in a pressure glass tube for depolymerization experiments to observe the reaction process conveniently.
